# Quantifying and understanding carbon storage and sequestration within the Eastern Arc Mountains of Tanzania, a tropical biodiversity hotspot

**DOI:** 10.1186/1750-0680-9-2

**Published:** 2014-04-28

**Authors:** Simon Willcock, Oliver L Phillips, Philip J Platts, Andrew Balmford, Neil D Burgess, Jon C Lovett, Antje Ahrends, Julian Bayliss, Nike Doggart, Kathryn Doody, Eibleis Fanning, Jonathan MH Green, Jaclyn Hall, Kim L Howell, Rob Marchant, Andrew R Marshall, Boniface Mbilinyi, Pantaleon KT Munishi, Nisha Owen, Ruth D Swetnam, Elmer J Topp-Jorgensen, Simon L Lewis

**Affiliations:** 1 School of Geography, University of Leeds, Leeds, LS2 9JT UK; 2 School of Biological Sciences, University of Southampton, Southampton, SO17 1BJ UK; 3 Environment Department, University of York, York, YO10 5DD UK; 4 Department of Zoology, University of Cambridge, Cambridge, CB2 3EJ UK; 5 WWF US, Washington, USA; 6 UNEP World Conservation Monitoring Centre, Cambridge, CB3 0DL UK; 7 Genetics and Conservation, Royal Botantic Garden Edinburgh, Edinburgh, UK; 8 Tanzanian Forest Conservation Group, Dar es Salaam, Tanzania; 9 Frankfurt Zoological Society, Frankfurt, D-60316 Germany; 10 The Society for Environmental Exploration, London, EC2A 3QP UK; 11 STEP Program, Princeton University, Princeton, 08544 USA; 12 Department of Geography, University of Florida, PO Box 117315, Gainesville, Florida FL 32611 USA; 13 The University of Dar es Salaam, Dar es Salaam, Tanzania; 14 Centre for the Integration of Research, Conservation and Learning, Flamingo Land Ltd, Malton, YO 17 6UX UK; 15 Sokoine University of Agriculture, PO Box 3001, Morogoro, Tanzania; 16 EDGE of Existence, Conservation Programmes, Zoological Society of London, London, UK; 17 Department of Geography, Staffordshire University, Stoke-on-Trent, ST4 2DF UK; 18 Department of Bioscience, Aarhus University, Aarhus, C DK-8000 Denmark; 19 Department of Geography, University College London, London, WC1E 6BT UK

**Keywords:** Eastern Arc Mountains, Tanzania, IPCC Tier 3, REDD+, Forest, Disturbance, Degradation, Ecosystem service

## Abstract

**Background:**

The carbon stored in vegetation varies across tropical landscapes due to a complex mix of climatic and edaphic variables, as well as direct human interventions such as deforestation and forest degradation. Mapping and monitoring this variation is essential if policy developments such as REDD+ (Reducing Emissions from Deforestation and Forest Degradation) are to be known to have succeeded or failed.

**Results:**

We produce a map of carbon storage across the watershed of the Tanzanian Eastern Arc Mountains (33.9 million ha) using 1,611 forest inventory plots, and correlations with associated climate, soil and disturbance data. As expected, tropical forest stores more carbon per hectare (182 Mg C ha^-1^) than woody savanna (51 Mg C ha^-1^). However, woody savanna is the largest aggregate carbon store, with 0.49 Pg C over 9.6 million ha. We estimate the whole landscape stores 1.3 Pg C, significantly higher than most previous estimates for the region. The 95% Confidence Interval for this method (0.9 to 3.2 Pg C) is larger than simpler look-up table methods (1.5 to 1.6 Pg C), suggesting simpler methods may underestimate uncertainty. Using a small number of inventory plots with two censuses (*n* = 43) to assess changes in carbon storage, and applying the same mapping procedures, we found that carbon storage in the tree-dominated ecosystems has decreased, though not significantly, at a mean rate of 1.47 Mg C ha^-1^ yr^-1^ (c. 2% of the stocks of carbon per year).

**Conclusions:**

The most influential variables on carbon storage in the region are anthropogenic, particularly historical logging, as noted by the largest coefficient of explanatory variable on the response variable. Of the non-anthropogenic factors, a negative correlation with air temperature and a positive correlation with water availability dominate, having smaller p-values than historical logging but also smaller influence. High carbon storage is typically found far from the commercial capital, in locations with a low monthly temperature range, without a strong dry season, and in areas that have not suffered from historical logging. The results imply that policy interventions could retain carbon stored in vegetation and likely successfully slow or reverse carbon emissions.

**Electronic supplementary material:**

The online version of this article (doi:10.1186/1750-0680-9-2) contains supplementary material, which is available to authorized users.

## Background

Tropical forests are globally significant ecosystems; accounting for ~50% of global forest area [1], storing ~ 45% of all carbon in terrestrial vegetation [2–4], maintaining high biodiversity [5], and providing ecosystem services, such as timber, non-timber forest products [6], and climate change mitigation [7, 8]. However, within the last few decades, vast areas of tropical forests have been converted to other land-uses or degraded. For example, between 1990 and 1997, 4.4-7.2 million hectares of humid tropical forest were converted each year and an additional 1.6-3.0 million hectares of forest were visibly degraded [9]. This process increased in the early 2000s, with an estimated 5.1-5.7 million hectares of humid tropical forest (and 3.5-4.7 million hectares of dry tropical forest) deforested per year between 2000 and 2005 [10]. The gradual and sustained reduction in forest quality and quantity has resulted in substantial emissions of CO_2_ [11]. Globally, deforestation and forest degradation accounted for 6-20% of anthropogenic GHG emissions in the 1990s and early 2000s [12–14]. Tropical regions make a substantial contribution to this, emitting 0.7-1.5 Pg C yr^-1^ between 1990 and 1999 [9, 15–17] and 0.71.5 Pg C yr^-1^ between 2000 and 2007 [13, 16–18]. These processes also impact the future potential of forests to remove carbon from the atmosphere [7, 19, 20].

Recently, attempts to mitigate increasing anthropogenic CO_2_ emissions through reducing emissions from degradation and deforestation (REDD+) have been instigated [21]. The REDD+ programme is aimed at contributing to a reduction in greenhouse emissions whilst providing economic incentives for better management and protection of forests. This policy has been widely welcomed and may provide a financial incentive to significantly reduce carbon emissions [22, 23], although the equity and justice issues surrounding the impact on local livelihoods are actively debated [24, 25]. Key technical issues for the successful implementation of REDD+ include (but are not limited to) the accuracy of monitoring systems, preventing leakage and establishing accurate historical baselines. Thus, the success of REDD+, in part, rests on robust scientific information on the magnitude and extent of carbon storage in tropical regions and how it changes over time [26].

The Intergovernmental Panel on Climate Change (IPCC) provide a three “Tier” system through which carbon stocks and emissions can be reported, each with a different level of methodological complexity and accuracy. Tier 1 is the simplest method, using global default values obtained from the IPCC literature [27, 28]. The intermediate Tier 2 level improves on Tier 1 by using country specific data. Tier 3 is the most rigorous approach, using local forest inventory data, focusing on the direct measurement of trees, repeated over a time series [27–29]. Here we develop a Tier 3 methodology for the Eastern Arc Mountains (EAM) watershed area.

The estimates become progressively more robust from Tier 1 to 3 due to changes in two main systematic errors [29]. The first, completeness, refers to the number of IPCC carbon pools that are included, with studies including all five pools (aboveground live, litter, coarse wood debris [CWD], belowground and soil carbon) considered complete. The second, representativeness, derives from the substantial natural variability in the carbon stored across landscapes, even within a biome or country [30]. The aboveground biomass of a forest within a landscape may differ considerably from global default (Tier 1) values or even from country-specific (Tier 2) values. For example, in the Peruvian Amazon, data from the Los Amigos Conservation Concession [31] were shown not to be representative of forests nationally. Nearby forests situated to the north and south of this local study are estimated to contain 20-35% less carbon per unit area [32], suggesting that Los Amigos Conservation Concession is an area of locally high biomass. Since Tier 3 methods account for variation observed within biomes and countries, the representativeness of the carbon estimates is higher than those associated with Tier 1 and 2 methodologies [32, 33].

However, Tier 3 methods are more expensive [34, 35] and some nations may lack the capacity to adopt such methods [36]. Whilst, in some cases, the capability to apply Tier 3 guidelines is being rapidly developed, multi-temporal inventory data and data on historical carbon stock changes can take several decades to accrue [37, 38]. It is expected that REDD+ requirements will allow data provisions from several tiers in a single report. Highly variable and/or substantial carbon pools should be estimated using Tier 3 methodology (e.g. forest aboveground live carbon [ALC]), whilst Tier 1 or Tier 2 methodology may be sufficient for smaller carbon pools (e.g. CWD) or carbon poor land cover categories (e.g. bare ground).

In Tier 3 methods, in order to extrapolate from plot data, it is necessary to develop correlations with remotely sensed data to scale to the study area or country-wide estimates. Generally, carbon storage is either estimated via statistical correlation with electromagnetic properties, ground-truthed by volumetric measurements, such as diameter at breast height (DBH), which are converted to biomass estimates using allometric equations. A variety of remotely sensed data sources have been employed for carbon mapping and these can be aggregated into four groups: photographic imagery, RADAR, LiDAR, and ancillary geographic information systems (GIS) data (see Additional file 1: SI1 for an evaluation of each method). Here, we use ancillary GIS data as such data have three main advantages: 1) wide availability, often free of charge; 2) a suitable resolution (e.g. 90 m [39]); and 3) correlations with these ancillary GIS data may indicate which variables directly affect carbon storage. Developing an understanding of how these variables influence carbon storage is vital for accurate scenarios of future emissions.

**Figure 1:**
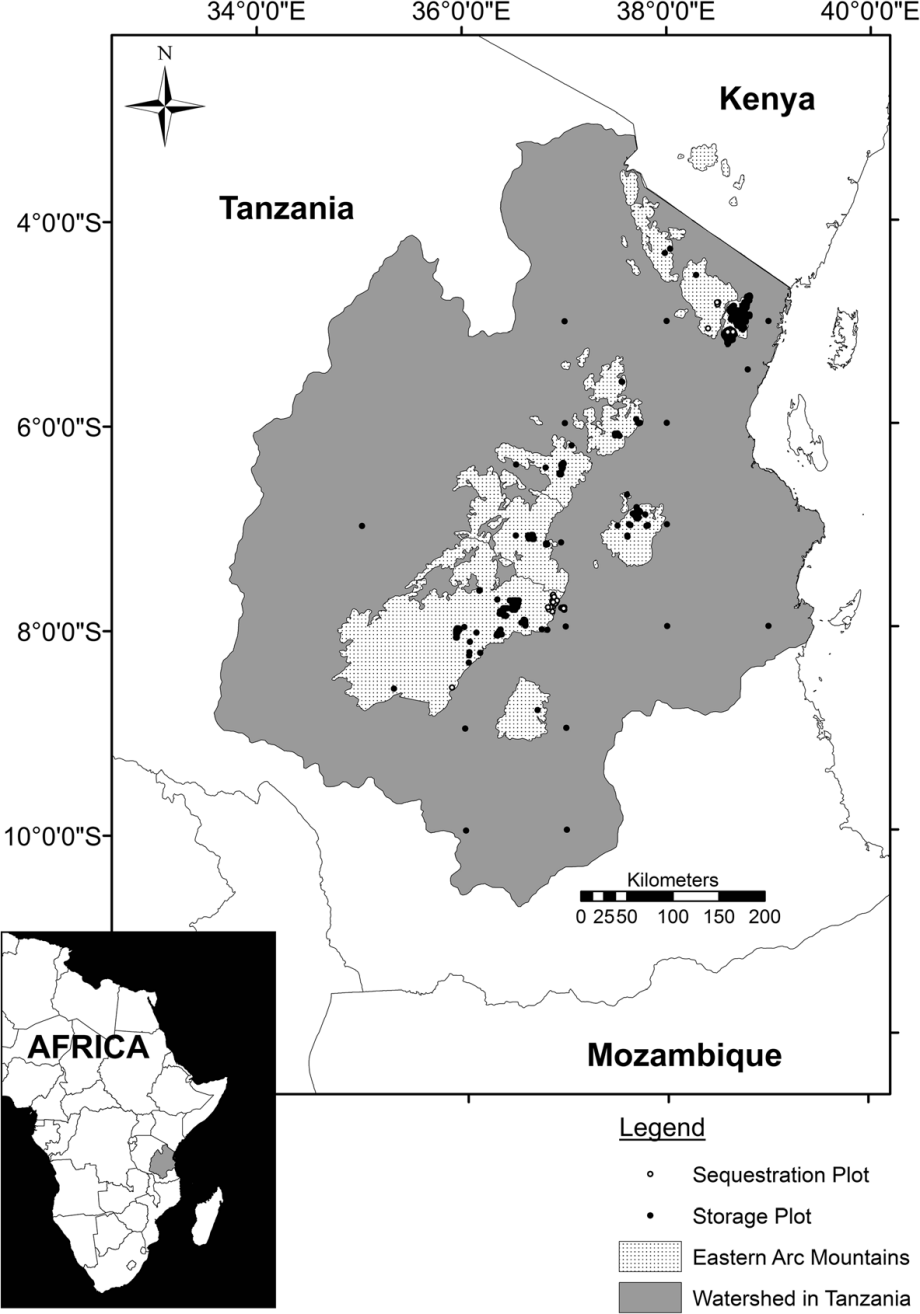
**The Eastern Arc Mountains of Tanzania and Kenya** [40]**.** The study area is the Eastern Arc watershed in Tanzania [41].

Here, we correlate carbon storage estimates from tree inventory plots (*n* = 1,611, median size = 0.1 ha) with data on climatic (e.g. temperature, precipitation, and solar radiation), edaphic (e.g. soil water holding capacity and soil fertility) and proxy variables for direct human interventions (e.g. governance type, distance from the main economic demand centres, population pressure, and historical logging), and variables that derive from climate-human interactions (e.g. burnt area index) for the Tanzanian watershed of the Eastern Arc Mountains (hereafter, EAM [40]), which covers 33.9 million ha (Figure 1; see Swetnam et al (2011) [41] for further details). We develop Tier 3 type correlation equations to estimate the total ALC stored across the forested and wooded land cover categories, an advancement on previous Tier 2 estimates for the region presented in Willcock et al (2012) [42]. Additionally, we investigate the most influential correlates of spatial differences in carbon storage and how these result from changes in either species composition affecting wood density (specific gravity) or the number of large trees present. Lastly, a smaller number of inventory plots (n = 43, median size 0.1 ha) have two censuses, and by applying the same mapping procedures, we assess changes in carbon storage over time, providing a first-order estimate of sequestration across the region.

## Results

### Carbon stocks

**Figure 2:**
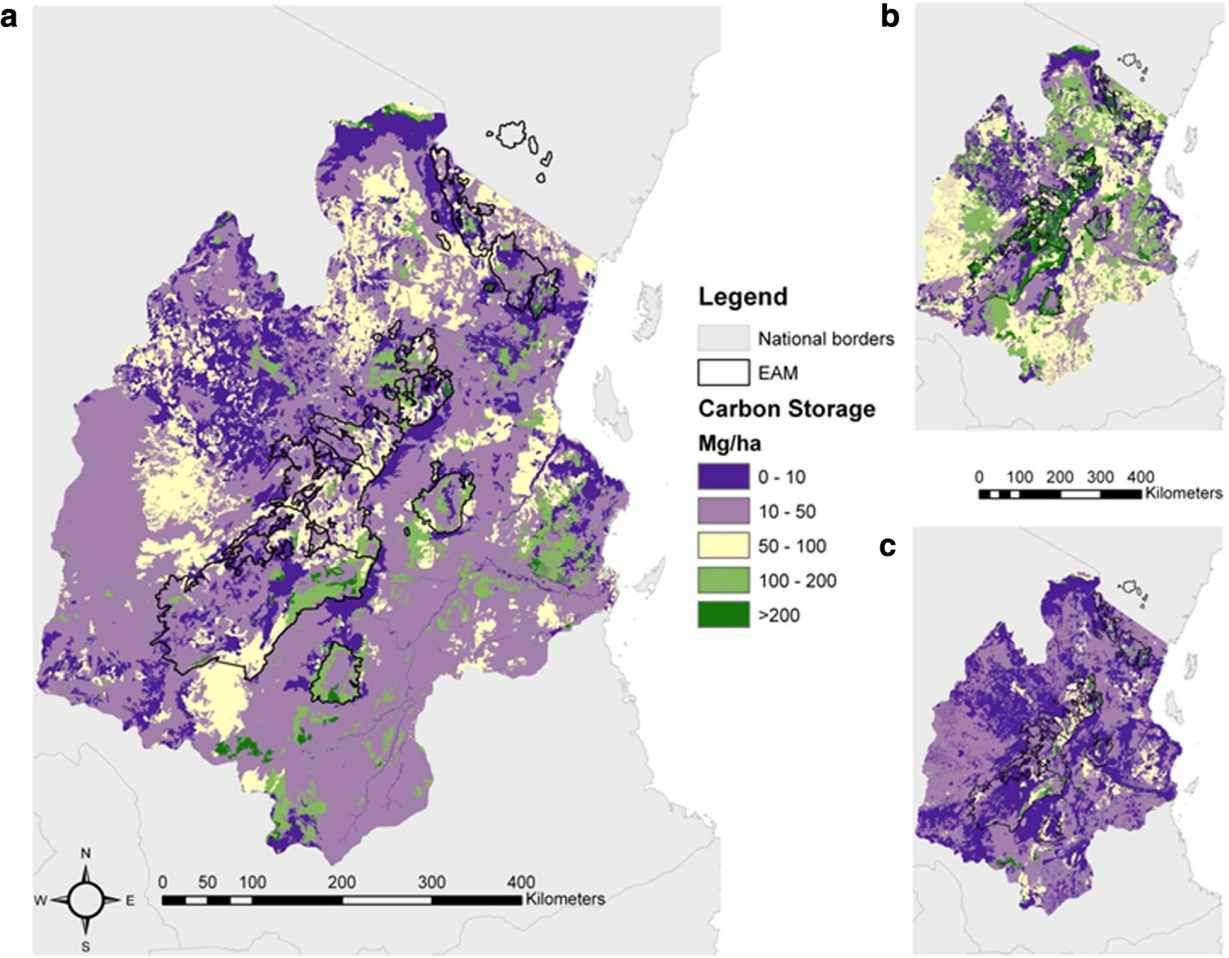
**Aboveground live carbon storage in the study area (a), with upper (b) and lower (c) pixel based 95% CI.** See text for details on Methods.

Utilising 1,611 plots and scaling to the 33.9 million ha study area we estimate that 1.32 (95% confidence interval [CI] ranges from 0.89 to 3.16) Pg C was stored in the aboveground live vegetation in the year 2000 (Figure 2; Table 1). Woodland and bushland contributed most to the amount of stored aboveground live carbon (ALC) in the study region, with open woodland storing the most ALC (0.49 [0.47 to 1.60] Pg C over 9.6 million ha); followed by bushland (0.29 [0.15 to 0.51] Pg C over 5.0 million ha) and closed woodland (0.18 [0.13 to 0.61] Pg C over 1.8 million ha).

**Table 1:** **Aboveground live carbon stored within the study area for the year 2000, estimated by this and previous studies**

Study	Aboveground live carbon, Pg (95% CI range)	Methodology	Resolution (m^2^)	Disturbance included?	Tanzanian on-the-ground data?
Present study* – Tier 3	1.32 (0.89-3.16)	Correlation equations derived using remotely sensed influential variables.	100	Anthropogenic variables represent human disturbance. Natural disturbance variables also included.	Yes
Willcock et al (2012)* – Original Tier 2 [42]	1.58 (1.56-1.60)	Land cover based look-up table.	100	Only where land cover categories are identified as disturbed (e.g. cropland mosaics).	Yes
Willcock et al (2012) – Harmonised Tier 2 [42]	1.64 (1.52-1.76)	Land cover based look-up table.	100	Only where land cover categories are identified as disturbed (e.g. cropland mosaics).	Yes
Baccini et al (2012) – Tier 1 [3]	2.03	Derived from MODIS and GLAS LiDAR data.	500	Partially includes disturbance through impacts on canopy heights.	Yes
Saatchi et al (2011) – Tier 1 [4]	0.83	Derived from MODIS, SRTM, QSCAT and GLAS LiDAR.	1000	Partially includes disturbance through impacts on canopy heights.	No
Hurtt et al (2006) HYDE-SAGE – Tier 1 [46]	0.63	Modelled from the Miami LU ecosystem model with cropland data from the Centre for Sustainability and the Global Environment.	~110,000	Contains simple submodels of natural plant mortality, disturbance from fire, and organic matter decomposition, as well as wood harvesting.	No
Hurtt et al (2006) HYDE – Tier 1 [46]	0.41	Modelled from the Miami LU ecosystem model.	~110,000	Contains simple submodels of natural plant mortality, disturbance from fire, and organic matter decomposition, as well as wood harvesting.	No
Baccini et al (2008) – Tier 1 [47]	0.34	Derived from MODIS and GLAS LiDAR data.	1000	Partially includes disturbance through impacts on canopy heights.	No

*This study and Willcock et al (2012) are not independent as they are derived from the same underlying data and utilise the same look-up table values.

Best estimate values from our methodology, per unit area, in each land cover class, are given in Table 2. Forest contained the greatest ALC per unit area, with highest values in sub-montane forest (189 [95 to 588] Mg ha^-1^), followed by lowland (182 [152- to 360] Mg ha^-1^), upper montane (166 [69 to 533] Mg ha^-1^), montane (130 [62 to 702] Mg ha^-1^), and forest mosaic (121 [55 to 485] Mg ha^-1^). Woodlands held less ALC than forests, with closed woodland storing 100 (70 to 331) Mg ha^-1^ and open woodland storing 51 (38 to 165) Mg ha^-1^ (Table 2), but more than the landscape average of 39 (26 to 93) Mg ha^-1^.

**Table 2:** **The mean (and 95% CI) estimates of forest characteristics investigated in this study (carbon storage, carbon sequestration, WSG, the intercept from the power law relationship and the gradient from the power law relationship) separated by land cover category**

Land cover category [41]	Carbon storage (Mg ha^-1^)	Carbon sequestration (Mg ha^-1^ yr^-1^)	WSG (g cm^-3^)	The intercept from the power law relationship	The gradient from the power law relationship
**Lowland Forest (<1000 m)**	182 (152 to 360)	-0.91 (-7.08 to 4.29)	0.60 (0.59 to 0.60)	6.01 (2.94 to 5.17)	-0.93 (-1.04 to -0.82)
**Sub-montane forest (1000-1500 m)**	189 (95 to 588)	-2.02 (-11.06 to 1.29)	0.58 (0.57 to 0.58)	5.95 (3.68 to 8.23)	-1.31 (-1.48 to -1.14)
**Montane Forest (1500-2000 m)**	130 (62 to 702)	-2.03 (-11.85 to 1.07)	0.60 (0.59 to 0.60)	6.95 (3.51 to 10.39)	-1.57 (-1.82 to -1.32)
**Upper-montane forest (>2000 m)**	166 (69 to 533)	-2.08 (-10.49 to 1.23)	0.60 (0.58 to 0.60)	7.03 (4.60 to 9.45)	-1.61 (-1.93 to -1.26)
**Forest mosaic**	121 (55 to 485)	-1.18 (-6.69 to 2.92)	0.56 (0.56 to 0.56)	9.22 (6.98 to 11.46)	-1.90 (-1.99 to -1.81)
**Closed Woodland**	100 (70 to 331)	-1.24 (-7.91 to 2.63)	0.64 (06.2 to 0.65)	6.67 (4.95 to 8.60)	-1.55 (-1.85 to -1.30)
**Open Woodland**	51 (38 to 165)	-1.49 (-7.53 to 2.05)	0.61 (0.59 to 0.62)	6.38 (4.88 to 7.82)	-1.45 (-1.70 to -1.19)

**Figure 3:**
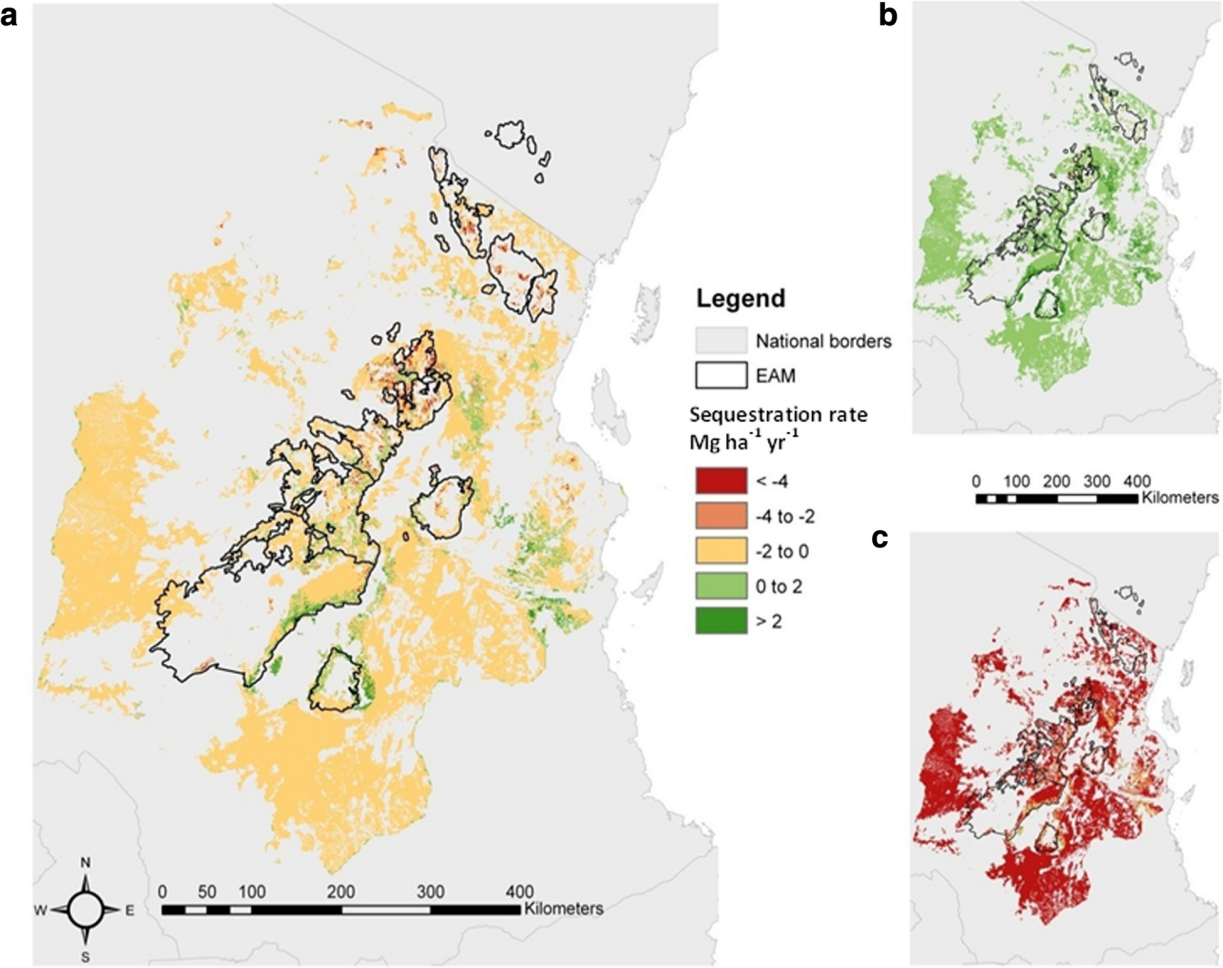
**Aboveground live carbon sequestration in tree-dominated land cover categories within the study area (a), with upper (b) and lower (c) pixel based 95% CI.** See text for details on Methods.

Our sequestration model suggests that the landscape may be losing 0.05 (-0.07 to 0.26) Pg C yr^-1^ (mean net flux to atmosphere of 1.47 [-2.13 to 7.75] Mg C ha^-1^ yr^-1^). Of the 12.3 million ha of tree-dominated land in our study area, only 1.4% (0.17 million ha) shows a carbon decrease over the entire 95% CI range and only 0.8% (0.10 million ha) a definite carbon increase (Figure 3). The locations showing net carbon uptake are in the Udzungwa mountains, while the locations with net reductions in carbon storage are mainly in the Pare and Usambara mountains.

### Links between carbon stock and influential variables

The variables that influence carbon storage and sequestration may be inferred from relationships within the correlation models. Forward selection results are presented in the following paragraphs as these best indicate causal relationships [43–45]. In general, backward models were in close agreement with forward models (Tables 3 and 4; Additional file 1: Tables S1-S3).

**Table 3:** **The coefficients and associated p-values of the variables correlated with aboveground carbon storage using both forward and backward selection procedures**

Variable (where appropriate, units are given in brackets)	Group	Forward		Backward	
		Coefficient	p-value	Coefficient	p-value
**(Intercept)**	n/a	-1.21E + 03	3.14E-03	-2.80E + 00	7.55E-01
**Natural logarithm of the population pressure with decay constant of 12.5 km**	Anthropogenic	1.06E + 00	1.06E-05	1.42E + 00	2.27E-06
**Natural logarithm of the population pressure with decay constant of 16.7 km**	Anthropogenic	n/a	n/a	1.42E + 00	2.27E-06
**Distance to roads** (km)	Anthropogenic	1.15E-04	1.09E-03	1.78E-04	1.30E-05
**Historical logging – Partially logged** (no logging/partially logged)	Anthropogenic	-2.10E + 00	1.09E-03	-3.83E + 00	4.97E-07
**Cost distance to Dar es Salaam**	Anthropogenic	3.41E-05	2.00E-03	2.58E + 00	5.46E-03
**Natural logarithm of the cost distance to market towns**	Anthropogenic	-6.05E-01	5.24E-02	-9.85E-01	1.89E-02
**Governance – local** (national/local/joint/unknown)	Anthropogenic	4.24E + 00	9.29E-03	n/a	n/a
**Governance – national** (national/local/joint/unknown)	Anthropogenic	-7.95E-03	9.78E-01	n/a	n/a
**Governance – unknown** (national/local/joint/unknown)	Anthropogenic	6.26E-01	7.10E-01	n/a	n/a
**Mean annual monthly temperature range** (°C)	Climatic	-9.79E-01	2.00E-16	-1.15E + 00	1.98E-13
**Mean annual minimum monthly temperature** (°C)	Climatic	n/a	n/a	1.09E + 00	3.07E-16
**Mean annual maximum monthly temperature** (°C)	Climatic	n/a	n/a	-1.15E + 00	1.98E-13
**Mean number of dry months annually**	Climatic	-2.28E-01	2.57E-02	-3.09E-01	5.58E-03
**Total available water capacity of the soil** (vol. %, -33 to -1500 kPA conforming to USDA standards)	Edaphic	-3.75E-01	1.16E-05	-8.59E-01	3.05E-05
**Total nitrogen content of the soil** (g kg^-1^)	Edaphic	n/a	n/a	-4.13E-01	2.50E-03
**Total carbon content of the soil** (g kg^-1^)	Edaphic	n/a	n/a	6.18E + 00	1.15E-03
**pH of the soil** (pH)	Edaphic	n/a	n/a	1.73E + 00	2.96E-02
**Spatial autocorrelation term 5**	Spatial	6.45E + 01	3.15E-03	6.60E + 00	1.18E-01
**Spatial autocorrelation term 7**	Spatial	-8.48E-01	3.57E-03	-1.71E-01	1.45E-01
**Spatial autocorrelation term 4**	Spatial	n/a	n/a	6.60E + 00	1.18E-01
**Spatial autocorrelation term 3**	Spatial	n/a	n/a	-1.71E-01	1.45E-01

**Table 4:** **The coefficients and associated p-values of the variables correlated with aboveground carbon sequestration**

Variable	Coefficient	p-value
**(Intercept)**	0.032	0.890
**PC1**	-0.112	0.006
**PC3**	-0.255	0.010
**PC5**	-0.412	0.012

**Figure 4:**
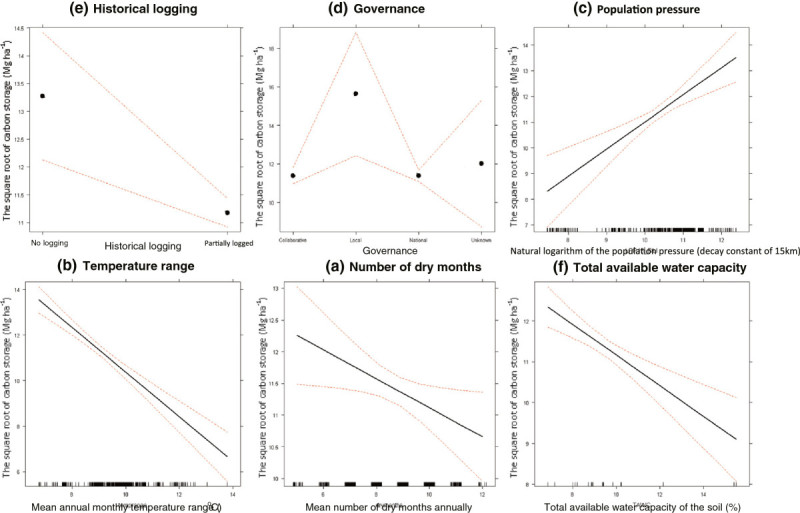
**The modelled effect of most influential, significant anthropogenic (a, b, and c), climatic (d and e) and edaphic (f) variables of aboveground live carbon storage.** Dashed red lines indicate the modelled 95% CI. The data is indicated by black lines above the x-axis.

Carbon storage (adjusted R-squared [Adj R-sq] = 0.18) is correlated positively with the natural logarithm of the population pressure with decay constant of 12.5 km (p-value < 0.001) and increased by 1 Mg ha^-1^ for every 8700 km from a road (p-value < 0.010), and every 30,000 units in the cost distance to Dar es Salaam (p-value < 0.010). Carbon storage decreased by 1 Mg ha^-1^ for every 1°C increase in mean annual monthly temperature range (p-value < 0.001), every 2.7% rise in the total available water capacity of the soil (p-value < 0.001), and every 4.4 month increase in the mean number of dry months annually (p-value < 0.050). Carbon storage was 2.1 Mg ha^-1^ lower in areas where historical logging was present (p-value < 0.010), and 4.2 Mg ha^-1^ higher in areas under the control of local communities/governments (p-value < 0.010). Thus, carbon storage is high in areas far from the commercial capital, with a low monthly temperature range, without a dry season, that have not suffered from historical logging and are under local community/government control (Figure 4; Table 3).

The rate of carbon sequestration correlated with three principal component (PC) axes (presented in order of influence; Adj R-sq = 0.41). Carbon sequestration was negatively correlated with the soil fertility axis (PC5; p-value < 0.050), warmer temperatures and longer dry seasons (PC3; p-value < 0.050), and with increased anthropogenic disturbance (PC1; p-value < 0.010). Thus, carbon sequestration was highest in less fertile areas with little or no drought and little anthropogenic disturbance (Table 4).

Wood specific gravity (WSG; Adj R-sq = 0.28; see Additional file 1: SI2) was most strongly affected by the annual mean burned area probability (increasing by 1 g cm^-3^ for every 0.04 increase; p-value < 0.001) and the total available water capacity of the soil (decreasing by 1 g cm^-3^ for every 82.0% increase; p-value < 0.001). Thus, WSG is higher in burnt areas with little available water (Additional file 2: Figure S1; Additional file 3: Figure S2; Additional file 1: Table S1).

The intercept of the power law relationship (an indication of potential stem density [see Additional file 1: SI3]; Adj R-sq = 0.30) was most affected by the natural logarithm of the population pressure with decay constant of 12.5 km (positive correlation; p-value < 0.001) and the mean annual monthly temperature range (increasing by 1.0 for every 1.2°C increase; p-value < 0.001). Thus, the density of smaller stems increases in areas with a high population pressure and large temperature fluctuations (Additional file 3: Figure S2; Additional file 4: Figure S3; Additional file 1: Table S2).

Correlations identified for the gradient of the power law relationship (an indication of the proportion of larger stems; see Additional file 1: SI3) were broadly the inverse of those identified for the intercept. The gradient of the power law relationship was most affected by the natural logarithm of the population pressure with decay constant of 20.8 km (negative correlation; p-value < 0.001) and the mean burned area probability in the fourth quarter (decreasing by 1.0 for every 0.2 increase; p-value < 0.001). Thus, the proportion of large stems was greater in areas experiencing few disturbances from people or fire (Additional file 3: Figure S2; Additional file 5: Figure S4; Additional file 1: Table S3).

When investigating the most influential correlates of spatial differences in carbon storage and how these result from changes in either species composition affecting wood density (specific gravity) or the number of large trees present, we found that the final Tier 3 carbon storage estimates were positively correlated with both size-frequency distribution estimates (both intercept and gradient [p-values < 0.001]), and negatively correlated with WSG estimates (p- value < 0.001) and maximum height estimates (p-value < 0.001; Additional file 1: see SI4). All possible interactions were investigated and were significant (Adj R-sq = 0.35; p- values < 0.001), however, the majority of the explanatory power lay within the second order interactions (Adj R-sq = 0.33; p-values < 0.001; Additional file 1: Table S5). Broadly, WSG and the proportion of larger stems had largest influence over the carbon storage estimate. Considering only second order interactions, in areas of low potential stem density, carbon storage is positively correlated with maximum canopy height (Additional file 6: Figure S5). However, the opposite correlation is observed in areas of higher stem density. Although similar interactions are observed between both size-frequency distribution estimates (gradient and intercept), the interaction between WSG and maximum canopy height is inverse, with carbon storage only showing positive correlations with maximum canopy height in areas of high WSG. Both size-frequency distribution estimates also interacted similarly with WSG, with both showing positive correlations with carbon storage in areas of low WSG, but negative correlations in areas of high WSG (Additional file 6: Figure S5). Finally carbon sequestration correlation values were positively correlated with carbon storage estimates (p-value < 0.001), indicating that areas storing the most carbon are also those that are increasing in stock at the fastest rate.

## Discussion

### Tier 3 correlation-based method vs. Tier 1 and 2 methods

Our estimates of 1.3 Pg C stored across the 33.9 million hectares is larger than most previous Tier 1 estimates [46–48], although below the most recently produced estimate [3] (Table 1). Underestimation of the amount of carbon stored in the EAM region in global analyses can be a result of their poor resolution and/or application of data from other regions which may differ systematically compared to East African forests, woodlands and savannas [42]. When separated by land cover category, our locally derived carbon estimates are comparable to those presented in other local [49–52] and global studies, the latter often containing little or no data from East Africa [3, 4, 46, 47, 53]. This suggests differences between our estimates and other studies have arisen because many previous studies mapped carbon storage at lower resolution [3, 4, 46, 47, 53]. When considering homogenous landscapes, scale effects are unlikely to cause a dramatic difference in carbon estimates. However, in highly fragmented and heterogeneous landscapes, such as East Africa, the effects of scale are likely to be substantial. Forest fragments, typically of high carbon storage, may be omitted at lower resolutions, being ‘replaced’ by more dominant, but low carbon, land cover categories (e.g. open woodland), resulting in underestimation of carbon storage.

It must be noted that, the landscape-scale confidence intervals surrounding our Tier 3 estimates are considerably wider than those around previous estimates [3, 4, 42, 47, 53]. This result is consistent with Hill et al (2013), who also showed increasing methodological sophistication does not necessarily result in reduced uncertainty, as is often assumed [54]. Confidence intervals derived from look-up table values may show a systematic bias. The ranges provided are an artefact of the study area, the number of land cover categories and the resolution, as when summed across a large number of pixels, pixel error is mostly negated as underestimates in one part of the landscape are counterbalanced by overestimates in other parts. The 95% CI developed from correlation equations are effectively based on numerous continuous variables, containing the uncertainty relating to anthropogenic, climatic and edaphic variables, thus have many thousands of possible combinations, severely limiting the ability of the ‘law of averages’ to act. Hence, the 95% CI presented in this investigation may better reflect that of the actual landscape, containing more variables that make-up the complex landscape heterogeneity (i.e. improved representativeness), although this is only true for those pixels estimated using the correlation equations (86% of the EAM but only 52% of the study area). Therefore, the look-up table 95% CI presented in Willcock et al (2012), and used in this study, may underestimate uncertainty [42]. Future studies should expand the existing plot network (Figure 1), enabling the correlation equations (and improved 95% CI) to be applied to the entire study area. This process has already begun under a new WWF-REDD+ project (which focusses on better sampling the data-deficient land cover categories identified in this study [55]) and the National Forest Monitoring and Assessment (NAFORMA) project [56, 57].

### Links between carbon stock and influential variables

The results presented here indicate that ALC storage in tree-dominated ecosystems is correlated with anthropogenic, climatic and edaphic variables. However, in all our models there is a large amount of unexplained variation (R-squared values for our correlation models vary between 0.18 and 0.41). This is likely to be due to three main reasons (Additional file 1: SI6). Firstly, although we used the highest resolution datasets that are freely available, several of the associated variables are of relatively poor resolution across the EAM (including; wind, light and soil nutrient variables [Additional file 1: Table S6]). This is particularly important here as low resolution GIS data is unlikely to correlate well with the response variables from our plot network as many plots (with high variance [58]) may fall within a single cell [59]. Thus, our study may be biased against retaining low resolution explanatory variables in our models. Secondly, contemporary forest characteristics are the result of growth, recruitment and mortality over many years. It is difficult to obtain data on historical variables and yet these could have had a significant impact on present day carbon storage and other forest characteristics [60]. Thirdly, present day information is also lacking, for example datasets describing physical soil properties in the study area are unavailable. Thus, future work is needed to develop additional high resolution GIS data, particularly for historic time periods.

Of the variance explained in our forward and backward models, direct anthropogenic factors are the most influential explanatory variables (as noted by the largest coefficient of explanatory variables on the response variable, in contrast to those [e.g. temperature] with smaller p-values but also smaller influence [Table 3]) and so are the focus of our remaining discussion (see Additional file 1: SI5 for discussion of climatic and edaphic variables).

Within our study area, people are clustered around high carbon areas (Figure 4). We suggest this could be due to these areas having favourable climatic conditions with more moisture for plant (and thus crop) growth. Further, the incidence of malaria is lower at high elevations [61], making these locations more habitable for human populations. Thus there is a peak in population density near the base of high-carbon montane forests [40]. Our interpretation that it is the landscape suitability driving human population density is consistent with the observation that when individual localities are followed over time, degradation at the local level caused by the population is evident [62, 63]. This emphasises that our results are not proof of causation and that the drivers may be a correlate of the explanatory variables retained in our models (Additional file 1: SI6). Our results also show a decrease in carbon storage in previously logged areas and in areas nearer the commercial capital, Dar es Salaam. This confirms previous reports that areas near the capital have lower biomass due to the local demand of low grade timber by the city, as well as international demand for high grade timber via the city’s port [62]; emphasising the connections between the rural and urban landscape, and how the sphere of urban influence drives change in rural ecosystems. Future investigations should use simulation modelling and direct experimentation to identify if the influential variables highlighted here can be confirmed as drivers of carbon storage and sequestration, providing a deeper understanding of the process-based relationships.

The decrease in carbon storage as a result of logging (51-77% of the ALC is retained) is of similar magnitude to other reported estimates [64]. However, the historical logging data we utilised was based on expert opinion (Additional file 1: Table S6) so, given its importance, further work developing and evaluating historical variables is needed (Additional file 1: Table S7). We observe a comparable decrease due to differing governance. Land under national control holds between 40% and 65% of the ALC stored in areas under decentralised governance. This perhaps indicates that decentralisation of management (e.g. participatory and community led forestry) is successful in our study area [37, 65]. However, it is not possible to prove causation within the framework of this study. Many locally managed forests are located in the south-east of our study area within an area of naturally high carbon storage, whereas land under national control covers much larger areas, including the dry, carbon-poor east. Hence, our finding that carbon storage is higher in areas under decentralised control may be an artefact of the differing areas where this type of land management occurs. Further studies monitoring change in carbon storage over time under the two different governance regimes would enable the effect of land management to be determined.

The overall effects on carbon storage are a result of many changes in forest characteristics. Both WSG and the proportion of larger stems decrease with increasing anthropogenic disturbance, however, stem density (≥ = 10 cm DBH) increases. Anthropogenic disturbance, for example logging, is often a commercial activity and results in the preferential removal of the largest, most valuable stems [62]. The more open canopy, following stem removal, would result in increased recruitment from young forest trees [66], leading to the high numbers of small stems observed. However, the opposite would be expected in woodlands and savannas, with more open canopies resulting in more grass, high fire intensity and so less recruitment [67, 68]. Our results highlight how influential the negative effect of people on tropical forest carbon storage can be. This assertion is supported by data from across the tropics [69–71]. The significant impact of anthropogenic activities implies that REDD+ could, at the local scale, have significant positive impacts on carbon storage. However, careful policy designs to limit leakage of deforestation and encourage the involvement of the local population are needed to ensure REDD+ schemes achieve their carbon storage and sequestration aims [72].

Like carbon storage and its components, carbon sequestration is also correlated with anthropogenic, climatic and edaphic variables. We estimate that some localities (for example the Udzungwa Mountains National Park; Figure 4) provide a carbon sink of comparable per-area magnitude to modelled estimates in East Africa [73] and to that observed over recent decades in structurally intact African forest [7]. However, many areas of forest and woodland within the study area experience a high level of degradation and disturbance, and so are net sources. Here, we have shown that anthropogenic disturbance is a key determinant of the trend in carbon storage over time in eastern Tanzania. Important locations of high carbon losses are the Pare and Usambara mountains (Table 5), which historically have seen the highest rates of degradation and disturbance [74]. The national population of Tanzania is increasing [75] and this may increase the pressure on tree-dominated ecosystems which could result in the study area becoming a significant source of carbon in the future. Furthermore, the effect of increase in anthropogenic pressures could be compounded by potential decrease in carbon storage as a result of increasing temperatures [76, 77] and changes in soil nutrients (see Additional file 1: SI5). However, these future effects could be complicated by increasing levels of atmospheric CO_2_, varying effectiveness of legally protected areas and shifting consumption patterns.

**Table 5:** **Carbon stored and sequestered across the individual mountain blocks of the EAM range (the total is denoted in bold)**

Eastern Arc Mountain Block [40]	Area, km^2^	Aboveground live carbon storage, Tg		Mean carbon sequestration, Mg ha^-1^ yr^-1^
		Tier 3	Willcock et al (2012) - Original Tier 2 [42]	
North Pare	510	1.93	2.38	2.60
South Pare	2,327	8.96	9.59	2.41
West Usambara	2,945	13.52	15.96	3.64
East Usambara	1,145	5.91	7.63	2.79
Nguu	1,562	9.34	12.71	1.89
Nguru	2,565	15.11	18.86	1.79
Ukaguru	3,243	13.39	20.63	1.42
Uluguru	3,057	15.92	13.91	1.35
Rubeho	7,984	36.84	40.96	1.06
Malundwe	33	0.29	0.29	1.80
Udzungwa	22,788	101.73	104.05	1.01
Mahenge	2,606	23.58	12.08	0.19
**Total**	**50,765**	**246.53**	**259.06**	**1.19**

## Conclusions

Our results show that the amount of carbon stored in forests across 33.9 million ha of the Eastern Arc Mountains of Tanzania is considerable: 1.32 (0.89 to 3.16) Pg. Our estimate is significantly higher than most previous estimates. However, our more sophisticated method also has higher uncertainty, implying that other methods may substantially underestimate the uncertainty involved. Within the tree-dominated land cover categories, historical logging is the most influential direct anthropogenic factor, while the mean number of dry months is the most influential environmental factor, with an order of magnitude less impact on carbon storage. We show that WSG, size-frequency distribution variables and height variables are all important in determining carbon storage. Our estimates indicate that, between 2004 and 2008, tree-dominated communities across the study areas showed no significant change, however some areas were identified as large sinks (0.8% of the study area) and others large sources (1.4% of the study area), showing the importance of taking a landscape scale approach. The carbon maps produced and statistical relationships documented can assist policy-makers in designing policies to maintain and enhance carbon storage for climate mitigation and other ecosystem services.

## Method

We collated data from 2,462 tree inventory plots within our study area (see Additional file 1: SI3), then applied a quality control and standardisation protocol. This consists of two main steps: (1) Metadata quality control; and (2) Measurement bias detection.

Firstly, all plots lacking a recorded spatial location and a fixed area were discarded (770 plots). Plots where one or more diameter at breast height (DBH) data were known to be missing were also excluded (7 plots). Furthermore, plots smaller than 0.025 ha (16 plots) were deemed to produce unreliable carbon estimates so also removed from the dataset.

Secondly, to assess possible measurement bias, i.e. not measuring over buttresses and so overestimating biomass [78], the remaining plots were grouped by the lead field researcher. Size-frequency distributions, using 10 cm size classes, were created for each of these groups. Forest size-frequency distributions are suggested to conform to the -2 power law based on metabolic scaling [79]. Although it has been argued that this rule is not globally applicable [80], many studies accept this as a theoretical maximum value for the abundance of large stems [81]. Thus, researchers with many plots above this maximum value likely measured stems around buttresses and so were removed (1 researcher, 100 Plots).

The quality control and standardisation procedure resulted in a dataset of 1,611 tree inventory plots (median 0.1 ha, mean 0.1 ha, mode 0.1 ha [43 plots with multiple censuses; median 0.1 ha, mean 0.5 ha, mode 1.0 ha]; Figure 1; see Additional file 1: SI3 for a further information) from which we calculated plot-level stand structure indices and aboveground carbon storage per unit area (see Additional file 1: SI2 for full details). We obtained the exponent and intercept of the population size-frequency distribution using the power law fit for each plot using the log-log transformation method. Whereby, for each plot, we created 10 cm bin size-frequency distributions based on DBH, and a linear model of the logarithm of frequency against the logarithm of the size class was fitted. Whilst not as accurate as the maximum likelihood estimation method, our simpler method is more stable for many of our plots, providing both the intercept and slope indicators of population structure [82].

We obtained WSG data via the phylogenetic information provided by our tree inventory plots. We used a global wood density database to extract species average WSG [83]. This procedure provided over 32,000 trees with WSG data. When this was not possible we adopted a hierarchical approach, first applying the appropriate genus average if available (~14,000 trees) before considering family average (~9,500 trees), plot average (~4,500 trees) and dataset average (~80 trees) in turn [84]. Including WSG as an additional parameter in allometric equations reduces the biomass estimation error [49, 85, 86].

In addition, we estimated plot biomass using moist forest tree allometry [86] based on measurements of DBH from our tree inventory plots, WSG (as described above) and height data (derived from our dataset using the best fit DBH-height equation form [Equation 5.1; see Additional file 1: SI4], if not measured in the tree inventory plots). Finally, carbon was assumed to be 50% of biomass [7].

For a smaller number of plots, multiple measurements were available over time (n = 43; mean plot size = 0.5 ha; mean measurement period = 3.9 years). We calculated changes in carbon storage rates by dividing the difference in carbon storage estimates between censuses by the number of years separating them.

For our 1,611 geo-referenced tree inventory plots, we obtained further information on variables falling into five broad categories; anthropogenic, climatic, geographic, edaphic, and pyrologic (median resolution 1.0 ha, mean resolution 22.0 ha, mode resolution 1.0 ha; Additional file 1: Table S6). Anthropogenic data, further divided into six subcategories, were obtained: (1) population pressure variables (n = 14 related variables) were obtained from Platts (2012) [87] (see Additional file 1: SI7); (2) Dar es Salaam related variables (n = 3; e.g. distance to Dar es Salaam), (3) market town related variables (n = 3; e.g. distance to market towns), and (4) infrastructure related variables (n = 2; e.g. distance to roads) were derived from available topographic maps; (5) historical logging (n = 1) from Swetnam et al (2011) [88]; and (6) governance (n = 1) from the World Database on Protected Areas [89]. Climate data were divided into three subcategories (precipitation [n = 2; maximum mean cumulative water deficit and mean number of dry months annually], temperature [n = 4; mean annual temperature, mean annual minimum monthly temperature, mean annual monthly maximum temperature, and mean annual monthly temperature range] and wind speed [n = 1]) and were derived from the Tropical Rainfall Measuring Mission [90, 91], WorldClim [92, 93], and United States National Aeronautics and Space Administration Surface meteorology and Solar Energy [94] datasets. Similarly, geographic data have two variables (aspect [n = 1] and incoming solar radiation [n = 1]) derived from Shuttle Radar Topography Mission [93] and National Renewable Energy Laboratory [95, 96] datasets respectively. Lastly, we extracted edaphic data (n = 6) from the International Soil Reference and Information Centre database [97, 98] and fire-related variables (n = 5) derived from MODIS images [99].

We then correlated these variables with carbon storage, and following this, its components: WSG, the intercept of the power law relationship, and the gradient of the power law relationship, in each case using general linear models (see Additional file 1: SI2-5). No transformations were required to ensure a normal distribution when correlating either WSG, the intercept of the power law relationship or the gradient of the power law relationship with the individual variables. However, carbon storage estimates required a square root transformation to ensure a normal distribution within the general linear models (normality was confirmed using the Shapiro-Wilk test; p-value > 0.05). In all models, plots were weighted by the square root of their area as confidence in biomass estimation increases with the area surveyed [100, 101]. Landscape scale spatial autocorrelation was accounted for by including spatial terms (latitude, longitude and the interactions between them) in the model (Additional file 1: Table S6) [102]. The numerous possible interactions were excluded from the models, as these were found to add very little explanatory power to the models, only increasing R-squared values by ~0.001 with the addition of each interaction term. All analyses were performed using R 2.12.1 [103] and mapped in ArcGIS v9.3.1 [104].

When assessing carbon sequestration (n = 43) fewer degrees of freedom were available, therefore explanatory variables need to be grouped. Therefore, we conducted a principle components (PC) analysis, obtaining five PC which explained >90% of the cumulative variance of the individual influential variables (Additional file 1: Table S4). Then, covariation of PC with carbon sequestration was assessed instead of the individual influential variables. Carbon sequestration estimates required a cube-root transformation to ensure a normal distribution within the general linear models (confirmed using the Shapiro-Wilk test; p-value > 0.05). This enabled the effect of multiple variables to be examined even with this limited dataset. PC analysis of the variables was performed on the scaled data using the prcomp package [105] within R 2.12.1 [103]. All other aspects of the model (weighting and spatial autocorrelation) were performed identically to the models for carbon storage and its components.

The most appropriate model was chosen using forward and backward stepwise selection. Forward models are more useful for inferring causal relationships [43] and so were preferentially used to infer the influential variables of carbon storage and sequestration. However, averaging forward–backwards and backward–forwards predictions outperforms conventional selection procedures [43] and so both methods were used when estimating the spatial distributions within the study area. Akaike information criterion (AIC) was used to reduce/expand the models, with variable selection occurring when the variable reduced the mean squared error (MSE) under ten-fold cross validation [106]. Unlike model selection using R-squared, which neglects the principles of parsimony, AIC considers both model fit and complexity, resulting in better predictions and allowing inferences to be made from multiple models [107]. Model selection continued until the addition/removal of further variables able to reduce cross validation MSE no longer increased AIC, thereby producing the best-fit model with the lowest prediction error [43].

Within each category (anthropogenic, climatic, geographic, edaphic, and pyrologic), some variables were highly correlated (Additional file 1: Table S7) and this may confound the stepwise procedure as each variable does not carry enough distinct information [108]. For example, all temperature related variables (Additional file 1: Table S7) were correlated (R-squared > 0.6). However, it is unclear which correlated best with the variables of interest, e.g. carbon storage and sequestration. Many studies include mean annual temperature in biomass models [77, 109], but theory suggests that it may be the temperature range driving this relationship as photosynthesis correlates with maximum temperatures, but respiration with minimum temperatures [76, 110, 111]. We found that, if we removed correlated variables prior to model selection, the final models were artefacts of the variables we had selected. For example, if we included mean annual temperature in the model, but not temperature range, then the significant correlations between mean annual temperature and ALC storage were found. However, these correlations were insignificant if temperature range was added to the model, with the newly added variable showing a significant effect instead. In short, the resultant models were automatically biased towards *a priori* expectations. To avoid this bias, we devised a procedure by which the influential variables included in model selection were selected by their ability to explain variation within the data of interest (e.g. carbon storage). All variables (describe above) were included in model selection. Once this had run to completion the model was assessed. The subcategory with the most correlated variables retained within the model was selected and all but the most influential, significant variable were removed. For example, if all four temperature-related variables were included in the initial model and this was the largest group of variables then this group would be selected. Then, if mean annual temperature was the most influential and significant temperature-related variable, all other temperature-related variables would be excluded in the next round of model selection. Thus, stepwise model selection was then repeated for all remaining variables. This process was repeated until no highly correlated variables remained within the model produced.

Since only landscape-scale variation was accounted for by the spatial terms already included in the model (latitude, longitude and the interactions between them; Table 1; Additional file 1: Table S6), it was necessary to investigate the effect of local-scale (<10 km^2^) spatial autocorrelation [102]. To do this, the separate forward and backward models, containing no highly correlated variables (produced above), were mapped. Then, the sum of the model estimates within the maps were extracted at 1, 3, 5, 7 and 10 km^2^ resolutions, and included as additional variables (representing local spatial autocorrelation terms) into the stepwise model selection process, which was re-run a final time [112]. However, in all cases, local spatial autocorrelation terms were rejected as they did not reduce cross validated MSE.

Since it was not necessary to include local spatial autocorrelation terms in the models, the preliminary maps produced above could be regarded as final spatial representations of the ten best fit models, two (forward and backward) for each of the five variables of interest (carbon storage, carbon sequestration, WSG, the intercept of the power law relationship and the gradient of the power law relationship). Each pair of maps (forward and backward) were then combined into a single, final weighted mean estimate. The ratio of the relevant cross validated MSE of the forward and backward models was used to create the weighted mean, with the model showing lowest error receiving the highest weighting [43]. Thus, we ultimately produced five maps (from ten best fit models); one each for carbon storage, carbon sequestration, WSG, the intercept of the power law relationship, and the gradient of the power law relationship. As our carbon storage estimates were derived from data representing trees with a DBH greater than or equal to 10 cm, regionally estimates of ratios from Willcock et al (2012) were used to estimate the unmeasured component of ALC storage [42], this was summed with our modelled carbon storage estimate, providing an estimate of total ALC storage.

Although the five maps produced covered the entire study area, we were concerned that extrapolating predictions beyond the range of observed predictor variables from our dataset could result in large, unquantifiable errors. Thus, we limited the models to localities where all the associate variables were within the range of that shown in our dataset, thus only interpolating within our correlation models for tree-dominated land cover categories. For any pixels outside the data range, look-up table methods were used in preference to the correlation model estimates. Thus, for every land cover in our study area containing trees (open woodland; closed woodland; forest mosaic; lowland forest; sub-montane forest; montane forest; and upper montane forest [41]) that fell within the limits of our dataset, the estimate of carbon storage derived from the correlation equations was used. For all other land cover categories, and for those localities for which predictor variables fell outside the ranges of values used in model construction, land cover based look-up table values from Willcock et al (2012) were used to estimate ALC storage [42]. In total, look-up table values were applied to 52% of the landscape, although this was predominantly to low carbon land cover categories, with 86% of the EAM (which hold the majority of the regions tropical forest [113]) estimated using the correlation approach described above. Estimates of WSG and population structure were only made for wooded land cover categories, with estimates for areas within our dataset range being derived from the relevant correlation equations and estimates for other areas coming from land cover based look-up table values derived from the median value of our WSG and population structure data (weighted by the square root of plot size and derived via sampling with replacement 10,000 times) for each land cover category (Additional file 1: Table S8). For carbon sequestration, again, estimates were only made for wooded land cover categories for those areas inside the range of our dataset estimates derived from the correlation equations were used. However, unlike carbon storage, WSG and population structure, for areas outside the range of our dataset, a land cover based look-up table was not used as several land cover categories were poorly represented due to the small sample size available (n = 43). Instead, for pixels outside the range of the correlation-derived carbon sequestration model (16% of pixels with wooded land cover), the median value of data from our recensused plots (again weighted by the square root of plot size and derived via sampling with replacement 10,000 times) was utilised.

For every 1 ha pixel of each map derived from correlation equations, we produced 95% confidence intervals (CI). If the pixel estimate was derived from the general linear models, then the pixel 95% CI was calculated by adding and subtracting the square root of the cross validation MSE. For look-up table pixels the look up table 95% CI were used. The pixel 95% CI describes, for every pixel, the range we would expect each of our estimates to lie within. However, as we are also interested in estimating carbon storage and sequestration on a landscape scale, indications of uncertainty are also required at landscape-scale. Simply summing the pixel 95% CI to derive 95% CI of the overall landscape-scale estimates would incorrectly treat random error as a region-wide systematic bias. Thus, to derive 95% CI for landscape-scale estimates, we randomly allocated each pixel an estimate within the range dictated by its 95% pixel CI, and summed these values across the entire landscape. This process was performed 10,000 times and the median value and 95% CI (the 250^th^ and 9,750^th^ ranked values, which may not be equally distributed around the median) for aboveground carbon storage and sequestration in the study area were obtained.

For the final model of carbon storage estimates, we investigated how the components of carbon storage (population structure, WSG and tree height) interacted to ultimately produce the ecosystem service of carbon storage. We obtained estimates of maximum canopy height from the best fit DBH-height equation [Equation 5.1; see Additional file 1: SI4 and Additional file 7: Figure S6], and combined this spatially with our correlation model derived estimates of WSG, the intercept of the power law relationship and gradient of the power law relationship. We then correlated these against our estimates of carbon storage, allowing all possible interactions, and selected the best-fit model (via AIC) using both forwards and backwards stepwise regression.

Ethical approval for the above study was obtained from the Faculty of Environment Research Ethics Committee, in accordance with the University of Leeds research ethics policy.

## Electronic supplementary material

Additional file 1: Supporting text (including S1-7 and Tables S1-12). (DOCX 398 KB)

Additional file 2: Figure S1: The spatial variation of WSG in tree-dominated land cover categories within the study area (a), with upper (b) and lower (c) pixel based 95% CI. See text for details on methods. (DOC 410 KB)

Additional file 3: Figure S2: The most influential, significant influential variables on WSG (a and b), the intercept of the power law relationship (c and d), and the gradient of the power law relationship (e and f). Dashed red lines indicate 95% CI. (DOC 77 KB)

Additional file 4: Figure S3: The spatial variation in the intercept of the power law relationship (a proxy measure for potential stem density) in tree dominated land cover categories within the study area (a), with upper (b) and lower (c) pixel based 95% CI. See text for details on methods. (DOC 273 KB)

Additional file 5: Figure S4: The spatial variation in the gradient of the power law relationship (a proxy measure for the proportion of larger stems) in tree-dominated land cover categories within the study area (a), with upper (b) and lower (c) pixel based 95% CI. See text for details on methods. (DOC 254 KB)

Additional file 6: Figure S5: The 2^nd^ order interactions relating my carbon storage derivatives (wood specific gravity, maximum canopy height, the intercept of the power law relationship, and the gradient of the power law relationship [shown here as WSG, height, intercept, and gradient respectively]) to aboveground live carbon storage. Dashed red lines indicate 95% CI. (DOC 91 KB)

Additional file 7: Figure S6: The effect of MAT on tree height for a range of DBH. The data (points) correspond to DBH ranges whereas the Gompertz model fits (solid lines) illustrate the relationship for mid-point of this range only. Dotted lines represent the 95CI of the model fits. (DOC 122 KB)

Below are the links to the authors’ original submitted files for images.

Authors’ original file for figure 1

Authors’ original file for figure 2

Authors’ original file for figure 3

Authors’ original file for figure 4

Authors’ original file for figure 5

Authors’ original file for figure 6

Authors’ original file for figure 7

Authors’ original file for figure 8

Authors’ original file for figure 9

Authors’ original file for figure 10

## Abbreviations

AIC: Akaike information criteria
ALC: Aboveground live carbon
CI: Confidence interval
CV: Cross validation
CWD: Coarse woody debris
DBH: Diameter at breast height
EAM: Eastern Arc Mountains
eCEC: Effective cation exchange capacity
GIS: Geographic information systems
HYDE: History Database of the Global Environment
IPCC: Intergovernmental Panel on Climate Change
IUCN: International Union for Conservation of Nature
KITE: York Institute for Tropical Ecosystems
MAT: Mean annual temperature
MSE: Mean squared error
PC: Principal components
REDD+: Reducing Emissions from Deforestation and Forest degradation
SAGE: Centre for Sustainability and Global Environment
WSG: Wood specific gravity.
