# Correction to: Quantifying and understanding carbon storage and sequestration within the Eastern Arc Mountains of Tanzania, a tropical biodiversity hotspot

**DOI:** 10.1186/s13021-017-0088-7

**Published:** 2017-12-07

**Authors:** Simon Willcock, Oliver L. Phillips, Philip J. Platts, Andrew Balmford, Neil D. Burgess, Jon C. Lovett, Antje Ahrends, Julian Bayliss, Nike Doggart, Kathryn Doody, Eibleis Fanning, Jonathan M. H. Green, Jaclyn Hall, Kim L. Howell, Rob Marchant, Andrew R. Marshall, Boniface Mbilinyi, Pantaleon K. T. Munishi, Nisha Owen, Ruth D. Swetnam, Elmer J. Topp-Jorgensen, Simon L. Lewis

**Affiliations:** 1 School of Geography, University of Leeds, Leeds, LS2 9JT UK; 2 School of Biological Sciences, University of Southampton, Southampton, SO17 1BJ UK; 3 Environment Department, University of York, York, YO10 5DD UK; 4 Department of Zoology, University of Cambridge, Cambridge, CB2 3EJ UK; 5 WWF US, Washington, USA; 6 UNEP World Conservation Monitoring Centre, Cambridge, CB3 0DL UK; 7 Genetics and Conservation, Royal Botantic Garden Edinburgh, Edinburgh, UK; 8 Tanzanian Forest Conservation Group, Dar es Salaam, Tanzania; 9 Frankfurt Zoological Society, Frankfurt, 60316 Germany; 10 The Society for Environmental Exploration, London, EC2A 3QP UK; 11 STEP Program, Princeton University, Princeton, 08544 USA; 12 Department of Geography, University of Florida, PO Box 117315, Gainesville, FL 32611 USA; 13 The University of Dar es Salaam, Dar es Salaam, Tanzania; 14 Centre for the Integration of Research, Conservation and Learning, Flamingo Land Ltd, Malton, YO17 6UX UK; 15 Sokoine University of Agriculture, PO Box 3001, Morogoro, Tanzania; 16 EDGE of Existence, Conservation Programmes, Zoological Society of London, London, UK; 17 Department of Geography, Staffordshire University, Stoke-on-Trent, ST4 2DF UK; 18 Department of Bioscience, Aarhus University, Aarhus C, 8000 Denmark; 19 Department of Geography, University College London, London, WC1E 6BT UK

## Correction to: Carbon Balance Manag (2014) 9:2 10.1186/1750-0680-9-2

Upon publication of the original article [1], the authors noticed that the figure labelling for Fig. 4 in the online version was processed wrong. The top left panel should be panel **a**, with the panels to its right being **b** and **c**. **d** and **e** should be the panels on the lower row, and **f** is correct. The graphs themselves are all correct. It is simply the letter labels that are wrong. The panel labels should be a, b, c, d, e, f and not e, d, c, b, a, f.

The corrected Fig. 4 is given in this erratum.

**Fig. 4:**
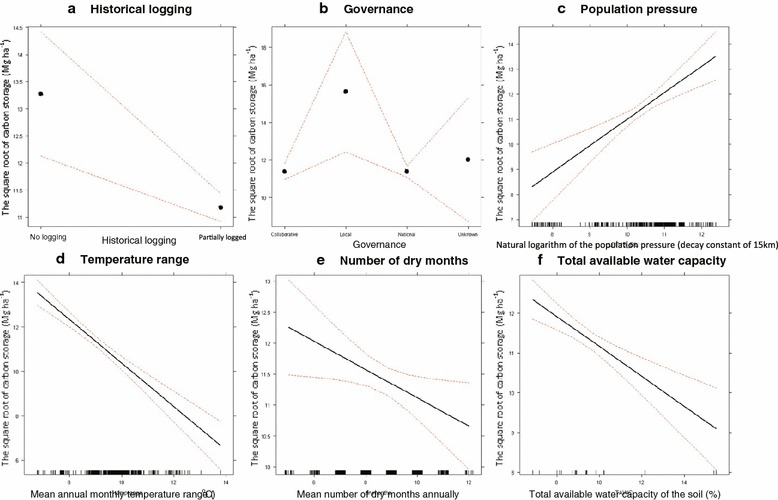
The modelled effect of most influential, significant anthropogenic (**a**−**c**), climatic (**d**, **e**) and edaphic (**f**) variables of aboveground live carbon storage. Dashed red lines indicate the modelled 95% CI. The data is indicated by black lines above the x-axis
