# Supplementary material for: Quantifying and understanding carbon storage and sequestration within the Eastern Arc Mountains of Tanzania, a tropical biodiversity hotspot
Source: Carbon Balance Manag. 2014 Apr 28;9:2. doi: 10.1186/1750-0680-9-2 (PMC4041645; doi:10.1186/1750-0680-9-2)
Supplement: Supplementary file 1 — Additional file 1: Supporting text (including S1-7 and Tables S1-12). (DOCX 398 KB) [file 13021_2013_99_MOESM1_ESM.docx]

**SI1 – REMOTELY SENSED DATA SOURCES ARE EMPLOYED FOR CARBON MAPPING**

A variety of remotely sensed data sources are employed for carbon mapping and these can be aggregated into six groups: very high resolution imagery, moderate resolution data, coarse resolution data, RADAR, LiDAR, and ancillary geographic information systems (GIS) data. Very high resolution imagery (<5m resolution; e.g. IKONOS, Quickbird) are used for ground-truthing the interpretations made from lower resolution imagery [[1](#_ENREF_1)], especially in countries where sample locations are hard to access. However, very high resolution imagery are rarely used for large areas due to the high financial and labour investment that is required [[2](#_ENREF_2)]. Moderate resolution data (30m resolution; e.g. Landsat) can be purchased, processed and managed at reasonable cost [[3](#_ENREF_3)]. In fact, historical Landsat data are available free from NASA [[4](#_ENREF_4)] but many images in the tropics are of limited use due to cloud coverage or seasonality [[5](#_ENREF_5)]. Coarse resolution data (250-1000m resolution; e.g. SPOT, MODIS) are also available free of charge. The daily temporal resolution provided by these satellites solves the problems of cloud cover and seasonality, but the resolution is too coarse for accurate carbon storage estimation [[6](#_ENREF_6)].

Present optical satellite sensors (e.g. Landsat, MODIS) cannot be used to estimate carbon stocks of tropical forests and woodlands with high certainty [[7](#_ENREF_7)]. Correlations have been developed between plot-based carbon estimates and vegetation indices (e.g. NDVI) [[8](#_ENREF_8), [9](#_ENREF_9)]. However, optical satellite sensors tend to saturate in high biomass regions [[7](#_ENREF_7), [10](#_ENREF_10), [11](#_ENREF_11)] and may be of limited availability due to cloud cover [[5](#_ENREF_5), [10](#_ENREF_10)]. Furthermore, the correlations developed are often regionally specific and so not transferable between studies or applicable across the globe [[11](#_ENREF_11)]. Very high-resolution images can be collected, typically from aeroplanes, and used to directly measure tree height and crown area. However, due to the high cost, it is often impractical to collect these data over vast areas, and so this technique is only particularly efficient for estimating biomass in small regions [[12](#_ENREF_12)].

Until recently, radar data have rarely been used for carbon mapping. However, the use of this technology is being explored. Radar is able to penetrate cloud cover and can collect data in day-time and night-time conditions. Early indications suggest that RADAR can be used to measure vegetation height and carbon storage estimated from this [[13](#_ENREF_13), [14](#_ENREF_14)], however, this technology is still in development and relatively costly [[3](#_ENREF_3)]. LiDAR sensors function on a similar concept to that of radar, measuring vegetation height and so estimating biomass [[15](#_ENREF_15), [16](#_ENREF_16)]. Recent studies [[17](#_ENREF_17), [18](#_ENREF_18)] have tended to use LiDAR data over microwave and radar techniques as they are less likely to saturate in high-biomass regions [[16](#_ENREF_16), [19](#_ENREF_19), [20](#_ENREF_20)]. However, due to the scattering of reflectance beams, these techniques have higher uncertainties for taller canopies and in montane regions, where terrain is more rugged [[19](#_ENREF_19), [21](#_ENREF_21)]. Despite this drawback, large-footprint LiDAR remote sensing far exceeds the capabilities of radar and optical sensors to estimate forest and woodland carbon stocks [[16](#_ENREF_16), [19](#_ENREF_19), [20](#_ENREF_20)]. However, currently aeroplane-mounted LiDAR instruments are too costly for use at large scales, and satellite based LiDAR systems are not yet widely available [[22](#_ENREF_22), [23](#_ENREF_23)]. In addition, techniques that use height as a proxy for AGB have high uncertainty in regions that obtain maximum height rapidly but continue to accumulate biomass for many years [[24](#_ENREF_24), [25](#_ENREF_25)].

Finally, GIS-based extrapolation of tree inventory plots using modelled statistical relationships with ancillary data (e.g. temperature data, precipitation data, topography) can be used to estimate carbon storage. Ancillary GIS data have three main advantages: 1) it is widely available and often free of charge; 2) it is often of moderate resolution (90m [[4](#_ENREF_4)]); and 3) correlations identified with these variables may provide indications of those that directly affect carbon storage. Developing an understanding of these influential variables is vital if accurate scenarios of future carbon storage are to be developed.

**SI2 – METHOD FOR OBTAINING CARBON VALUES FROM TREE INVENTORY PLOTS**

Using the quality-controlled dataset of 1,611 tree inventory plots (median 0.1ha, mean 0.1ha, mode 0.1ha [43 plots with multiple censuses; median 0.1ha, mean 0.5ha, mode 1.0ha]; see SI6 for a discussion on the limitations of the plot data) we calculated plot-level stand structure indices and aboveground carbon storage per unit area. We obtained the exponent and intercept of the population size-frequency distribution using the power law fit for each plot using the log-log transformation method. Whereby, for each plot, we created 10cm bin size-frequency distributions based on diameter at breast height (DBH), and a linear model of the logarithm of the frequency against the logarithm of the size class was fitted. Whilst not as accurate as the maximum likelihood estimation method, our simpler method is more stable for many of our plots, providing both the intercept and slope indicators of population structure, given that these variables need not be highly correlated [[26](#_ENREF_26)].

The quality controlled dataset contained 16,534 tree height measurements with concomitant diameter values. Trees with heights in excess of 80m (29 trees) were assumed to be erroneous and removed from the dataset because they were significant outliers within both this and previous data sets [[27](#_ENREF_27)]. Using these data we created DBH-height relationships using the equation forms shown in Table S9. In addition, we recognised that previous regional studies have identified that tree height varies significantly with altitude [[27](#_ENREF_27), [28](#_ENREF_28)]. Since mean annual temperature (MAT; obtained from the WorldClim data source [[29](#_ENREF_29)]) is a strong correlate of altitude, as well as dominating the primary axis of the principal components (PC) describing the environmental heterogeneity spanned by the plot network (see PC1 in Table S10), we also incorporated MAT into the equation forms as a linear fixed effect. Each plot was included as a random effect, accounting for the non-independence of errors and the best fit model was chosen using the *Akaike Information Criterion* (AIC).

We obtained wood specific gravity (WSG) data via the phylogenetic information provided by our tree inventory plots. We used a global wood density database, to extract species average WSG [[30](#_ENREF_30)]. This procedure provided over 32,000 trees with WSG data. When this was not possible the appropriate genus average (~14,000 trees), family average (~9,500 trees), plot average (~4,500 trees) and dataset average (~80 trees) were applied [[31](#_ENREF_31)]. Including WSG as an additional parameter in allometric equations reduces the biomass estimation error [[28](#_ENREF_28), [32](#_ENREF_32), [33](#_ENREF_33)]. Finally, carbon was assumed to be 50% of biomass [[34](#_ENREF_34)]. Hence, for all plots stand-level data was obtained on aboveground carbon storage, WSG, height, and population structure.In addition, we estimated plot biomass using moist forest tree allometry [[33](#_ENREF_33)] based on measurements of diameter at breast height (DBH) from our tree inventory plots, WSG (as described above) and height data (derived using the best fit DBH-height equation form [Equation 5.1; see SI4], if not measured in the tree inventory plots). Moist forest tree allometry was used in this study as, although all plots are classified as ‘dry’ when using precipitation categories [[33](#_ENREF_33)], the overwhelming majority are from the EAM and coastal forest (~92% of our collaborative dataset) and are considered as ‘moist forests’ by most authors [[27](#_ENREF_27), [35](#_ENREF_35)]. This discrepancy is perhaps because the east African precipitation follows a bimodal regime [[36](#_ENREF_36)] and thus is not well described using precipitation categories. The basal area and forest structure of the EAM and coastal forest area more similar to the moist forests used in the Chave et al (2005) dataset [[33](#_ENREF_33)] than to the dry forests [[28](#_ENREF_28)]. Additionally, EAM forest is more similar in species composition to moist Guineo-Congolian forests than to the dry forest miombo of east Africa, despite the close spatial proximity of the later [[27](#_ENREF_27)]. The dry forest data used to create the allometric equations in Chave et al (2005) include no data from Africa and thus may not be applicable to dry forest on this continent [[33](#_ENREF_33)], specifically the woodlands of our dataset (~5% of my collaborative dataset).

In order to investigate the effect of tree height on biomass estimates, allometric equations for AGB were applied that both include and exclude height data for each plot [[33](#_ENREF_33)]. Since the precipitation classification of the EAM forest is ambiguous, this procedure was applied to standard allometric equations for both tropical moist and tropical dry forest [[33](#_ENREF_33)]. Using both moist forest and dry forest allometric equations that include height, WSG and DBH [[33](#_ENREF_33)], the mean biomass for forested areas of our study area was 314.2 (300.6-327.6) Mg ha^-1^ and 280.2 (269.0-291.2) Mg ha^-1^ respectively (Table S11). Whilst both estimates are not vastly different, carbon estimated via the moist forest biomass equation was significantly greater than carbon estimated from the dry forest biomass equation (average difference = 34.0 [31.3-36.7] Mg ha^-1^) p-value <0.001). Excluding height from the allometric equations greatly exacerbates the difference between them, providing biomass estimates of 495.6 (475.8-515.2) Mg ha^-1^ and 262.4 (253.4-271.6) Mg ha^-1^ using the moist forest equation and dry forest equation respectively. This is because including height in the model significantly reduces the carbon estimate of the plots when utilising moist forest equations (average decrease = 181.4 [174.0-188.8] Mg ha^-1^, p-value < 0.001), but significantly increases carbon estimated for dry forest equations (average increase = 17.7 [14.5-20.8] Mg ha^-1^, p-value <0.001). If height is excluded from the allometric equations then the moist forest equation provides biomass estimates significantly higher than those produced by the dry forest equation (average decrease = 233.1 [222.1-244.0] Mg ha^-1^, p-value < 0.001). These preliminary findings support previous understanding that including stem height is more important than selecting the correct precipitation category when predicting plot biomass [[33](#_ENREF_33)], justifying our sole use of the moist forest equation, particularly considering the small sample size (none from Africa) used to develop the ‘dry forest’ equation.

For a smaller number of plots, multiple measurements were available over time (n = 43; mean plot size = 0.5 ha; mean measurement period = 3.9 years). We calculated changes in carbon storage rates arithmetically by dividing the difference in carbon storage estimates between censuses by the number of years separating them. Thus, obtaining plot-level data representing the aboveground carbon flux over time, a result of the net effect of growth, recruitment and mortality.

**SI3 – DATA COLLECTION & COLLATION**

**Data Collation**

Written memoranda of understanding, outlining the investigations to be undertaken and the data sharing procedure were constructed with local and international agencies working within the EAM. From this, a total of 2,462 tree inventory plots were obtained. The numerous data sources were created using a variety of methods from a host of organisations and individuals. These will now be described.

The majority of plots (2,302) were collated by Dr Antje Ahrends as part of the York Institute for Tropical Ecosystems (KITE) database. The KITE database is a large collaborative collection, predominantly made up for plots created by Frontier Tanzania (1,164), Dr Andrew Marshall (648), Prof Jon Lovett (375), and Dr Antje Ahrends (30). Frontier Tanzania created permanent sample plots of 50m by 20m every 450m along transects placed 900m apart [[37](#_ENREF_37)]. The diameter and species of every woody stem with a DBH over 10cm whose base fell within the designated plot area was recorded. For those stems whose base was bisected by the plot boundary, the data were recorded if more than half of the base lay within the plot. Height of the stem was recorded using a clinometer (whereby the angle to the top of the tree canopy was measured in accordance with Chave (2005) and the height calculated using trigonometry [[38](#_ENREF_38)]) for a random subsample of stems (approximately 10 from each of the following size classes: 10-20cm, 20-30cm, 30-40cm and >40cm) [[37](#_ENREF_37)]. These plots were measured by volunteers (mainly from the UK) supported by local botanists from the Tanzanian Forestry Research Institute (TAFORI) and experienced fieldwork coordinators. Dr Marshall and Dr Ahrends utilised the Frontier methodology when establishing a further 648 and 30 permanent sample plots respectively. The remainder of the plots were established by Prof Jon Lovett (375 plots) and Mr Roy Gereau (85 plots). Prof Lovett established 113 plots of 100m by 25m, recording the DBH, height and species of all woody stems over a 3cm DBH threshold [[39](#_ENREF_39)]. Of these stems, only those over 10cm DBH were included in the KITE dataset. The remainder of the plots established by Prof Lovett (262 plots), and those established by Mr Gereau were done using the 20-tree variable-area plotless technique [[40](#_ENREF_40)]. The nearest 20 trees of over 20cm DBH to an objectively chosen point were identified and DBH was recorded [[41](#_ENREF_41), [42](#_ENREF_42)]. Distance to the 21^st^ most distance tree was also recorded and half this distance can be considered to be the plot radius [[41](#_ENREF_41), [42](#_ENREF_42)]. However, this is a crude estimate and so we did not include these 347 plots in our analyses.

In addition to the KITE database, we were able to obtain data from six other sources, namely Prof Pantaleon Munishi (100 plots), Deo Shirima (4 plots), Mr Elmer Topp-Jorgenson (7 plots), Dr Gerry Hertel (33 plots) and Dr Jack Isango (16 plots). Those plots from Prof Munishi, Mr Topp-Jorgenson and Dr Isango were established at random locations but measured using the Frontier Tanzania protocol [[37](#_ENREF_37)]. The methodology of Dr Hertel and Mr Shirima differed from that of Frontier Tanzania only in that they used circular plots of 7.32m radius and square 100m by 100m plots respectively established at randomly chosen locations [[43](#_ENREF_43)].

Once the tree inventory data had been collated, a quality control and standardisation protocol was applied. This consists of two main steps: (1) Metadata quality control; and (2) Measurement bias detection.

Firstly, all plots lacking a recorded spatial location and a fixed area were discarded (770 plots). Plots where one or more diameter at breast height (DBH) data were known to be missing were also excluded (7 plots). Furthermore, plots smaller than 0.025ha (16 plots) were deemed to produce unreliable carbon estimates and so also removed from the dataset.

Secondly, to assess the potential impact of measurement bias, i.e. not measuring over buttresses and so overestimating biomass [[44](#_ENREF_44)], the remaining plots were grouped by the lead field researcher. Size frequency distributions, using 10cm size classes, were created for each of these groups. Forest size frequency distributions are suggested to conform to the -2 power law based on metabolic scaling [[45](#_ENREF_45)]. It has been argued that this rule is not globally applicable [[46](#_ENREF_46)], however, many studies accept this observation but highlight a tendency for the metabolic scaling model to over-predicted large stems [[47](#_ENREF_47)]. Additionally, whilst this law holds for large datasets, there is substantial variation at a plot level. This variation could be helpful in indicating potential biases in the data. For example, groups of plots showing a higher proportion of big trees than expected may indicate that the field team had a majestic forest bias. Hence, those researchers whose data significantly differed from this law, showing higher proportions of big trees, were discarded (1 researcher, 100 Plots).

**Data Collection**

The collaborative data described above was supplemented by the addition of 20 new 100m by 100m plots and 22 smaller plots (20m by 200m). The one hectare plots were established by Dr Marshall in the Udzungwa and Usamabara mountains to best capture the geographical range of the EAM. In 2007 and 2008, these plots were placed using randomised co-ordinates stratified by elevation in predominantly closed-canopy forest [[48](#_ENREF_48)]. Internationally accepted protocol was followed for the method of plot data collection [[49](#_ENREF_49)]. The DHB of stems ≥10cm were measured in 20 x 20m subplots. Smaller stems were not sampled as they typically only hold ~5% of biomass in mature African tropical forests [[34](#_ENREF_34), [50](#_ENREF_50)]. Stem heights were recorded using a clinometer or laser rangefinder across a range of size classes (10-19, 20-29, 30-39, 40-49, ≥50 cm DBH), with at least 10 randomly selected heights being recorded for each size class. A sub-sample of the measurements between the clinometer and laser range finder have been shown to be highly correlated (Pearson r^2^ = 0.977) [[48](#_ENREF_48)]. Trees were identified, with the aid of local botanists, following taxonomy of the Africa Plant Phylogeny Group [[51](#_ENREF_51)], with voucher specimens collected for verification at the Royal Botanic Gardens (Kew, London) if there was ambiguity.

In 2010, using the same methods, we recensused the one hectare plots, having previously established 22 smaller sample plots in 2009. The 22 smaller plots were established, using the same methods, in randomly chosen locations on the EAM, stratified by temperature and precipitation measures [[52](#_ENREF_52)]. We analysed the existing plot network and observed that the total dataset was relatively data poor at temperature and precipitation extremes. Specifically, we established more plots at locations experiencing mean annual temperatures of over 22°C but with mean annual precipitation levels of either below 1000mm (7 plots) or above 1600mm (7 plots). In addition, we established eight plots in forested areas with a mean annual temperature of less than 16°C. The plots we sampled were also subjected to the quality control and standardisation protocol described above. No plots were discarded, producing the final plot network which contained 1611 plots, with a mean plot size of 0.088 (median = 0.10, mode = 0.10) hectares.

For plots with multiple census data available, further quality control is possible. Building on standard measurement error detection protocols developed elsewhere [[34](#_ENREF_34), [53](#_ENREF_53)], it is possible to detect anomalies between remeasurements. Existing protocols treat as measurement error trees which appear to shrink more than 5mm in any measurement interval, or which are recorded as gaining in diameter faster than 40mm yr^-1^ [[34](#_ENREF_34), [53](#_ENREF_53)]. We selected all tree inventory plots with multiple censuses (60 plots and 9,090 trees in total). Most plots (41 out of 43) only had two censuses and so trees that were recruited or died between censuses were omitted, ensuring the growth rate of all trees remaining (8,475) could be calculated. With only two censuses, when an error is identified, it is difficult to know if the erroneous value is in the first or last census. We assumed that the original measurement was always the correct value. If the difference in final and initial DBH was less than -5mm then the final census DBH was replaced by the initial census DBH. Thus assuming that no growth occurred over this period and the ‘shrinking’ tree is due to error. This assumption was required for 314 trees (3.5% of all remeasured trees). Trees where the growth rate was over 40mm per year were also considered likely to be due to measurement error. To provide a realistic replacement estimate of growth rate, the average growth rate per year for the respective plot and size class (separated into 10-20, 20-40 and >40cm) was multiplied by the number of years between the censuses and this value was added to the initial census DBH giving a corrected final DBH. 43 trees (0.47% of all recensused trees) required this assumption.

**SI4 – LOCALLY DERIVED DBH-HEIGHT EQUATION**

The best fit DBH-height equation was the Gompertz, determined by choosing the fit with the lowest AIC value (p-value < 0.001; Equation 5.1; Table S9). There was a significant positive correlation between maximum canopy height and mean annual temperature (MAT) using the Gompertz (p-value < 0.001; Equation 5.2) and all other equation forms (Table S9).

**Equation 5.1**

$${\mathbf{Height =}\left( \mathbf{0.980726296 + 1.236525192 * MAT} \right)\mathbf{* e}}^{\mathbf{(-(-0.974598751 + 0.126698008 * MAT) * e}^{\mathbf{(-(0.068341379 - 0.001264387 * MAT) * DBH))}}}$$

**Equation 5.2**

$$\mathbf{Maximum height = 0.980726296 + 1.236525192 * MAT}$$

Within our plot data, height-MAT relationships differ amongst tree size classes (Figure S6). At lower mean annual temperatures the smallest size classes reach a peak in height, with height decreasing at higher temperatures. Larger size classes peak in height at higher temperatures, with trees >40 cm apparently reaching their height maxima at higher air temperatures than found today. Specifically, stems with a 10cm DBH are estimated to obtain maximum height of 11.5 m (95% CI: 8.3-14.3) in temperatures of 12.0 °C (9.8-16.2), while stems of 40cm DBH may not reach their maximum of 19.7 m (9.7-41.3) until temperatures of 22.1 °C (18.5-38.0). Size classes between 10 and 40cm DBH show intermediate maxima (Figure S6; Table S12). This implies that, initially, stem height increases with temperature (or variables correlated with temperature, although we find that windspeed, soil fertility and soil water availability are poorly correlated with temperature [Table S10]). This result is expected under the cohesion-tension theory, whereby negative pressure gradients and surface tension provide the forces necessary to lift water against gravity [[54](#_ENREF_54)], provided that water is not limiting [[55](#_ENREF_55)]. However, nutrient and water limitation could indirectly be driving the maxima across all DBH ranges, with small stems being outcompeted by larger stems and therefore reaching maxima at lower temperatures [[56-59](#_ENREF_56)] (Figure S6; Table S12).

**SI5 – DISCUSSION OF CLIMATIC AND EDAPHIC CORRELATIONS**

After anthropogenic effects, climatic variables are the next most influential correlate of carbon storage. The effect of climate on tropical forest biomass is quite well documented but also highly contentious [[60-62](#_ENREF_60)]. Our results clearly demonstrate that the temperature range (the difference between mean monthly maximum and minimum temperatures), and not the mean annual temperature, is key to understanding carbon storage in the tropical forests of the EAM. However, our results appear to conflict with expectations from theory [[60](#_ENREF_60)]. Respiration is known to be correlated with high night-time temperatures [[63](#_ENREF_63)], while high day-time temperatures may result from high insolation, leading to increased photosynthesis, provided that water is not limiting [[64](#_ENREF_64)]. However, our findings indicate that carbon storage actually decreases as temperature range widens, i.e. with higher monthly maxima and lower monthly minima temperatures. As the temperature range increase, both the potential stem density (indicated by the intercept of the power law relationship) and WSG increase and so the reduction of carbon storage is driven by the decreasing proportion of larger stems. A possible explanation for these results can be found in niche theory, with each species having a unique ‘goldilocks zone’ in which it functions most efficiently [[65](#_ENREF_65)]. Typically, large-stemmed species are specialists, growing slowly in a specific niche over a long period of time [[66](#_ENREF_66), [67](#_ENREF_67)]. Thus, if environments are more constant (with a lower temperature range) then, under niche theory, each locality will be occupied by species specifically adapted to function best at that temperature, thus resulting in many large stems and high biomass [[68](#_ENREF_68), [69](#_ENREF_69)]. Areas experiencing high temperature variation may be occupied with generalist species, having to tolerate a variety of temperatures, and resulting in lower overall productivity and biomass. In addition, extreme climate variations are known to increase mortality [[53](#_ENREF_53)] increasing dynamism, reducing the residence time of carbon and potentially killing large stemmed species before they grow to their full capacity, preventing the accumulation of high biomass levels.

Precipitation is also known to be an important variable influencing carbon storage [[70](#_ENREF_70)]. Our best fit model suggests that increased dry season length reduces carbon storage, whereas drought intensity does not have a significant affect. In times of water scarcity, plants close stomata to reduce water loss through transpiration, leading to a reduction in carbon assimilation [[71](#_ENREF_71)]. Interestingly, precipitation-based variables were not found to significantly correlate with any of the components of carbon storage and so the mechanism driving this correlation is unclear. Previously studies investigating the derivatives of carbon storage have produced conflicting results [[72-74](#_ENREF_72)].

Within the next century, the region is predicted to become both warmer and wetter, having a similar length dry season but experiencing increased seasonality, with higher probabilities of intense drought and flooding [[75](#_ENREF_75), [76](#_ENREF_76)]. Thus, our results support the anticipated ‘greening’ expected as a result of the general trend shown in future climate scenarios (i.e. high temperatures and levels of precipitation may lead to increased carbon storage) [[75](#_ENREF_75)]. However, caution should be applied as more intense droughts and/or floods may hinder growth. Specifically, the water limitation experienced in times of drought may complicate the predicted increase in growth as a result of the increasing temperature, despite the mediating action of increasing CO_2_ concentrations on plant water use efficiency.

Soil water availability is also known to effect plant growth and carbon storage [[77](#_ENREF_77)]. However, this effect can be complex, with both too little water (droughts) and too much water (floods) known to reduce carbon storage [[53](#_ENREF_53), [78](#_ENREF_78), [79](#_ENREF_79)]. We find carbon storage decreases with an increase in soil water availability, driven by a reduction in WSG and the proportion of large stems, although somewhat buffered by an increasing density of smaller stems. Our result may be considered counter-intuitive, with water scarcity known to lead to a reduction in carbon assimilation [[71](#_ENREF_71)]. However, droughtedness has already been accounted for in our model and thus, the observed effect of soil water availability may be structural rather than hydrological. More saturated soils, may be unable to provide large stems with enough structural support to remain upright, particularly in montane areas (such as the EAM) where slopes may be extremely steep. Thus, larger stems may not be present in saturated soils, leading to low levels of carbon storage. In addition, drier, sandier soils appear to filter species towards those with higher WSG [[53](#_ENREF_53), [62](#_ENREF_62)].

We find no effect of soil fertility on tropical forest biomass. Previous studies have shown that more fertile soils have the potential to support higher levels of growth, but that these are often also more dynamic and so likely to have higher mortality [[80](#_ENREF_80)]. However, regional studies have produced conflicting results, finding positive, negative and no correlations between soil fertility and AGB [[81-85](#_ENREF_81)]. The most recent, in-depth studies by Quesada *et al* (2009, 2012) support our result [[84](#_ENREF_84), [85](#_ENREF_85)]. They found that Amazon forest biomass was not significantly correlated with soil conditions once corrections for spatial autocorrelation were applied, perhaps because aboveground biomass does not seem to be directly influenced by edaphic conditions unless conditions are particularly extreme [[84](#_ENREF_84), [85](#_ENREF_85)]. The debate surrounding the effect of soil properties on the components of carbon storage is as equally contentious to that surrounding AGB. For example, in the Amazon, WSG has been found to have negative correlations with soil fertility [[84-86](#_ENREF_84)], but, similar to results presented here, no correlations have also been reported [[74](#_ENREF_74), [87](#_ENREF_87)]. In general, edaphic characteristics in the tropics are relatively understudied and involve large uncertainties, perhaps hindering our understanding of any mechanisms involved [[84](#_ENREF_84), [88](#_ENREF_88), [89](#_ENREF_89)]. The lack of accurate, high resolution soil data was a key limitation of our study, and many other studies (see SI6). This emphasises the need for tropical forest research and REDD + projects, both regional and global, to include soil in their investigations.

Although additional variables, such as solar radiation and fire, were not found to affect carbon storage estimates, we demonstrate significant correlations with its components. Forests experiencing lower light levels show a lower potential stem density, but a higher proportion of larger trees. Larger trees are usually taller [[24](#_ENREF_24)] and so would dominate in regions receiving less solar radiation, intercepting the little light available and decreasing the number of smaller stems present in the understory [[56](#_ENREF_56)]. The reduced number of small stems in forests experiencing low light levels may be countered by the increased proportion of large stems, leaving overall carbon storage values unaffected. Fire, on the other hand, is negatively correlated with the proportion of big trees, but this affect may be countered by an increase in WSG, again resulting in no overall effect on carbon storage. Stems of high WSG are able to provide equal strength to lower WSG stems, at a reduced DBH. Thus, high WSG stems show a reduced surface area and lower costs of bark construction and maintenance. These costs are particularly important in fire-prone habitats, where thick bark is needed for protection [[90](#_ENREF_90)]. Hence, smaller, high WSG stems are increasingly selected for as the probability of fire occurrence increases.

Thus, the variables correlating with aboveground carbon storage and its components are numerous (spanning anthropogenic, climatic and edaphic variables) and complex. But, how do the components interact to contribute to carbon storage? We find that all components correlate with carbon storage, although WSG and the proportion of large stems dominate. In addition, we find that there are complex interactions between all components. For example, the proportion of large stems and the potential stem density do not combine additively with maximum canopy height to contribute to aboveground carbon storage. In areas of low potential stem density and areas with a low proportion of large stems, carbon storage is positively correlated with maximum canopy height. However, this correlation is reversed in areas of high potential stem density and also areas with a high proportion of large stems. This change in correlation may be due to maximum canopy heights not being attained in areas of high potential stem density or areas with a high proportion of large stems. Up to 25% of species examined in Bolivian forest fail to show asymptotic DBH-height relationships [[59](#_ENREF_59)]. Furthermore, the maximum height may not be realised as mechanical damage and/or death can prevent this [[25](#_ENREF_25), [91](#_ENREF_91)]. Thus, competition amongst stems in areas of high stem density and areas with a high proportion of large stems may prevent stems reaching the predicted maximum canopy height, and so altering the positive correlation between maximum canopy height and carbon storage that may be expected.

In Amazonia, WSG has been proposed to drive landscape-scale variations in aboveground biomass [[31](#_ENREF_31)]. In our study, while highly influential, WSG does not combine additively with other components to impact on carbon storage. In areas of low WSG, as expected, the potential stem density (intercept of the size-frequency power law relationship) and the proportion of large stems (gradient of the same relationship) correlate positively with carbon storage. However, the low WSG provides less structural support for a given diameter than in higher WSG areas [[90](#_ENREF_90)], this may result in stems not obtaining maximum canopy height. Indeed, we find stem height to be disproportionately below maximum canopy height in low WSG areas (p-value < 0.01). In high WSG areas, the dense wood provides stems with more structural support, allowing them to attain maximum canopy height. Thus, we observe the expected positive correlation between carbon storage and maximum canopy height, which dominates variation in carbon storage in these areas, decoupling the previous size-frequency component effects.

**SI6 – STUDY LIMITATIONS**

Despite stringent quality control and standardisation protocols, there are limitations to our dataset. The mean plot size used in this study is small for a tropical tree-dominated vegetation study, at 0.09ha. Biomass estimates resulting from small plots are known to suffer from a left-hand skew, leading to high uncertainties [[92](#_ENREF_92)]. However, as the number of plots increases, the confidence also increases [[92](#_ENREF_92)]. Thus, results obtained from our extensive network of small plots are likely to be robust, covering a sampled area of >160 ha, although caution is still recommended. Secondly, the plots have been measured in different regions by different field teams and using different plot designs. This could be a further source of error if methods were not fully comparable; however, all plots from field teams whose data showed measurement bias were removed. Thirdly, height was not recorded for every stem, only ~34% of sampled stems had height measurements. For stems lacking height data, a value was derived from the DBH using the best fit DBH-height equation available for the region (Equation 5.1). Finally, our biomass estimates utilise pantropical allometric equations [[33](#_ENREF_33)]. However, no data used to derive these relationships was from Africa or from montane environments [[33](#_ENREF_33)]. By utilising the combination of DBH, height and wood specific gravity data, we have minimised this source of error as much as possible [[32](#_ENREF_32), [33](#_ENREF_33)]. However, these errors may mean that the data used to calculate the correlation models used in this investigation may not be a true representation of carbon storage, and its components, on-the-ground. Ideally, an extensive plot network, developed using global standard protocols containing multiple censuses over time would be available. However, such a network has not yet been developed across the EAM.

In all our models there is a large amount of unexplained variation. The R-squared values for our correlation models vary between 0.18 and 0.41. Hence, at least 60% of the variation in carbon storage and its components are unexplained by our correlation models. This is likely to be due to three main reasons. Firstly, although we used the highest resolution datasets that are freely available, several of the associated variables are of relatively poor resolution or are very sparsely located across the EAM (including; wind, light and soil variables [Table S6]). This is particularly important here as our plot network comprised of many small plots (median, mean and mode are all 0.1ha). Small plots contain a higher level of variation than larger plots, and this is likely to be unexplained in statistical models if datasets describing heterogeneity are not available on the same scale. Secondly, forest characteristics in the present are the result of growth, recruitment and mortality over many years. It is difficult to obtain data on historical variables and yet these could have had a significant impact on present day carbon storage and other forest characteristics. We included the extent of historical logging and this was retained as an important variable in 75% of the final models, being the most influential correlate of carbon storage (Tables 3-4; Tables S1-S3). Thirdly, present day information is also lacking, for example datasets describing physical soil properties in the study area are unavailable. The lack of data (albeit completely lacking or at courser-scale resolution) may mean that the correlations identified from the correlation equations produced here are inappropriate. Furthermore, the unexplained variation resulting from these data inadequacies is problematic when investigating how the components of carbon storage combine to produce observed carbon storage. As such, these results should be regarded as a first order estimate. In the future, higher resolution and historical datasets may enable further correlations to be observed when producing models estimating carbon storage, as well as each of the component variables. By reducing the level of unexplained variation in these models, more accurate assessments of how the components of carbon storage interact could be made.

The limited number of multiple censuses available (n=43 plots with >1 census) within our study area gives rise to uncertainty in our estimated sequestration rates. Calculating carbon sequestration requires multiple census tree inventory data, which are rare across the EAM. We have collated the most extensive network of recensused tree inventory plots within my study area to date. However, during the time period covered by my censuses, climatic conditions tended to be drier than over recent decades [[93](#_ENREF_93)]. As such, mortality during this period may have been higher than usual background rates. By contrast, sampling done over shorter time periods may result in overestimation of rates of carbon sequestration as rare stochastic mortality events may not be sampled [[94](#_ENREF_94), [95](#_ENREF_95)]. However, there is debate surrounding the importance of these rare disturbance events [[96](#_ENREF_96)]. During the sampling period, mortality events were recorded (for example, by both windstorms and felling) but 79% of my plots had a census history of <5 years, with only one plot exceeding 10 years, and so our estimates of carbon sequestration rates may be inflated, indicating that the study area maybe a larger carbon source than presented here. Whilst we examined numerous candidate variables (Table S6), due to our limited dataset, we were only able to examine PC axes (Table S4). Numerous potential influential variables of changing carbon storage have been identified in tropical tree communities [[97](#_ENREF_97), [98](#_ENREF_98)]. Further work is needed to expand the existing multiple census inventory plot networks [[34](#_ENREF_34), [53](#_ENREF_53)] in order to shed further light on the relative importance of these influential variables. The production of datasets able to separate the multiple variables that correlate with changes in carbon storage would lead to an increased ability to anticipate any future changes, perhaps resulting from population increases, climate change and/or changes in nutrient deposition.

**SI7 - DEFINITIONS**

**5.3.1 Population Pressure**

Natural resources are subject to pressure from both local populations and distant demand centres, such as cities. We use population variables as an attempt to represent the pressure exerted on a particular point in space by all persons across the landscape. Thus, we define population pressure as the pressure on forest and woodland resources, resulting in degradation, when all persons in the landscape (not just those living locally) have been accounted for. We assume that the pressure on a location *i* increases linearly according to the number of persons (*p*) in a remote location (*j*). We also assume that the weight (*w*) given to a remote population decreases exponentially with distance (*d*). Hence, population pressure can be represented mathematically as:

$${pressure}_{i}= \sum_{j=1}^{N} p_{j}{.w}_{ij} \mathrm{where} w_{ij}=exp(-\left( \frac{d_{ij}}{\sigma} \right)^{2})$$

and *N* is the number of locations of interest [[99](#_ENREF_99)].

These variables were calculated using a 1km^2^ population density grid based on [LandScan [100](#_ENREF_100)], correcting for ward-level census counts and protected area data [[101](#_ENREF_101)]. To aid computational efficiency, the 1km^2^ population grid was resampled to a 25km^2^ resolution, meaning ‘local’ populations are defined as those within the same 25km^2^ grid cell as the forest and/or woodland. Population pressure was calculated at this coarser scale using a range of plausible sigma values (σ = 5, 15, 25, 50) to allow a variety of spatial scales of distant pressure, before being bilinearly interpolated back to a 1km^2^ resolution [[99](#_ENREF_99)]. The natural logarithm of the population pressure grid was used for linear regressions as it better conformed to a normal distribution.

**5.3.2 Soil fertility**

Some studies have suggested aboveground carbon storage is correlated with soil nutrient availability, reporting both positive [[62](#_ENREF_62), [80](#_ENREF_80), [102](#_ENREF_102)] and negative [[83](#_ENREF_83), [84](#_ENREF_84)] correlations with soil fertility (see Section 2.3.2). We seek to determine the whether soil fertility is an influential correlate of aboveground carbon storage in eastern Tanzanian forests and woodlands. The spatial variation of edaphic variables is poorly understood in this region due to data deficiencies (further discussed in SI6). However, it is possible to use existing data from the SOTER database [[103](#_ENREF_103)] to provide a first order estimate of edaphic variation. Whilst the SOTER database provides useful estimates of soil nitrogen and carbon content, as an indication of overall soil fertility, only effective cation exchange capacity (eCEC*)* is provided [[104](#_ENREF_104)]. eCEC is a crude measure of soil fertility because it would show higher values in areas high in potassium and phosphorus, nutrients positively correlated with growth [[80](#_ENREF_80), [84](#_ENREF_84), [105](#_ENREF_105)], but also in areas of high aluminium content, which is toxic to many plants [[106](#_ENREF_106)]. We calculate soil fertility as:

$$\left( \frac{\left( 100-A \right)}{100} \right)*eCEC$$

where *A* is the aluminium saturation.

This partially negates the effect of aluminium levels in the overall measure of soil fertility, so that high values should be indicative of high potential growth rates. Thus, we define soil fertility as the eCEC of the soil, once the presence of aluminium ions has been controlled for.

**TABLES**

**Table S1** The coefficients and associated p-values of the variables correlated with WSG using both forward and backward selection procedures.

| **Variable** (where appropriate, units are given in brackets) | **Group** | **Forward** | | **Backward** | |
| --- | --- | --- | --- | --- | --- |
|  |  | **Coefficient** | **p-value** | **Coefficient** | **p-value** |
| **(Intercept)** | n/a | -1.98E+02 | 2.14E-05 | -1.59E+02 | 6.20E-04 |
| **Natural logarithm of the population pressure with decay constant of 16.7km** | Anthropogenic | -7.33E-03 | 9.78E-02 | n/a | n/a |
| **Natural logarithm of the population pressure with decay constant of 12.5km** | Anthropogenic | n/a | n/a | -1.33E-02 | 5.20E-03 |
| **Natural logarithm of the cost distance to roads** | Anthropogenic | 1.89E-02 | 9.40E-11 | n/a | n/a |
| **Distance to roads** (km) | Anthropogenic | n/a | n/a | 2.69E-06 | 6.13E-05 |
| **Natural logarithm of the cost distance to Dar es Salaam** | Anthropogenic | -4.91E-02 | 9.90E-07 | n/a | n/a |
| **Cost distance to Dar es Salaam** | Anthropogenic | n/a | n/a | -1.50E-06 | 2.00E-16 |
| **Natural logarithm of the cost distance to market towns** | Anthropogenic | n/a | n/a | 2.24E-02 | 9.99E-07 |
| **Governance - local** (national/local/joint/unknown) | Anthropogenic | 3.83E-03 | 8.71E-01 | n/a | n/a |
| **Governance - national** (national/local/joint/unknown) | Anthropogenic | -7.71E-03 | 6.46E-02 | n/a | n/a |
| **Governance - unknown** (national/local/joint/unknown) | Anthropogenic | 3.93E-02 | 1.17E-01 | n/a | n/a |
| **Mean annual monthly temperature range** (°C) | Climatic | 2.90E-02 | 2.00E-16 | n/a | n/a |
| **Mean annual maximum monthly temperature** (°C) | Climatic | n/a | n/a | 2.62E-02 | 2.00E-16 |
| **Mean annual minimum monthly temperature** (°C) | Climatic | n/a | n/a | -2.53E-02 | 2.00E-16 |
| **Wind speed** (m s^-1^) | Climatic | -3.70E-05 | 2.04E-02 | -4.98E-05 | 7.84E-04 |
| **Mean number of dry months annually** | Climatic | n/a | n/a | 3.71E-03 | 2.51E-02 |
| **pH of the soil** | Edaphic | 9.68E-02 | 2.00E-16 | 8.63E-02 | 1.27E-12 |
| **Total available water capacity of the soil** (vol. %, -33 to -1500kPA conforming to USDA standards) | Edaphic | -1.22E-02 | 3.90E-09 | -7.01E-03 | 4.59E-02 |
| **Total nitrogen content of the soil** (g kg^-1^) | Edaphic | n/a | n/a | 7.14E-03 | 1.31E-02 |
| **Total carbon content of the soil** (g kg^-1^) | Edaphic | n/a | n/a | 5.59E-02 | 8.35E-02 |
| **Percentage sand content of the soil** (%) | Edaphic | n/a | n/a | 3.99E-03 | 2.02E-03 |
| **Annual mean burned area probability** | Fire | 2.63E+01 | 3.80E-06 | 2.09E+01 | 2.62E-04 |
| **Mean annual global horizontal solar radiation** (kW m^-2^ day^-1^) | Geographic | 7.47E-05 | 3.40E-04 | 7.93E-05 | 1.32E-04 |
| **Spatial autocorrelation term 4** | Spatial | -4.88E+00 | 3.20E-04 | -1.16E+01 | 1.28E-03 |
| **Spatial autocorrelation term 6** | Spatial | 1.04E-01 | 5.83E-08 | 8.83E-02 | 2.92E-06 |
| **Spatial autocorrelation term 8** | Spatial | 1.27E-01 | 1.34E-04 | n/a | n/a |
| **Spatial autocorrelation term 5** | Spatial | 9.83E+00 | 1.61E-05 | n/a | n/a |
| **Spatial autocorrelation term 2** | Spatial | -1.18E-01 | 1.46E-05 | 7.70E+00 | 5.15E-04 |
| **Spatial autocorrelation term 1** | Spatial | n/a | n/a | -7.79E+00 | 5.14E-04 |
| **Spatial autocorrelation term 3** | Spatial | n/a | n/a | 7.89E+00 | 5.27E-04 |

**Table S2** The coefficients and associated p-values of the variables correlated with the intercept of the power law relationship using both forward and backward selection procedures.

| **Variable** (where appropriate, units are given in brackets) | **Group** | **Forward** | | **Backward** | | | |
| --- | --- | --- | --- | --- | --- | --- | --- |
|  |  | **Coefficient** | **p-value** | **Coefficient** | **p-value** | |  |
| **(Intercept)** | n/a | -2.95E+01 | 1.89E-11 | -5.37E+02 | 9.92E-08 |  |  |
| **Natural logarithm of the cost distance to roads** | Anthropogenic | -5.29E-01 | 9.09E-10 | -3.09E-01 | 1.05E-04 |  |  |
| **Historical logging – Partially logged** (no logging/partially logged) | Anthropogenic | 1.06E+00 | 1.68E-05 | 1.67E+00 | 2.18E-09 |  |  |
| **Natural logarithm of the population pressure with decay constant of 12.5km** | Anthropogenic | 8.45E-01 | 1.23E-12 | 4.98E-01 | 1.06E-05 |  |  |
| **Cost distance to Dar es Salaam** | Anthropogenic | 1.40E-05 | 3.47E-06 | n/a | n/a |  |  |
| **Mean annual monthly temperature range** (°C) | Climatic | 8.46E-01 | 2.00E-16 | 9.52E-01 | 2.00E-16 |  |  |
| **Total available water capacity of the soil** (g kg^-1^) | Edaphic | 2.72E-01 | 3.82E-10 | 2.47E-01 | 1.22E-07 |  |  |
| **Mean burned area probability in the fourth quarter** | Fire | n/a | n/a | 2.05E+02 | 1.27E-03 |  |  |
| **Mean annual global horizontal solar radiation** (kW m^-2^ day^-1^) | Geographic | 3.64E-03 | 9.90E-08 | 3.76E-03 | 4.37E-07 |  |  |
| **Spatial autocorrelation term 1** | Spatial | n/a | n/a | -3.35E+01 | 4.45E-07 |  |  |
| **Spatial autocorrelation term 2** | Spatial | n/a | n/a | 3.30E+01 | 4.45E-07 |  |  |
| **Spatial autocorrelation term 3** | Spatial | n/a | n/a | 3.29E+01 | 4.48E-07 |  |  |
| **Spatial autocorrelation term 6** | Spatial | n/a | n/a | 1.24E+00 | 1.93E-06 |  |  |

**Table S3** The coefficients and associated p-values of the variables correlated with the gradient of the power law relationship using both forward and backward selection procedures.

| **Variable** (where appropriate, units are given in brackets) | **Group** | **Forward** | | **Backward** | |
| --- | --- | --- | --- | --- | --- |
|  |  | **Coefficient** | **p-value** | **Coefficient** | **p-value** |
| **(Intercept)** | n/a | 7.74E+00 | 1.05E-10 | 9.21E+01 | 1.53E-04 |
| **Natural logarithm of the cost distance to roads** | Anthropogenic | 1.22E-01 | 1.12E-08 | 6.05E-02 | 1.15E-03 |
| **Historical logging** (no logging/partially logged) | Anthropogenic | -1.01E-01 | 9.71E-02 | -2.95E-01 | 8.78E-06 |
| **Natural logarithm of the population pressure with decay constant of 20.8km** | Anthropogenic | -2.50E-01 | 6.99E-10 | -1.75E-01 | 2.91E-05 |
| **Cost distance to Dar es Salaam** | Anthropogenic | -3.76E-06 | 1.62E-06 | n/a | n/a |
| **Mean annual monthly temperature range** (°C) | Climatic | -2.05E-01 | 2.00E-16 | -2.38E-01 | 2.00E-16 |
| **Total available water capacity of the soil** (g kg^-1^) | Edaphic | -5.40E-02 | 1.24E-07 | -4.16E-02 | 2.10E-04 |
| **Mean burned area probability in the fourth quarter** | Fire | -5.81E+01 | 1.23E-04 | -5.62E+01 | 2.51E-04 |
| **Mean annual global horizontal solar radiation** (kW m^-2^ day^-1^) | Geographic | -8.68E-04 | 4.20E-07 | -1.05E-03 | 2.47E-08 |
| **Spatial autocorrelation term 1** | Spatial | n/a | n/a | 5.42E+00 | 7.01E-04 |
| **Spatial autocorrelation term 2** | Spatial | n/a | n/a | -5.35E+00 | 6.99E-04 |
| **Spatial autocorrelation term 3** | Spatial | n/a | n/a | -5.32E+00 | 7.03E-04 |
| **Spatial autocorrelation term 6** | Spatial | n/a | n/a | -2.08E-01 | 9.66E-04 |

**Table S4** The PC axes derived from the candidate variables (Table S6). Axes shown in this study to significantly affect carbon sequestration are indicated by an asterisk.

| **Variable** | **PC1 Coefficient*** | **PC2 Coefficient** | **PC3 Coefficient*** | **PC4 Coefficient** | **PC5 Coefficient*** |
| --- | --- | --- | --- | --- | --- |
| **Population pressure with decay constant of 41.6km** | 0.18 | -0.08 | -0.03 | -0.12 | 0 |
| **Population pressure with decay constant of 20.8km** | 0.19 | -0.02 | 0.02 | -0.04 | 0.01 |
| **Population pressure with decay constant of 16.7km** | 0.19 | -0.02 | 0.02 | -0.02 | 0.01 |
| **Population pressure with decay constant of 12.5km** | 0.19 | -0.01 | 0.02 | -0.01 | -0.01 |
| **Population pressure with decay constant of 8.6km** | 0.19 | -0.02 | 0 | -0.01 | -0.05 |
| **Population pressure with decay constant of 4.2km** | 0.18 | -0.06 | -0.03 | -0.05 | -0.09 |
| **Population pressure with decay constant of 1.7km** | 0.17 | -0.08 | -0.07 | -0.08 | -0.07 |
| **Natural logarithm of the population pressure with decay constant of 41.6km** | 0.18 | -0.06 | -0.01 | -0.16 | -0.03 |
| **Natural logarithm of the population pressure with decay constant of 20.8km** | 0.18 | 0.08 | 0.08 | -0.04 | -0.02 |
| **Natural logarithm of the population pressure with decay constant of 16.7km** | 0.18 | 0.09 | 0.09 | -0.02 | -0.03 |
| **Natural logarithm of the population pressure with decay constant of 12.5km** | 0.18 | 0.11 | 0.09 | -0.01 | -0.05 |
| **Natural logarithm of the population pressure with decay constant of 8.6km** | 0.18 | 0.1 | 0.07 | 0 | -0.08 |
| **Natural logarithm of the population pressure with decay constant of 4.2km** | 0.18 | 0.05 | -0.02 | 0.01 | -0.17 |
| **Natural logarithm of the population pressure with decay constant of 1.7km** | 0.12 | -0.04 | -0.22 | -0.05 | -0.26 |
| **Cost distance to Dar es Salaam** | -0.17 | 0.01 | 0.22 | -0.08 | -0.01 |
| **Cost distance to market towns** | -0.12 | -0.14 | 0.3 | 0.06 | 0.04 |
| **Distance to roads** | -0.1 | -0.22 | 0.09 | -0.08 | -0.24 |
| **Distance to Dar es Salaam** | -0.16 | 0.04 | -0.03 | -0.32 | -0.1 |
| **Distance to market towns** | -0.13 | -0.21 | -0.09 | 0.02 | 0 |
| **Natural logarithm of the cost distance to Dar es Salaam** | -0.17 | 0.02 | 0.19 | -0.05 | -0.03 |
| **Natural logarithm of the cost distance to market towns** | -0.11 | -0.15 | 0.3 | 0.08 | -0.02 |
| **Natural logarithm of the cost distance to roads** | -0.09 | -0.23 | 0.15 | -0.04 | -0.22 |
| **Mean annual temperature** | -0.08 | 0.17 | -0.32 | 0.1 | -0.17 |
| **Mean annual maximum monthly temperature** | -0.1 | 0.14 | -0.3 | 0.06 | -0.15 |
| **Mean annual minimum monthly temperature** | -0.06 | 0.19 | -0.31 | 0.12 | -0.18 |
| **Mean annual monthly temperature range** | -0.07 | -0.22 | 0.14 | -0.19 | 0.19 |
| **Mean maximum cumulative water deficit** | -0.1 | -0.15 | -0.21 | -0.14 | 0.06 |
| **Mean number of dry months annually** | -0.03 | -0.25 | -0.18 | -0.05 | 0.03 |
| **Wind speed** | 0.16 | -0.14 | -0.04 | 0.12 | 0.11 |
| **Total nitrogen content of the soil** | -0.02 | -0.17 | -0.03 | -0.11 | -0.53 |
| **Total carbon content of the soil** | 0.01 | -0.23 | 0.15 | 0.31 | -0.13 |
| **Percentage sand content of the soil** | -0.06 | 0.17 | -0.12 | -0.23 | 0.43 |
| **Total available water capacity of the soil** | 0.01 | 0.18 | 0.21 | 0.41 | 0.03 |
| **pH of the soil** | 0 | 0.23 | 0.17 | 0.28 | -0.19 |
| **Soil fertility** | 0 | -0.29 | -0.04 | -0.05 | 0.02 |
| **Mean burned area probability in the fourth quarter** | -0.12 | -0.2 | -0.15 | 0.17 | 0.07 |
| **Mean burned area probability in the third quarter** | -0.12 | -0.2 | -0.15 | 0.17 | 0.07 |
| **Annual mean burned area probability** | -0.12 | -0.2 | -0.15 | 0.17 | 0.07 |
| **Aspect** | 0.03 | 0.14 | 0.06 | -0.24 | 0.1 |
| **Mean annual global horizontal solar radiation** | -0.11 | 0 | -0.17 | 0.31 | 0.17 |
| **Spatial autocorrelation term 1** | 0.18 | -0.08 | -0.03 | 0.08 | 0.08 |
| **Spatial autocorrelation term 2** | 0.19 | -0.04 | -0.01 | 0.13 | 0.08 |
| **Spatial autocorrelation term 3** | 0.17 | -0.14 | -0.05 | 0.02 | 0.06 |
| **Spatial autocorrelation term 4** | 0.17 | -0.13 | -0.05 | 0.04 | 0.07 |
| **Spatial autocorrelation term 5** | 0.18 | -0.06 | -0.01 | 0.12 | 0.08 |
| **Spatial autocorrelation term 6** | -0.17 | 0.14 | 0.04 | -0.07 | -0.07 |
| **Spatial autocorrelation term 7** | 0.18 | -0.06 | -0.01 | 0.12 | 0.08 |
| **Spatial autocorrelation term 8** | 0.17 | -0.14 | -0.05 | 0.02 | 0.06 |

**Table S5** The coefficients and associated p-values of the correlations between the derivatives of carbon storage (the intercept of the power law relationship, the gradient of the power law relationship, WSG and maximum canopy height [shown here are intercept, gradient, WSG and height respectively]) and the carbon storage estimates made in this study.

| **Variable** | **4^th^ order interactions** | | **2^nd^ order interactions** | |
| --- | --- | --- | --- | --- |
|  | **Coefficient** | **p-value** | **Coefficient** | **p-value** |
| **(Intercept)** | 2.77E+03 | 2.00E-16 | 5.08E+02 | 2.00E-16 |
| **height** | -8.61E+01 | 2.00E-16 | -2.71E+00 | 3.99E-07 |
| **intercept** | 2.79E+02 | 2.00E-16 | 1.93E+02 | 2.00E-16 |
| **gradient** | 3.97E+03 | 2.00E-16 | 1.04E+03 | 2.00E-16 |
| **WSG** | -4.25E+03 | 2.00E-16 | -5.95E+02 | 2.00E-16 |
| **height:intercept** | -9.12E+00 | 5.61E-12 | -2.10E+00 | 2.00E-16 |
| **height:gradient** | -1.32E+02 | 2.00E-16 | -7.53E+00 | 2.00E-16 |
| **height:WSG** | 1.42E+02 | 2.00E-16 | 8.23E+00 | 2.00E-16 |
| **intercept:gradient** | -1.64E+02 | 2.00E-16 | -4.33E+00 | 2.00E-16 |
| **intercept:WSG** | -3.59E+02 | 6.84E-12 | -2.27E+02 | 2.00E-16 |
| **gradient:WSG** | -5.87E+03 | 2.00E-16 | -1.20E+03 | 2.00E-16 |
| **height:intercept:gradient** | 5.90E+00 | 2.00E-16 | n/a | n/a |
| **height:intercept:WSG** | 1.09E+01 | 5.36E-07 | n/a | n/a |
| **height:gradient:WSG** | 1.98E+02 | 2.00E-16 | n/a | n/a |
| **intercept:gradient:WSG** | 2.55E+02 | 2.00E-16 | n/a | n/a |
| **height:intercept:gradient:WSG** | -9.38E+00 | 2.00E-16 | n/a | n/a |

**Table S6** The candidate drivers used in this investigation divided into six groups (anthropogenic, climatic, edaphic, fire, geographic, and spatial). The possible effects of these drivers on forest carbon storage and sequestration, wood specific gravity and population structure has been provided.

| **Candidate Driver Name** | **Candidate Driver Description** | **Resolution** | **Data Source** | **Group** | **Carbon Storage and Sequestration (References)** | **Wood Specific Gravity**  **(References)** | **Population Structure**  **(References)** |
| --- | --- | --- | --- | --- | --- | --- | --- |
| Population pressure with decay constant of 41.6km | The population pressure experienced by an area, derived using a 41.6km decay constant | 0.1km | Raw pop data [[100](#_ENREF_100)] post-processed as described in [[99](#_ENREF_99)] | Anthropogenic | Logging and other forms of disturbance reduce the carbon stored in tropical forest. Increased disturbance will also result in increased carbon emissions.  [[107-110](#_ENREF_107)] | Logging and other forms of disturbance will result in a decrease of shade tolerant trees and a consequent increase in light demanding species. This will be associated with a reduction in WSG.  [[111](#_ENREF_111)] | Logging and other forms of disturbance will result in a decrease of shade tolerant trees and a consequent increase in light demanding species. Larger trees will be preferentially removed, leaving forests dominated by many small stems.  [[97](#_ENREF_97), [110](#_ENREF_110), [111](#_ENREF_111)] |
| Population pressure with decay constant of 20.8km | The population pressure experienced by an area, derived using a 20.8km decay constant |  |  |  |  |  |  |
| Population pressure with decay constant of 16.7km | The population pressure experienced by an area, derived using a 16.7km decay constant |  |  |  |  |  |  |
| Population pressure with decay constant of 12.5km | The population pressure experienced by an area, derived using a 12.5km decay constant |  |  |  |  |  |  |
| Population pressure with decay constant of 8.6km | The population pressure experienced by an area, derived using a 8.6km decay constant |  |  |  |  |  |  |
| Population pressure with decay constant of 4.2km | The population pressure experienced by an area, derived using a 4.1.7km decay constant |  |  |  |  |  |  |
| Population pressure with decay constant of 1.7km | The population pressure experienced by an area, derived using a 1.7km decay constant |  |  |  |  |  |  |
| Natural logarithm of the population pressure with decay constant of 41.6km | Log transformation of the population pressure experienced by an area, derived using a 41.6km decay constant. Unit addition avoids Logn(0) |  |  |  |  |  |  |
| Natural logarithm of the population pressure with decay constant of 20.8km | Log transformation of the population pressure experienced by an area, derived using a 20.8km decay constant. Unit addition avoids Logn(0) |  |  |  |  |  |  |
| Natural logarithm of the population pressure with decay constant of 16.7km | Log transformation of the population pressure experienced by an area, derived using a 16.7km decay constant. Unit addition avoids Logn(0) |  |  |  |  |  |  |
| Natural logarithm of the population pressure with decay constant of 12.5km | Log transformation of the population pressure experienced by an area, derived using a 12.5km decay constant. Unit addition avoids Logn(0) |  |  |  |  |  |  |
| Natural logarithm of the population pressure with decay constant of 8.6km | Log transformation of the population pressure experienced by an area, derived using a 8.6km decay constant. Unit addition avoids Logn(0) |  |  |  |  |  |  |
| Natural logarithm of the population pressure with decay constant of 4.2km | Log transformation of the population pressure experienced by an area, derived using a 4.1.7km decay constant. Unit addition avoids Logn(0) |  |  |  |  |  |  |
| Natural logarithm of the population pressure with decay constant of 1.7km | Log transformation of the population pressure experienced by an area, derived using a 1.7km decay constant. Unit addition avoids Logn(0) |  |  |  |  |  |  |
| Cost distance to Dar es Salaam | The cost distance to Dar es Salaam according to road type (A, B or C), protected areas and water bodies | 0.1km | N/A |  |  |  |  |
| Cost distance to market towns | The cost distance to market towns according to road type (A, B or C), protected areas and water bodies |  |  |  |  |  |  |
| Distance to roads | The euclidean distance to nearest A or B road |  |  |  |  |  |  |
| Distance to Dar es Salaam | The euclidean distance to Dar es Salaam |  |  |  |  |  |  |
| Distance to market towns | The euclidean distance to nearest market town (defined as a settlement with a population ≥ 5000 in 2002 census) |  |  |  |  |  |  |
| Natural logarithm of the cost distance to Dar es Salaam | Log transformation of the cost distance to Dar es Salaam. Unit addition avoids Logn(0) |  |  |  |  |  |  |
| Natural logarithm of the cost distance to market towns | Log transformation of the cost distance to a market town. Unit addition avoids Logn(0) |  |  |  |  |  |  |
| Natural logarithm of the cost distance to roads | Log transformation of the cost distance to nearest A or B road. Unit addition avoids Logn(0) |  |  |  |  |  |  |
| Historical logging | An attempt to record for each forest reserve an indication of the past logging history. Areas were assigned one of four categories: no logging, partially logged, clear felled and no data | 0.1km | [[112](#_ENREF_112)] |  |  |  |  |
| Governance | The governance of the land. Land categories were divided into those under national control, local control, joint management and unknown. | Various | [[113](#_ENREF_113)] |  |  |  |  |
| Mean annual temperature | The mean annual temperature derived from the mean monthly temperatures. Made more resolute using the elevation difference observed between the climate data digital elevation model and a higher resolution dataset | 0.1km | [[29](#_ENREF_29), [114](#_ENREF_114)] | Climatic | Temperatures increases and extreme droughts will decrease forest productivity, and therefore carbon storage and sequestration. However, carbon storage may increase with increased levels of gradual drought.  [[53](#_ENREF_53), [62](#_ENREF_62), [72](#_ENREF_72), [107](#_ENREF_107), [115](#_ENREF_115)] | WSG will increase with increased levels of drought. Other climatic factors may also be important.  [[62](#_ENREF_62), [72](#_ENREF_72), [89](#_ENREF_89)] | Changes in climate will alter species composition. Increasing amounts of precipitation and an increasing temperature range will result in increasing stem density.  [[62](#_ENREF_62), [97](#_ENREF_97), [98](#_ENREF_98), [116](#_ENREF_116)] |
| Mean annual maximum monthly temperature | The mean annual maximum temperature derived from the mean monthly maximum temperatures. Made more resolute using the elevation difference observed between the climate data digital elevation model and a higher resolution dataset |  |  |  |  |  |  |
| Mean annual minimum monthly temperature | The mean annual minimum temperature derived from the mean monthly minimum temperatures. Made more resolute using the elevation difference observed between the climate data digital elevation model and a higher resolution dataset |  |  |  |  |  |  |
| Mean annual monthly temperature range | The difference between the mean annual maximum and minimum temperatures |  |  |  |  |  |  |
| Mean maximum cumulative water deficit | Maximum mean cumulative water deficit (calculated as [[53](#_ENREF_53)]) | 4km | [[117](#_ENREF_117), [118](#_ENREF_118)] |  |  |  |  |
| Mean number of dry months annually | The average number of months annually that precipitation is exceeded by potential evapotranspiration |  |  |  |  |  |  |
| Wind speed | The mean annual wind speed 50m above the ground | 0.2km | [[119](#_ENREF_119)] |  |  |  |  |
| Total nitrogen content of the soil | The amount of organic carbon present in the soil to a depth of 1m | 10km | [[103](#_ENREF_103), [104](#_ENREF_104)] | Edaphic | More fertile areas will show greater growth rates and increased levels of carbon storage.  [[62](#_ENREF_62), [107](#_ENREF_107)] | More fertile areas will show lower WSG values.  [[62](#_ENREF_62)] | As soil fertility increases, stem density will increase. There will also be more large stems.  [[62](#_ENREF_62), [97](#_ENREF_97), [98](#_ENREF_98)] |
| Total carbon content of the soil | The amount of nitrogen present in the soil to a depth of 1m |  |  |  |  |  |  |
| Percentage sand content of the soil | The mean percentage mass of sand in the soil to a depth of 1m |  |  |  |  |  |  |
| Total available water capacity of the soil | The available water capacity of the soil to a depth of 1m |  |  |  |  |  |  |
| pH of the soil | The mean pH of the soil to a depth of 1m |  |  |  |  |  |  |
| Soil fertility | A mean measure of soil fertility to a 1m depth. Calculated as ((100-Aluminium saturation)/100)*effective cation exchange capacity |  |  |  |  |  |  |
| Mean burned area probability in the fourth quarter | The mean burnt area probability from January to March | 0.5km | [[120](#_ENREF_120)] | Fire | Areas that experience burns more frequently will show lower carbon storage levels. Overall, they will show lower rates of growth, but during recovery from a burn, growth rates may be increased.  [[121](#_ENREF_121), [122](#_ENREF_122)] | Areas that experience burns more frequently will show lower WSG values.  [[121](#_ENREF_121)] | An increased probability of fire will result in a decreasing stem density.  [[121-123](#_ENREF_121)] |
| Mean burned area probability in the third quarter | The mean burnt area probability from April to June |  |  |  |  |  |  |
| Mean burned area probability in the second quarter | The mean burnt area probability from July to September |  |  |  |  |  |  |
| Mean burned area probability in the first quarter | The mean burnt area probability from October to December |  |  |  |  |  |  |
| Annual mean burned area probability | The annual mean burned area probability |  |  |  |  |  |  |
| Aspect | The aspect of the slope | 0.1km | [[114](#_ENREF_114)] | Geographic | Higher solar radiation results in high levels of growth and carbon storage.  [[98](#_ENREF_98), [124](#_ENREF_124)] | Light demanding pioneers show lower wood density.  [[111](#_ENREF_111)] | Higher solar radiation levels may lead to increased tree densities and also an increased proportion of larger stems.  [[97](#_ENREF_97), [98](#_ENREF_98)] |
| Mean annual global horizontal solar radiation | The mean annual global horizontal solar radiation at ground level | 40km | [[125](#_ENREF_125), [126](#_ENREF_126)] |  |  |  |  |
| Spatial autocorrelation term 1 | Latitude + Longitude + Latitude*Latitude + Longitude*Longitude + Longitude*Latitude | 0.1km | N/A | Spatial | None - included in the model to help account for landscape scale spatial autocorrelation.  [[127-129](#_ENREF_127)] | None - included in the model to help account for landscape scale spatial autocorrelation.  [[127-129](#_ENREF_127)] | None - included in the model to help account for landscape scale spatial autocorrelation.  [[127-129](#_ENREF_127)] |
| Spatial autocorrelation term 2 | Latitude + Longitude + Latitude*Latitude + Longitude*Longitude |  |  |  |  |  |  |
| Spatial autocorrelation term 3 | Latitude + Longitude + Longitude*Latitude |  |  |  |  |  |  |
| Spatial autocorrelation term 4 | Latitude |  |  |  |  |  |  |
| Spatial autocorrelation term 5 | Longitude |  |  |  |  |  |  |
| Spatial autocorrelation term 6 | Latitude*Latitude |  |  |  |  |  |  |
| Spatial autocorrelation term 7 | Longitude*Longitude |  |  |  |  |  |  |
| Spatial autocorrelation term 8 | Longitude*Latitude |  |  |  |  |  |  |

**Table S7** The correlation coefficients of the continuous candidate drivers.

| **ID** | **Candidate Driver Name** | **1** | **2** | **3** | **4** | **5** | **6** | **7** | **8** | **9** | **10** | **11** | **12** | **13** | **14** | **15** | **16** | **17** | **18** | **19** | **20** | **21** | **22** | **23** | **24** | **25** | **26** | **27** | **28** | **29** | **30** | **31** | **32** | **33** | **34** | **35** | **36** | **37** | **38** | **39** | **40** | **41** | **42** | **43** | **44** | **45** | **46** | **47** | **48** | **49** | **50** |
| --- | --- | --- | --- | --- | --- | --- | --- | --- | --- | --- | --- | --- | --- | --- | --- | --- | --- | --- | --- | --- | --- | --- | --- | --- | --- | --- | --- | --- | --- | --- | --- | --- | --- | --- | --- | --- | --- | --- | --- | --- | --- | --- | --- | --- | --- | --- | --- | --- | --- | --- | --- |
| **1** | Population pressure with decay constant of 41.6km | 1.0 | 0.9 | 0.8 | 0.7 | 0.5 | 0.5 | 0.4 | 1.0 | 0.9 | 0.8 | 0.8 | 0.7 | 0.7 | 0.6 | -0.8 | -0.5 | -0.4 | -0.8 | -0.5 | -0.8 | -0.5 | -0.3 | -0.1 | 0.0 | -0.2 | 0.5 | -0.4 | 0.0 | 0.9 | -0.3 | -0.6 | 0.1 | -0.3 | -0.4 | 0.0 | -0.1 | -0.1 | NA | NA | -0.1 | -0.1 | -0.5 | 0.9 | 0.9 | 0.9 | 0.9 | 0.9 | -0.9 | 0.9 | 0.9 |
| **2** | Population pressure with decay constant of 20.8km | 0.9 | 1.0 | 1.0 | 0.9 | 0.8 | 0.7 | 0.6 | 0.9 | 1.0 | 0.9 | 0.9 | 0.9 | 0.8 | 0.7 | -0.8 | -0.5 | -0.5 | -0.9 | -0.7 | -0.8 | -0.5 | -0.3 | -0.2 | -0.1 | -0.2 | 0.4 | -0.5 | -0.2 | 0.7 | -0.2 | -0.4 | -0.1 | -0.2 | -0.2 | -0.1 | -0.1 | -0.1 | NA | NA | -0.1 | -0.1 | -0.6 | 0.8 | 0.8 | 0.7 | 0.8 | 0.8 | -0.8 | 0.8 | 0.7 |
| **3** | Population pressure with decay constant of 16.7km | 0.8 | 1.0 | 1.0 | 1.0 | 0.9 | 0.8 | 0.6 | 0.8 | 0.9 | 0.9 | 0.9 | 0.9 | 0.8 | 0.7 | -0.7 | -0.5 | -0.5 | -0.9 | -0.7 | -0.7 | -0.5 | -0.3 | -0.2 | -0.1 | -0.2 | 0.3 | -0.6 | -0.2 | 0.6 | -0.2 | -0.3 | -0.1 | -0.2 | -0.1 | -0.1 | -0.1 | -0.1 | NA | NA | -0.1 | -0.1 | -0.6 | 0.7 | 0.7 | 0.6 | 0.6 | 0.7 | -0.7 | 0.7 | 0.6 |
| **4** | Population pressure with decay constant of 12.5km | 0.7 | 0.9 | 1.0 | 1.0 | 1.0 | 0.9 | 0.6 | 0.7 | 0.8 | 0.9 | 0.9 | 0.9 | 0.8 | 0.7 | -0.6 | -0.5 | -0.5 | -0.8 | -0.6 | -0.7 | -0.5 | -0.3 | -0.2 | -0.2 | -0.2 | 0.3 | -0.6 | -0.3 | 0.5 | -0.1 | -0.2 | -0.1 | -0.1 | 0.0 | -0.2 | -0.1 | -0.1 | NA | NA | -0.1 | -0.1 | -0.6 | 0.6 | 0.6 | 0.5 | 0.5 | 0.6 | -0.5 | 0.6 | 0.5 |
| **5** | Population pressure with decay constant of 8.6km | 0.5 | 0.8 | 0.9 | 1.0 | 1.0 | 0.9 | 0.7 | 0.6 | 0.7 | 0.8 | 0.8 | 0.8 | 0.7 | 0.6 | -0.6 | -0.4 | -0.4 | -0.7 | -0.5 | -0.6 | -0.4 | -0.3 | -0.2 | -0.2 | -0.2 | 0.3 | -0.5 | -0.3 | 0.4 | -0.1 | -0.1 | -0.2 | -0.1 | 0.0 | -0.2 | -0.1 | -0.1 | NA | NA | -0.1 | -0.1 | -0.5 | 0.4 | 0.5 | 0.4 | 0.4 | 0.5 | -0.4 | 0.5 | 0.4 |
| **6** | Population pressure with decay constant of 4.2km | 0.5 | 0.7 | 0.8 | 0.9 | 0.9 | 1.0 | 0.8 | 0.5 | 0.7 | 0.7 | 0.7 | 0.7 | 0.7 | 0.6 | -0.5 | -0.4 | -0.4 | -0.6 | -0.5 | -0.5 | -0.4 | -0.3 | -0.2 | -0.2 | -0.2 | 0.2 | -0.4 | -0.3 | 0.4 | -0.1 | 0.0 | -0.1 | -0.1 | 0.1 | -0.2 | -0.1 | -0.1 | NA | NA | -0.1 | -0.1 | -0.4 | 0.4 | 0.4 | 0.3 | 0.3 | 0.4 | -0.4 | 0.4 | 0.3 |
| **7** | Population pressure with decay constant of 1.7km | 0.4 | 0.6 | 0.6 | 0.6 | 0.7 | 0.8 | 1.0 | 0.4 | 0.5 | 0.6 | 0.6 | 0.6 | 0.6 | 0.6 | -0.5 | -0.4 | -0.4 | -0.5 | -0.4 | -0.5 | -0.5 | -0.4 | -0.1 | 0.0 | -0.1 | 0.2 | -0.2 | -0.1 | 0.3 | -0.2 | -0.1 | -0.1 | -0.2 | 0.1 | -0.2 | 0.0 | 0.0 | NA | NA | 0.0 | 0.0 | -0.3 | 0.4 | 0.4 | 0.3 | 0.3 | 0.4 | -0.4 | 0.4 | 0.3 |
| **8** | Natural logarithm of the population pressure with decay constant of 41.6km | 1.0 | 0.9 | 0.8 | 0.7 | 0.6 | 0.5 | 0.4 | 1.0 | 0.9 | 0.8 | 0.8 | 0.7 | 0.7 | 0.6 | -0.8 | -0.5 | -0.4 | -0.8 | -0.5 | -0.8 | -0.5 | -0.3 | -0.2 | 0.0 | -0.2 | 0.5 | -0.4 | 0.0 | 0.9 | -0.3 | -0.6 | 0.1 | -0.3 | -0.4 | 0.0 | -0.1 | -0.1 | NA | NA | -0.1 | -0.1 | -0.5 | 0.9 | 0.9 | 0.9 | 0.9 | 0.9 | -0.9 | 0.9 | 0.9 |
| **9** | Natural logarithm of the population pressure with decay constant of 20.8km | 0.9 | 1.0 | 0.9 | 0.8 | 0.7 | 0.7 | 0.5 | 0.9 | 1.0 | 1.0 | 1.0 | 0.9 | 0.8 | 0.7 | -0.8 | -0.6 | -0.6 | -0.9 | -0.7 | -0.8 | -0.6 | -0.4 | -0.1 | 0.0 | -0.1 | 0.3 | -0.5 | -0.1 | 0.8 | -0.3 | -0.4 | -0.1 | -0.1 | -0.1 | -0.3 | -0.1 | -0.2 | NA | NA | -0.2 | -0.1 | -0.5 | 0.8 | 0.9 | 0.8 | 0.8 | 0.8 | -0.8 | 0.8 | 0.8 |
| **10** | Natural logarithm of the population pressure with decay constant of 16.7km | 0.8 | 0.9 | 0.9 | 0.9 | 0.8 | 0.7 | 0.6 | 0.8 | 1.0 | 1.0 | 1.0 | 1.0 | 0.8 | 0.7 | -0.8 | -0.6 | -0.6 | -0.9 | -0.8 | -0.8 | -0.6 | -0.4 | 0.0 | 0.0 | -0.1 | 0.2 | -0.5 | -0.1 | 0.7 | -0.3 | -0.3 | -0.1 | -0.1 | 0.0 | -0.3 | -0.1 | -0.2 | NA | NA | -0.2 | -0.1 | -0.5 | 0.8 | 0.8 | 0.7 | 0.7 | 0.8 | -0.7 | 0.8 | 0.7 |
| **11** | Natural logarithm of the population pressure with decay constant of 12.5km | 0.8 | 0.9 | 0.9 | 0.9 | 0.8 | 0.7 | 0.6 | 0.8 | 1.0 | 1.0 | 1.0 | 1.0 | 0.9 | 0.7 | -0.8 | -0.7 | -0.6 | -0.8 | -0.8 | -0.8 | -0.7 | -0.4 | 0.0 | 0.0 | -0.1 | 0.2 | -0.5 | -0.1 | 0.7 | -0.4 | -0.2 | -0.1 | -0.1 | 0.1 | -0.4 | -0.1 | -0.1 | NA | NA | -0.1 | -0.1 | -0.5 | 0.7 | 0.8 | 0.6 | 0.7 | 0.8 | -0.7 | 0.8 | 0.6 |
| **12** | Natural logarithm of the population pressure with decay constant of 8.6km | 0.7 | 0.9 | 0.9 | 0.9 | 0.8 | 0.7 | 0.6 | 0.7 | 0.9 | 1.0 | 1.0 | 1.0 | 1.0 | 0.9 | -0.9 | -0.7 | -0.7 | -0.8 | -0.7 | -0.8 | -0.7 | -0.5 | -0.1 | 0.0 | -0.1 | 0.2 | -0.4 | -0.1 | 0.6 | -0.4 | -0.2 | -0.1 | -0.1 | 0.2 | -0.5 | -0.1 | -0.1 | NA | NA | -0.1 | -0.1 | -0.4 | 0.7 | 0.8 | 0.6 | 0.7 | 0.7 | -0.7 | 0.7 | 0.6 |
| **13** | Natural logarithm of the population pressure with decay constant of 4.2km | 0.7 | 0.8 | 0.8 | 0.8 | 0.7 | 0.7 | 0.6 | 0.7 | 0.8 | 0.8 | 0.9 | 1.0 | 1.0 | 1.0 | -0.9 | -0.7 | -0.6 | -0.8 | -0.6 | -0.8 | -0.7 | -0.5 | -0.2 | -0.1 | -0.2 | 0.3 | -0.4 | -0.2 | 0.7 | -0.5 | -0.2 | -0.1 | -0.1 | 0.2 | -0.5 | 0.0 | 0.0 | NA | NA | 0.0 | -0.2 | -0.3 | 0.7 | 0.7 | 0.7 | 0.7 | 0.7 | -0.7 | 0.7 | 0.7 |
| **14** | Natural logarithm of the population pressure with decay constant of 1.7km | 0.6 | 0.7 | 0.7 | 0.7 | 0.6 | 0.6 | 0.6 | 0.6 | 0.7 | 0.7 | 0.7 | 0.9 | 1.0 | 1.0 | -0.9 | -0.7 | -0.5 | -0.7 | -0.5 | -0.8 | -0.7 | -0.5 | -0.2 | -0.1 | -0.3 | 0.4 | -0.3 | -0.1 | 0.6 | -0.5 | -0.1 | -0.1 | -0.1 | 0.2 | -0.5 | 0.0 | 0.0 | NA | NA | 0.0 | -0.2 | -0.2 | 0.7 | 0.7 | 0.6 | 0.7 | 0.7 | -0.7 | 0.7 | 0.6 |
| **15** | Cost distance to Dar es Salaam | -0.8 | -0.8 | -0.7 | -0.6 | -0.6 | -0.5 | -0.5 | -0.8 | -0.8 | -0.8 | -0.8 | -0.9 | -0.9 | -0.9 | 1.0 | 0.9 | 0.8 | 0.8 | 0.7 | 1.0 | 0.9 | 0.6 | -0.1 | -0.1 | 0.0 | -0.3 | 0.3 | 0.0 | -0.7 | 0.5 | 0.3 | 0.0 | 0.2 | 0.0 | 0.4 | 0.0 | 0.0 | NA | NA | 0.0 | 0.1 | 0.3 | -0.8 | -0.8 | -0.8 | -0.8 | -0.8 | 0.8 | -0.8 | -0.8 |
| **16** | Cost distance to market towns | -0.5 | -0.5 | -0.5 | -0.5 | -0.4 | -0.4 | -0.4 | -0.5 | -0.6 | -0.6 | -0.7 | -0.7 | -0.7 | -0.7 | 0.9 | 1.0 | 0.8 | 0.5 | 0.7 | 0.8 | 0.9 | 0.6 | -0.3 | -0.3 | -0.3 | 0.1 | 0.1 | -0.2 | -0.4 | 0.5 | 0.1 | 0.1 | 0.1 | -0.2 | 0.5 | 0.0 | 0.0 | NA | NA | 0.0 | 0.0 | 0.2 | -0.5 | -0.6 | -0.5 | -0.5 | -0.5 | 0.5 | -0.5 | -0.5 |
| **17** | Distance to roads | -0.4 | -0.5 | -0.5 | -0.5 | -0.4 | -0.4 | -0.4 | -0.4 | -0.6 | -0.6 | -0.6 | -0.7 | -0.6 | -0.5 | 0.8 | 0.8 | 1.0 | 0.4 | 0.6 | 0.7 | 0.7 | 0.9 | -0.1 | -0.1 | -0.1 | 0.0 | 0.2 | 0.0 | -0.3 | 0.3 | 0.3 | -0.1 | 0.1 | -0.1 | 0.4 | 0.0 | 0.0 | NA | NA | 0.0 | 0.1 | 0.2 | -0.4 | -0.5 | -0.4 | -0.4 | -0.4 | 0.4 | -0.4 | -0.4 |
| **18** | Distance to Dar es Salaam | -0.8 | -0.9 | -0.9 | -0.8 | -0.7 | -0.6 | -0.5 | -0.8 | -0.9 | -0.9 | -0.8 | -0.8 | -0.8 | -0.7 | 0.8 | 0.5 | 0.4 | 1.0 | 0.5 | 0.8 | 0.5 | 0.3 | 0.2 | 0.1 | 0.3 | -0.5 | 0.5 | 0.2 | -0.8 | 0.3 | 0.4 | 0.0 | 0.2 | 0.1 | 0.2 | 0.1 | 0.1 | NA | NA | 0.1 | 0.1 | 0.5 | -0.9 | -0.9 | -0.8 | -0.8 | -0.9 | 0.9 | -0.9 | -0.8 |
| **19** | Distance to market towns | -0.5 | -0.7 | -0.7 | -0.6 | -0.5 | -0.5 | -0.4 | -0.5 | -0.7 | -0.8 | -0.8 | -0.7 | -0.6 | -0.5 | 0.7 | 0.7 | 0.6 | 0.5 | 1.0 | 0.6 | 0.7 | 0.3 | -0.3 | -0.3 | -0.3 | 0.2 | 0.3 | -0.1 | -0.4 | 0.4 | 0.1 | 0.2 | -0.1 | -0.2 | 0.4 | 0.1 | 0.2 | NA | NA | 0.2 | 0.0 | 0.5 | -0.5 | -0.5 | -0.4 | -0.4 | -0.5 | 0.4 | -0.5 | -0.4 |
| **20** | Natural logarithm of the cost distance to Dar es Salaam | -0.8 | -0.8 | -0.7 | -0.7 | -0.6 | -0.5 | -0.5 | -0.8 | -0.8 | -0.8 | -0.8 | -0.8 | -0.8 | -0.8 | 1.0 | 0.8 | 0.7 | 0.8 | 0.6 | 1.0 | 0.8 | 0.6 | 0.0 | -0.1 | 0.0 | -0.3 | 0.3 | 0.0 | -0.8 | 0.5 | 0.4 | -0.1 | 0.2 | 0.1 | 0.4 | 0.0 | 0.1 | NA | NA | 0.1 | 0.1 | 0.3 | -0.9 | -0.9 | -0.8 | -0.9 | -0.9 | 0.9 | -0.9 | -0.8 |
| **21** | Natural logarithm of the cost distance to market towns | -0.5 | -0.5 | -0.5 | -0.5 | -0.4 | -0.4 | -0.5 | -0.5 | -0.6 | -0.6 | -0.7 | -0.7 | -0.7 | -0.7 | 0.9 | 0.9 | 0.7 | 0.5 | 0.7 | 0.8 | 1.0 | 0.7 | -0.3 | -0.3 | -0.3 | 0.1 | 0.1 | -0.2 | -0.4 | 0.6 | 0.1 | 0.1 | 0.1 | -0.3 | 0.6 | 0.0 | 0.1 | NA | NA | 0.1 | 0.0 | 0.1 | -0.5 | -0.6 | -0.5 | -0.5 | -0.6 | 0.5 | -0.6 | -0.5 |
| **22** | Natural logarithm of the cost distance to roads | -0.3 | -0.3 | -0.3 | -0.3 | -0.3 | -0.3 | -0.4 | -0.3 | -0.4 | -0.4 | -0.4 | -0.5 | -0.5 | -0.5 | 0.6 | 0.6 | 0.9 | 0.3 | 0.3 | 0.6 | 0.7 | 1.0 | 0.0 | 0.0 | 0.1 | -0.1 | 0.2 | 0.1 | -0.3 | 0.3 | 0.3 | -0.2 | 0.1 | -0.1 | 0.3 | 0.0 | 0.0 | NA | NA | 0.0 | 0.1 | 0.1 | -0.4 | -0.4 | -0.3 | -0.4 | -0.4 | 0.3 | -0.4 | -0.3 |
| **23** | Mean annual temperature | -0.1 | -0.2 | -0.2 | -0.2 | -0.2 | -0.2 | -0.1 | -0.2 | -0.1 | 0.0 | 0.0 | -0.1 | -0.2 | -0.2 | -0.1 | -0.3 | -0.1 | 0.2 | -0.3 | 0.0 | -0.3 | 0.0 | 1.0 | 1.0 | 1.0 | -0.6 | 0.3 | 0.4 | -0.1 | -0.4 | 0.1 | 0.0 | 0.0 | 0.2 | -0.3 | -0.1 | -0.1 | NA | NA | -0.1 | 0.2 | 0.1 | -0.1 | 0.0 | -0.2 | -0.1 | -0.1 | 0.2 | -0.1 | -0.2 |
| **24** | Mean annual maximum monthly temperature | 0.0 | -0.1 | -0.1 | -0.2 | -0.2 | -0.2 | 0.0 | 0.0 | 0.0 | 0.0 | 0.0 | 0.0 | -0.1 | -0.1 | -0.1 | -0.3 | -0.1 | 0.1 | -0.3 | -0.1 | -0.3 | 0.0 | 1.0 | 1.0 | 0.9 | -0.4 | 0.4 | 0.4 | 0.0 | -0.4 | 0.0 | 0.1 | 0.0 | 0.1 | -0.2 | 0.0 | -0.1 | NA | NA | -0.1 | 0.2 | 0.1 | 0.0 | 0.1 | 0.0 | 0.0 | 0.1 | 0.0 | 0.1 | 0.0 |
| **25** | Mean annual minimum monthly temperature | -0.2 | -0.2 | -0.2 | -0.2 | -0.2 | -0.2 | -0.1 | -0.2 | -0.1 | -0.1 | -0.1 | -0.1 | -0.2 | -0.3 | 0.0 | -0.3 | -0.1 | 0.3 | -0.3 | 0.0 | -0.3 | 0.1 | 1.0 | 0.9 | 1.0 | -0.7 | 0.3 | 0.4 | -0.2 | -0.3 | 0.2 | -0.1 | 0.1 | 0.3 | -0.3 | -0.1 | -0.1 | NA | NA | -0.1 | 0.2 | 0.1 | -0.2 | -0.1 | -0.2 | -0.2 | -0.1 | 0.3 | -0.1 | -0.2 |
| **26** | Mean annual monthly temperature range | 0.5 | 0.4 | 0.3 | 0.3 | 0.3 | 0.2 | 0.2 | 0.5 | 0.3 | 0.2 | 0.2 | 0.2 | 0.3 | 0.4 | -0.3 | 0.1 | 0.0 | -0.5 | 0.2 | -0.3 | 0.1 | -0.1 | -0.6 | -0.4 | -0.7 | 1.0 | -0.1 | -0.3 | 0.5 | 0.1 | -0.5 | 0.3 | -0.2 | -0.4 | 0.2 | 0.1 | 0.1 | NA | NA | 0.1 | -0.2 | -0.2 | 0.5 | 0.4 | 0.5 | 0.5 | 0.4 | -0.6 | 0.4 | 0.5 |
| **27** | Mean maximum cumulative water deficit | -0.4 | -0.5 | -0.6 | -0.6 | -0.5 | -0.4 | -0.2 | -0.4 | -0.5 | -0.5 | -0.5 | -0.4 | -0.4 | -0.3 | 0.3 | 0.1 | 0.2 | 0.5 | 0.3 | 0.3 | 0.1 | 0.2 | 0.3 | 0.4 | 0.3 | -0.1 | 1.0 | 0.6 | -0.3 | 0.1 | 0.3 | -0.1 | -0.1 | 0.1 | 0.0 | 0.1 | 0.1 | NA | NA | 0.1 | 0.1 | 0.4 | -0.3 | -0.4 | -0.3 | -0.3 | -0.3 | 0.3 | -0.3 | -0.3 |
| **28** | Mean number of dry months annually | 0.0 | -0.2 | -0.2 | -0.3 | -0.3 | -0.3 | -0.1 | 0.0 | -0.1 | -0.1 | -0.1 | -0.1 | -0.2 | -0.1 | 0.0 | -0.2 | 0.0 | 0.2 | -0.1 | 0.0 | -0.2 | 0.1 | 0.4 | 0.4 | 0.4 | -0.3 | 0.6 | 1.0 | 0.1 | -0.1 | 0.1 | -0.2 | -0.2 | 0.0 | 0.0 | 0.1 | 0.1 | NA | NA | 0.1 | 0.1 | 0.2 | 0.1 | 0.1 | 0.1 | 0.1 | 0.1 | -0.1 | 0.1 | 0.1 |
| **29** | Wind speed | 0.9 | 0.7 | 0.6 | 0.5 | 0.4 | 0.4 | 0.3 | 0.9 | 0.8 | 0.7 | 0.7 | 0.6 | 0.7 | 0.6 | -0.7 | -0.4 | -0.3 | -0.8 | -0.4 | -0.8 | -0.4 | -0.3 | -0.1 | 0.0 | -0.2 | 0.5 | -0.3 | 0.1 | 1.0 | -0.4 | -0.6 | 0.1 | -0.3 | -0.3 | -0.1 | -0.1 | -0.1 | NA | NA | -0.1 | -0.1 | -0.3 | 1.0 | 1.0 | 1.0 | 1.0 | 1.0 | -1.0 | 1.0 | 1.0 |
| **30** | Total nitrogen content of the soil | -0.3 | -0.2 | -0.2 | -0.1 | -0.1 | -0.1 | -0.2 | -0.3 | -0.3 | -0.3 | -0.4 | -0.4 | -0.5 | -0.5 | 0.5 | 0.5 | 0.3 | 0.3 | 0.4 | 0.5 | 0.6 | 0.3 | -0.4 | -0.4 | -0.3 | 0.1 | 0.1 | -0.1 | -0.4 | 1.0 | 0.2 | -0.4 | -0.1 | -0.4 | 0.8 | 0.1 | 0.1 | NA | NA | 0.1 | 0.1 | -0.2 | -0.4 | -0.4 | -0.4 | -0.4 | -0.4 | 0.4 | -0.4 | -0.4 |
| **31** | Total carbon content of the soil | -0.6 | -0.4 | -0.3 | -0.2 | -0.1 | 0.0 | -0.1 | -0.6 | -0.4 | -0.3 | -0.2 | -0.2 | -0.2 | -0.1 | 0.3 | 0.1 | 0.3 | 0.4 | 0.1 | 0.4 | 0.1 | 0.3 | 0.1 | 0.0 | 0.2 | -0.5 | 0.3 | 0.1 | -0.6 | 0.2 | 1.0 | -0.7 | 0.1 | 0.5 | -0.1 | 0.0 | 0.0 | NA | NA | 0.0 | 0.1 | 0.2 | -0.6 | -0.6 | -0.6 | -0.6 | -0.6 | 0.6 | -0.6 | -0.6 |
| **32** | Percentage sand content of the soil | 0.1 | -0.1 | -0.1 | -0.1 | -0.2 | -0.1 | -0.1 | 0.1 | -0.1 | -0.1 | -0.1 | -0.1 | -0.1 | -0.1 | 0.0 | 0.1 | -0.1 | 0.0 | 0.2 | -0.1 | 0.1 | -0.2 | 0.0 | 0.1 | -0.1 | 0.3 | -0.1 | -0.2 | 0.1 | -0.4 | -0.7 | 1.0 | 0.0 | -0.3 | -0.1 | 0.1 | 0.0 | NA | NA | 0.0 | -0.1 | 0.1 | 0.1 | 0.1 | 0.2 | 0.1 | 0.1 | -0.1 | 0.1 | 0.2 |
| **33** | Total available water capacity of the soil | -0.3 | -0.2 | -0.2 | -0.1 | -0.1 | -0.1 | -0.2 | -0.3 | -0.1 | -0.1 | -0.1 | -0.1 | -0.1 | -0.1 | 0.2 | 0.1 | 0.1 | 0.2 | -0.1 | 0.2 | 0.1 | 0.1 | 0.0 | 0.0 | 0.1 | -0.2 | -0.1 | -0.2 | -0.3 | -0.1 | 0.1 | 0.0 | 1.0 | 0.7 | -0.4 | 0.0 | 0.0 | NA | NA | 0.0 | 0.0 | 0.1 | -0.3 | -0.3 | -0.3 | -0.3 | -0.3 | 0.3 | -0.3 | -0.3 |
| **34** | pH of the soil | -0.4 | -0.2 | -0.1 | 0.0 | 0.0 | 0.1 | 0.1 | -0.4 | -0.1 | 0.0 | 0.1 | 0.2 | 0.2 | 0.2 | 0.0 | -0.2 | -0.1 | 0.1 | -0.2 | 0.1 | -0.3 | -0.1 | 0.2 | 0.1 | 0.3 | -0.4 | 0.1 | 0.0 | -0.3 | -0.4 | 0.5 | -0.3 | 0.7 | 1.0 | -0.8 | 0.0 | 0.0 | NA | NA | 0.0 | 0.0 | 0.2 | -0.3 | -0.2 | -0.3 | -0.3 | -0.2 | 0.3 | -0.2 | -0.3 |
| **35** | Soil fertility | 0.0 | -0.1 | -0.1 | -0.2 | -0.2 | -0.2 | -0.2 | 0.0 | -0.3 | -0.3 | -0.4 | -0.5 | -0.5 | -0.5 | 0.4 | 0.5 | 0.4 | 0.2 | 0.4 | 0.4 | 0.6 | 0.3 | -0.3 | -0.2 | -0.3 | 0.2 | 0.0 | 0.0 | -0.1 | 0.8 | -0.1 | -0.1 | -0.4 | -0.8 | 1.0 | 0.0 | 0.1 | NA | NA | 0.1 | 0.1 | -0.2 | -0.2 | -0.2 | -0.1 | -0.1 | -0.2 | 0.1 | -0.2 | -0.1 |
| **36** | Mean burned area probability in the fourth quarter | -0.1 | -0.1 | -0.1 | -0.1 | -0.1 | -0.1 | 0.0 | -0.1 | -0.1 | -0.1 | -0.1 | -0.1 | 0.0 | 0.0 | 0.0 | 0.0 | 0.0 | 0.1 | 0.1 | 0.0 | 0.0 | 0.0 | -0.1 | 0.0 | -0.1 | 0.1 | 0.1 | 0.1 | -0.1 | 0.1 | 0.0 | 0.1 | 0.0 | 0.0 | 0.0 | 1.0 | 0.6 | NA | NA | 0.9 | -0.1 | 0.2 | -0.1 | -0.1 | -0.1 | -0.1 | -0.1 | 0.1 | -0.1 | -0.1 |
| **37** | Mean burned area probability in the third quarter | -0.1 | -0.1 | -0.1 | -0.1 | -0.1 | -0.1 | 0.0 | -0.1 | -0.2 | -0.2 | -0.1 | -0.1 | 0.0 | 0.0 | 0.0 | 0.0 | 0.0 | 0.1 | 0.2 | 0.1 | 0.1 | 0.0 | -0.1 | -0.1 | -0.1 | 0.1 | 0.1 | 0.1 | -0.1 | 0.1 | 0.0 | 0.0 | 0.0 | 0.0 | 0.1 | 0.6 | 1.0 | NA | NA | 0.9 | -0.1 | 0.0 | -0.1 | -0.1 | -0.1 | -0.1 | -0.1 | 0.1 | -0.1 | -0.1 |
| **38** | Mean burned area probability in the second quarter | NA | NA | NA | NA | NA | NA | NA | NA | NA | NA | NA | NA | NA | NA | NA | NA | NA | NA | NA | NA | NA | NA | NA | NA | NA | NA | NA | NA | NA | NA | NA | NA | NA | NA | NA | NA | NA | 1.0 | NA | NA | NA | NA | NA | NA | NA | NA | NA | NA | NA | NA |
| **39** | Mean burned area probability in the first quarter | NA | NA | NA | NA | NA | NA | NA | NA | NA | NA | NA | NA | NA | NA | NA | NA | NA | NA | NA | NA | NA | NA | NA | NA | NA | NA | NA | NA | NA | NA | NA | NA | NA | NA | NA | NA | NA | NA | 1.0 | NA | NA | NA | NA | NA | NA | NA | NA | NA | NA | NA |
| **40** | Annual mean burned area probability | -0.1 | -0.1 | -0.1 | -0.1 | -0.1 | -0.1 | 0.0 | -0.1 | -0.2 | -0.2 | -0.1 | -0.1 | 0.0 | 0.0 | 0.0 | 0.0 | 0.0 | 0.1 | 0.2 | 0.1 | 0.1 | 0.0 | -0.1 | -0.1 | -0.1 | 0.1 | 0.1 | 0.1 | -0.1 | 0.1 | 0.0 | 0.0 | 0.0 | 0.0 | 0.1 | 0.9 | 0.9 | NA | NA | 1.0 | -0.1 | 0.1 | -0.1 | -0.1 | -0.1 | -0.1 | -0.1 | 0.1 | -0.1 | -0.1 |
| **41** | Aspect | -0.1 | -0.1 | -0.1 | -0.1 | -0.1 | -0.1 | 0.0 | -0.1 | -0.1 | -0.1 | -0.1 | -0.1 | -0.2 | -0.2 | 0.1 | 0.0 | 0.1 | 0.1 | 0.0 | 0.1 | 0.0 | 0.1 | 0.2 | 0.2 | 0.2 | -0.2 | 0.1 | 0.1 | -0.1 | 0.1 | 0.1 | -0.1 | 0.0 | 0.0 | 0.1 | -0.1 | -0.1 | NA | NA | -0.1 | 1.0 | 0.0 | -0.1 | -0.1 | -0.1 | -0.1 | -0.1 | 0.1 | -0.1 | -0.1 |
| **42** | Mean annual global horizontal solar radiation | -0.5 | -0.6 | -0.6 | -0.6 | -0.5 | -0.4 | -0.3 | -0.5 | -0.5 | -0.5 | -0.5 | -0.4 | -0.3 | -0.2 | 0.3 | 0.2 | 0.2 | 0.5 | 0.5 | 0.3 | 0.1 | 0.1 | 0.1 | 0.1 | 0.1 | -0.2 | 0.4 | 0.2 | -0.3 | -0.2 | 0.2 | 0.1 | 0.1 | 0.2 | -0.2 | 0.2 | 0.0 | NA | NA | 0.1 | 0.0 | 1.0 | -0.3 | -0.4 | -0.3 | -0.3 | -0.3 | 0.3 | -0.3 | -0.3 |
| **43** | Spatial autocorrelation term 1 | 0.9 | 0.8 | 0.7 | 0.6 | 0.4 | 0.4 | 0.4 | 0.9 | 0.8 | 0.8 | 0.7 | 0.7 | 0.7 | 0.7 | -0.8 | -0.5 | -0.4 | -0.9 | -0.5 | -0.9 | -0.5 | -0.4 | -0.1 | 0.0 | -0.2 | 0.5 | -0.3 | 0.1 | 1.0 | -0.4 | -0.6 | 0.1 | -0.3 | -0.3 | -0.2 | -0.1 | -0.1 | NA | NA | -0.1 | -0.1 | -0.3 | 1.0 | 1.0 | 1.0 | 1.0 | 1.0 | -1.0 | 1.0 | 1.0 |
| **44** | Spatial autocorrelation term 2 | 0.9 | 0.8 | 0.7 | 0.6 | 0.5 | 0.4 | 0.4 | 0.9 | 0.9 | 0.8 | 0.8 | 0.8 | 0.7 | 0.7 | -0.8 | -0.6 | -0.5 | -0.9 | -0.5 | -0.9 | -0.6 | -0.4 | 0.0 | 0.1 | -0.1 | 0.4 | -0.4 | 0.1 | 1.0 | -0.4 | -0.6 | 0.1 | -0.3 | -0.2 | -0.2 | -0.1 | -0.1 | NA | NA | -0.1 | -0.1 | -0.4 | 1.0 | 1.0 | 1.0 | 1.0 | 1.0 | -1.0 | 1.0 | 1.0 |
| **45** | Spatial autocorrelation term 3 | 0.9 | 0.7 | 0.6 | 0.5 | 0.4 | 0.3 | 0.3 | 0.9 | 0.8 | 0.7 | 0.6 | 0.6 | 0.7 | 0.6 | -0.8 | -0.5 | -0.4 | -0.8 | -0.4 | -0.8 | -0.5 | -0.3 | -0.2 | 0.0 | -0.2 | 0.5 | -0.3 | 0.1 | 1.0 | -0.4 | -0.6 | 0.2 | -0.3 | -0.3 | -0.1 | -0.1 | -0.1 | NA | NA | -0.1 | -0.1 | -0.3 | 1.0 | 1.0 | 1.0 | 1.0 | 1.0 | -1.0 | 1.0 | 1.0 |
| **46** | Spatial autocorrelation term 4 | 0.9 | 0.8 | 0.6 | 0.5 | 0.4 | 0.3 | 0.3 | 0.9 | 0.8 | 0.7 | 0.7 | 0.7 | 0.7 | 0.7 | -0.8 | -0.5 | -0.4 | -0.8 | -0.4 | -0.9 | -0.5 | -0.4 | -0.1 | 0.0 | -0.2 | 0.5 | -0.3 | 0.1 | 1.0 | -0.4 | -0.6 | 0.1 | -0.3 | -0.3 | -0.1 | -0.1 | -0.1 | NA | NA | -0.1 | -0.1 | -0.3 | 1.0 | 1.0 | 1.0 | 1.0 | 1.0 | -1.0 | 1.0 | 1.0 |
| **47** | Spatial autocorrelation term 5 | 0.9 | 0.8 | 0.7 | 0.6 | 0.5 | 0.4 | 0.4 | 0.9 | 0.8 | 0.8 | 0.8 | 0.7 | 0.7 | 0.7 | -0.8 | -0.5 | -0.4 | -0.9 | -0.5 | -0.9 | -0.6 | -0.4 | -0.1 | 0.1 | -0.1 | 0.4 | -0.3 | 0.1 | 1.0 | -0.4 | -0.6 | 0.1 | -0.3 | -0.2 | -0.2 | -0.1 | -0.1 | NA | NA | -0.1 | -0.1 | -0.3 | 1.0 | 1.0 | 1.0 | 1.0 | 1.0 | -1.0 | 1.0 | 1.0 |
| **48** | Spatial autocorrelation term 6 | -0.9 | -0.8 | -0.7 | -0.5 | -0.4 | -0.4 | -0.4 | -0.9 | -0.8 | -0.7 | -0.7 | -0.7 | -0.7 | -0.7 | 0.8 | 0.5 | 0.4 | 0.9 | 0.4 | 0.9 | 0.5 | 0.3 | 0.2 | 0.0 | 0.3 | -0.6 | 0.3 | -0.1 | -1.0 | 0.4 | 0.6 | -0.1 | 0.3 | 0.3 | 0.1 | 0.1 | 0.1 | NA | NA | 0.1 | 0.1 | 0.3 | -1.0 | -1.0 | -1.0 | -1.0 | -1.0 | 1.0 | -1.0 | -1.0 |
| **49** | Spatial autocorrelation term 7 | 0.9 | 0.8 | 0.7 | 0.6 | 0.5 | 0.4 | 0.4 | 0.9 | 0.8 | 0.8 | 0.8 | 0.7 | 0.7 | 0.7 | -0.8 | -0.5 | -0.4 | -0.9 | -0.5 | -0.9 | -0.6 | -0.4 | -0.1 | 0.1 | -0.1 | 0.4 | -0.3 | 0.1 | 1.0 | -0.4 | -0.6 | 0.1 | -0.3 | -0.2 | -0.2 | -0.1 | -0.1 | NA | NA | -0.1 | -0.1 | -0.3 | 1.0 | 1.0 | 1.0 | 1.0 | 1.0 | -1.0 | 1.0 | 1.0 |
| **50** | Spatial autocorrelation term 8 | 0.9 | 0.7 | 0.6 | 0.5 | 0.4 | 0.3 | 0.3 | 0.9 | 0.8 | 0.7 | 0.6 | 0.6 | 0.7 | 0.6 | -0.8 | -0.5 | -0.4 | -0.8 | -0.4 | -0.8 | -0.5 | -0.3 | -0.2 | 0.0 | -0.2 | 0.5 | -0.3 | 0.1 | 1.0 | -0.4 | -0.6 | 0.2 | -0.3 | -0.3 | -0.1 | -0.1 | -0.1 | NA | NA | -0.1 | -0.1 | -0.3 | 1.0 | 1.0 | 1.0 | 1.0 | 1.0 | -1.0 | 1.0 | 1.0 |

**Table S8** The mean (and 95% CI) look-up table values used to estimate the gradient of the power law relationship, the intercept of the power law relationship and WSG. Carbon storage look-up values were taken directly from Table 3.9. Due to the low sample size (n=43), the carbon sequestration look-up values were not separated by land cover and a value of -2.39 (-6.35 to 1.16) Mg ha^-1^ yr^-1^ was used throughout the study area.

| **Land cover [**[**130**](#_ENREF_130)**]** | **Sample Size** | **WSG** | **The gradient of the power law relationship** | **The intercept of the power law relationship** |
| --- | --- | --- | --- | --- |
| **Lowland Forest (<1000m)** | 579 | 0.60 (0.59 to 0.61) | -0.91 (-0.98 to -0.85) | 3.99 (3.72 to 4.28) |
| **Sub-montane forest (1000-1500m)** | 492 | 0.55 (0.55 to 0.56) | -1.25 (-1.30 to -1.20) | 5.68 (5.41 to 5.96) |
| **Montane Forest (1500-2000m)** | 146 | 0.60 (0.60 to 0.61) | -1.60 (-1.71 to -1.49) | 6.94 (6.46 to 7.43) |
| **Upper-montane forest (>2000m)** | 27 | 0.61 (0.59 to 0.62) | -1.67 (-2.00 to -1.31) | 7.13 (5.63 to 8.62) |
| **Forest mosaic** | 1* | 0.49 | -1.81 | 8.67 |
| **Closed Woodland** | 38 | 0.64 (0.62 to 0.67) | -1.56 (-1.86 to -1.33) | 6.67 (5.77 to 7.84) |
| **Open Woodland** | 67 | 0.61 (0.59 to 0.62) | -1.45 (-1.71 to -1.20) | 6.40 (5.24 to 7.51) |

*Confidence intervals not calculated due to low sample size. Included in the table to highlight this lack of data.

**Table S9** DBH-height equation forms tested. *H* is height, *D* is DBH and *a,* *b* and *c* are constant coefficients to be estimated [[25](#_ENREF_25), [131](#_ENREF_131)]. Equation forms that failed to stabilise are indicated by n/a.

| **Equation name** | **Form** | **Parameter interpretation** | **Model statistics without temperature** | **Model statistics including temperature** | **References** |
| --- | --- | --- | --- | --- | --- |
| **Power** |  | No biological interpretation | AIC = 104035.3  R^2^ = 0.61  P-value < 0.001 | AIC = 103710.1  R^2^ = 0.62  P-value < 0.001 | [[132](#_ENREF_132), [133](#_ENREF_133)] |
| **Two parameter exponential** |  | a = maximum height  b = rate parameter | a = 34.3  AIC = 103722.0  R^2^ = 0.61  P-value < 0.001 | a = -2.8 + 1.5 * MAT  AIC =103436.4  R^2^ = 0.62  P-value < 0.001 | [[134](#_ENREF_134)] |
| **Three parameter exponential** |  | a = maximum height  b = height range  c = rate parameter | n/a | a = -8.2 + 1.5 * MAT  AIC =104463.0  R^2^ = 0.64  P-value < 0.001 | [[135](#_ENREF_135), [136](#_ENREF_136)] |
| **Gompertz** |  | a = maximum height  b = no biological interpretation (reflects choice of zero D)  c = rate parameter  Inflection at D/e | n/a | a = 1.0 + 1.2 * MAT  AIC =102935.1  R^2^ = 0.65  P-value < 0.001 | [[137](#_ENREF_137), [138](#_ENREF_138)] |
| **Logistic** |  | a = maximum height  b = no biological interpretation (reflects choice of zero D)  c = rate parameter  Inflection at D/2 | a = 27.7  AIC =103491.7  R^2^ = 0.65  P-value < 0.001 | a = -20.0 + 2.2 * MAT  AIC =103058.0  R^2^ = 0.65  P-value < 0.001 | [[137](#_ENREF_137), [138](#_ENREF_138)] |
| **Weibull** |  | a = maximum height  b = rate parameter  c = shape parameter | n/a | n/a | [[139](#_ENREF_139), [140](#_ENREF_140)] |

**Table S10** The PCA axis of environmental variables spanned by my 1,611 plots. Axis 1 is dominated by temperature variables. The correlation coefficient between MAT and other variables is also shown. Elevation and anthropogenic disturbance (not shown) had correlation coefficients ranges of 0.92 to 0.96 and -0.30 to 0.35 respectively.

| **Variable** | **Description** | **Resoltion** | **Source** | **Axis 1** | **Axis 2** | **Axis 3** | **Axis 4** | **Axis 5** | **Correlation with MAT** |
| --- | --- | --- | --- | --- | --- | --- | --- | --- | --- |
| **Mean burned area probability in the fourth quarter** | The mean burnt area probability from January to March | 0.5km | [[120](#_ENREF_120)] | 0.028817 | 0.436769 | -0.22405 | 0.217956 | -0.11453 | -0.05 |
| **Mean burned area probability in the third quarter** | The mean burnt area probability from April to June |  |  | 0.042253 | 0.427735 | -0.20595 | 0.200203 | -0.1424 | -0.07 |
| **Annual mean burned area probability** | The annual mean burned area probability |  |  | 0.038842 | 0.484005 | -0.24165 | 0.235007 | -0.1418 | -0.07 |
| **Total nitrogen content of the soil** | The amount of organic carbon present in the soil to a depth of 1m | 10km | [[103](#_ENREF_103), [104](#_ENREF_104)] | 0.187956 | 0.276185 | 0.22216 | -0.37162 | -0.13668 | -0.35 |
| **Total carbon content of the soil** | The amount of nitrogen present in the soil to a depth of 1m |  |  | -0.21043 | 0.261661 | 0.396431 | -0.09508 | 0.107052 | 0.14 |
| **Total available water capacity of the soil** | The available water capacity of the soil to a depth of 1m |  |  | -0.13038 | -0.00075 | 0.265522 | 0.357736 | -0.14687 | 0.02 |
| **pH of the soil** | The mean pH of the soil to a depth of 1m |  |  | -0.28239 | -0.00214 | 0.303893 | 0.377933 | 0.100051 | 0.22 |
| **Percentage sand content of the soil** | The mean percentage mass of sand in the soil to a depth of 1m |  |  | 0.087656 | -0.19291 | -0.35074 | 0.168144 | -0.21462 | 0.01 |
| **Soil fertility** | A mean measure of soil fertility to a 1m depth. Calculated as ((100-Aluminium saturation)/100)*effective cation exchange capacity |  |  | 0.233446 | 0.190747 | -0.03035 | -0.47566 | -0.13187 | -0.27 |
| **Mean annual global horizontal solar radiation** | The mean annual global horizontal solar radiation at ground level | 40km | [[125](#_ENREF_125), [126](#_ENREF_126)] | -0.15015 | 0.150255 | 0.01957 | 0.099889 | 0.364822 | 0.11 |
| **Aspect** | The aspect of the slope | 0.1km | [[114](#_ENREF_114)] | -0.09031 | -0.01527 | 0.043953 | -0.22473 | -0.17124 | 0.17 |
| **Wind speed** | The mean annual wind speed 50m above the ground | 0.2km | [[119](#_ENREF_119)] | 0.151085 | -0.25267 | -0.30176 | 0.087243 | 0.292845 | -0.14 |
| **Mean maximum cumulative water deficit** | Maximum mean cumulative water deficit (calculated as [[53](#_ENREF_53)]) | 4km | [[117](#_ENREF_117), [118](#_ENREF_118)] | -0.2134 | 0.227975 | -0.12155 | -0.15869 | 0.480611 | 0.34 |
| **Mean number of dry months annually** | The average number of months annually that precipitation is exceeded by potential evapotranspiration |  |  | -0.22289 | 0.126725 | -0.25759 | -0.19297 | 0.422544 | 0.44 |
| **Mean annual temperature** | The mean annual temperature derived from the mean monthly temperatures. Made more resolute using the elevation difference observed between the climate data digital elevation model and a higher resolution dataset | 0.1km | [[29](#_ENREF_29), [114](#_ENREF_114)] | -0.4073 | -0.06801 | -0.21286 | -0.12644 | -0.19817 | 1 |
| **Mean annual maximum monthly temperature** | The mean annual maximum temperature derived from the mean monthly maximum temperatures. Made more resolute using the elevation difference observed between the climate data digital elevation model and a higher resolution dataset |  |  | -0.36416 | -0.0943 | -0.29076 | -0.12279 | -0.16486 | 0.97 |
| **Mean annual minimum monthly temperature** | The mean annual minimum temperature derived from the mean monthly minimum temperatures. Made more resolute using the elevation difference observed between the climate data digital elevation model and a higher resolution dataset |  |  | -0.42556 | -0.04709 | -0.14756 | -0.12722 | -0.21809 | 0.98 |
| **Mean annual monthly temperature range** | The difference between the mean annual maximum and minimum temperatures |  |  | 0.359161 | -0.05696 | -0.17193 | 0.083342 | 0.222735 | 0.98 |
| **Standard deviation** |  |  |  | 2.129 | 1.6895 | 1.5965 | 1.5546 | 1.11668 |  |
| **Proportion of Variance** |  |  |  | 0.2518 | 0.1586 | 0.1416 | 0.1343 | 0.06928 |  |
| **Cumulative Proportion** |  |  |  | 0.2518 | 0.4104 | 0.552 | 0.6863 | 0.75555 |  |

**Table S11** Mean biomass estimates for forested land within my study area using moist and dry forest allometric equations [[33](#_ENREF_33)].

| **Allometric equation** | **Inputs** | **Biomass Estimate, Mg ha^-1^ (95% CI range)** |
| --- | --- | --- |
| Moist forest equation | DBH, height and WSG | 314.2 (300.6-327.6) |
| Moist forest equation | DBH and WSG | 495.6 (475.8-515.2) |
| Dry forest equation | DBH, height and WSG | 280.2 (269.0-291.2) |
| Dry forest equation | DBH and WSG | 262.4 (253.4-271.6) |

**Table S12** The maxima associated with the Gompertz model fits for a range of DBH measurements.

| **DBH (cm)** | **Estimate maximum height (m)** | **MAT where maximum height occurs (^o^C)** |
| --- | --- | --- |
| 10 | 11.5 (8.3-14.3) | 12.0 |
| 20 | 13.9 (8.8-20.9) | 16.2 |
| 40 | 19.7 (9.7-41.3) | 22.1 |
| 60 | 29.0 (10.6-62.9) | 26.2 |
| 80 | 29.7 (11.4-81.5) | 29.3 |
| 100 | 33.6 (12.1-96.7) | 31.8 |
| 150 | 40.5 (13.5-123.5) | 36.1 |
| 200 | 45.1 (14.5-140.4) | 39.0 |

**References**

1. Mayaux P, Eva H, Gallego J, Strahler AH, Herold M, Agrawal S, Naumov S, De Miranda EE, Di Bella CM, Ordoyne C, et al: **Validation of the global land cover 2000 map.** *Geoscience and Remote Sensing, IEEE Transactions on* 2006, **44:**1728-1739.

2. Mumby PJ, Green EP, Edwards AJ, Clark CD: **The cost-effectiveness of remote sensing for tropical coastal resources assessment and management.** *Journal of Environmental Management* 1999, **55:**157-166.

3. Hardcastle P, Baird D, Harden V, Abbot PG, O’Hara P, Palmer JR, Roby A, Haüsler T, Ambia V, Branthomme A, et al: **Capability and cost assessment of the major forest nations to measure and monitor their forest carbon.** Edinburg, Scotland: LTS International Ltd; 2008.

4. **Science for a changing world** [<http://www.usgs.gov/default.asp>]

5. Asner GP: **Cloud cover in Landsat observations of the Brazilian Amazon.** *International Journal of Remote Sensing* 2001, **22:**3855-3862.

6. Muukkonen P, Heiskanen J: **Biomass estimation over a large area based on standwise forest inventory data and ASTER and MODIS satellite data: A possibility to verify carbon inventories.** *Remote Sensing of Environment* 2007, **107:**617-624.

7. Thenkabail PS, Enclona EA, Ashton MS, Legg C, De Dieu MJ: **Hyperion, IKONOS, ALI, and ETM+ sensors in the study of African rainforests.** *Remote Sensing of Environment* 2004, **90:**23-43.

8. Lu D: **Aboveground biomass estimation using Landsat TM data in the Brazilian Amazon.** *International Journal of Remote Sensing* 2005, **26:**2509-2525.

9. Foody GM, Cutler MEJ: **Tree biodiversity in protected and logged Bornean tropical rain forests and its measurement by satellite remote sensing.** *Journal of Biogeography* 2003, **30:**1053-1066.

10. Sánchez-Azofeifa GA, Castro-Esau KL, Kurz WA, Joyce A: **Monitoring carbon stocks in the tropics and the remote sensing operational limitations: from local to regional projects.** *Ecological Applications* 2009, **19:**480-494.

11. Waring RH, Way J, Hunt ER, Morrissey L, Ranson KJ, Weishampel JF, Oren R, Franklin SE: **Imaging Radar for Ecosystem Studies.** *BioScience* 1995, **45:**715-723.

12. Brown S, Pearson T, Slaymaker D, Ambagis S, Moore N, Novelo D, Sabido W: **Creating A Virtual Tropical Forest From Three-Dimensional Aerial Imagery To Estimate Carbon Stocks.** *Ecological Applications* 2005, **15:**1083-1095.

13. Kellndorfer J, Walker W, Pierce L, Dobson C, Fites JA, Hunsaker C, Vona J, Clutter M: **Vegetation height estimation from Shuttle Radar Topography Mission and National Elevation Datasets.** *Remote Sensing of Environment* 2004, **93:**339-358.

14. Collins JN, Hutley LB, Williams RJ, Boggs G, Bell D, Bartolo R: **Estimating landscape-scale vegetation carbon stocks using airborne multi-frequency polarimetric synthetic aperture radar (SAR) in the savannahs of north Australia.** *International Journal of Remote Sensing* 2009, **30:**1141-1159.

15. Omasa K, Hosoi F, Konishi A: **3D lidar imaging for detecting and understanding plant responses and canopy structure.** *Journal of Experimental Botany* 2007, **58:**881-898.

16. Lefsky MA, Cohen WB, Acker SA, Parker GG, Spies TA, Harding D: **Lidar Remote Sensing of the Canopy Structure and Biophysical Properties of Douglas-Fir Western Hemlock Forests.** *Remote Sensing of Environment* 1999, **70:**339-361.

17. Baccini A, Goetz SJ, Walker WS, Laporte NT, Sun M, Sulla-Menashe D, Hackler J, Beck PSA, Dubayah R, Friedl MA, et al: **Estimated carbon dioxide emissions from tropical deforestation improved by carbon-density maps.** *Nature Clim Change* 2012, **2:**182-185.

18. Saatchi SS, Harris NL, Brown S, Lefsky M, Mitchard ETA, Salas W, Zutta BR, Buermann W, Lewis SL, Hagen S, et al: **Benchmark map of forest carbon stocks in tropical regions across three continents.** *Proceedings of the National Academy of Sciences* 2011, **108:**9899-9904.

19. Means JE, Acker SA, Harding DJ, Blair JB, Lefsky MA, Cohen WB, Harmon ME, McKee WA: **Use of Large-Footprint Scanning Airborne Lidar To Estimate Forest Stand Characteristics in the Western Cascades of Oregon.** *Remote Sensing of Environment* 1999, **67:**298-308.

20. Drake JB, Knox RG, Dubayah RO, Clark DB, Condit R, Blair JB, Hofton M: **Above-ground biomass estimation in closed canopy Neotropical forests using lidar remote sensing: factors affecting the generality of relationships.** *Global Ecology and Biogeography* 2003, **12:**147-159.

21. Reutebuch S, McGaughey R, Andersen H-E, Carson W: **Accuracy of a high-resolution lidar terrain model under a conifer forest canopy** *Canadian Journal of Remote Sensing* 2003, **29:**527-535.

22. Hese S, Lucht W, Schmullius C, Barnsley M, Dubayah R, Knorr D, Neumann K, Riedel T, Schröter K: **Global biomass mapping for an improved understanding of the CO2 balance—the Earth observation mission Carbon-3D.** *Remote Sensing of Environment* 2005, **94:**94-104.

23. Gibbs HK, Brown S, Niles JO, Foley JA: **Monitoring and estimating tropical forest carbon stocks: making REDD a reality.** *Environmental Research Letters* 2007, **2**.

24. Feldpausch TR, Banin L, Phillips OL, Baker TR, Lewis SL, Quesada CA, Affum-Baffoe K, Arets EJMM, Berry NJ, Bird M, et al: **Height-diameter allometry of tropical forest trees.** *Biogeosciences* 2011, **8:**1081-1106.

25. Banin L, Feldpausch TR, Phillips OL, Baker TR, Lloyd J, Affum-Baffoe K, Arets EJMM, Berry NJ, Bradford M, Breinen RJW, et al: **What controls tropical forest architecture? Testing environmental, structural and floristic drivers.** *Global Ecology and Biogeography* 2012, **21:**1179-1190.

26. Goldstein ML, Morris SA, Yen GG: **Problems with fitting to the power-law distribution.** *The European Physical Journal B - Condensed Matter and Complex Systems* 2004, **41:**255-258.

27. Lovett JC: **Eastern Arc moist forest flora.** In *Biogeography and Ecology of the Rainforests of Eastern Africa* (Lovett JC, Wasser SK eds.). pp. 33-55; 1993:33-55.

28. Marshall AR, Willcock S, Lovett JC, Balmford A, Burgess ND, Latham JE, Munishi PKT, Platts PJ, Salter R, Shirima DD, Lewis SL: **Measuring and modelling above-ground carbon storage and tree allometry along an elevation gradient.** *Biological Conservation* 2012.

29. Hijmans RJ, Cameron SE, Parra JL, Jones PG, Jarvis A: **Very high resolution interpolated climate surfaces for global land areas.** *International Journal of Climatology* 2005, **25:**1965-1978.

30. Zanne AE, Lopez-Gonzalez G, Coomes DA, Ilic J, Jansen S, Lewis SL, Miller RB, Swenson NG, Wiemann MC, Chave J: **Global wood density database.** Dryad. Identifier <http://hdl.handle.net/10255/dryad.235> [Accessed 5/12/2008]; 2009.

31. Baker TR, Phillips OL, Malhi Y, Almeida S, Arroyo L, Di Fiore A, Erwin T, Killeen TJ, Laurance SG, Laurance WF, et al: **Variation in wood density determines spatial patterns in Amazonian forest biomass.** *Global Change Biology* 2004, **10:**545-562.

32. Djomo AN, Ibrahima A, Saborowski J, Gravenhorst G: **Allometric equations for biomass estimations in Cameroon and pan moist tropical equations including biomass data from Africa.** *Forest Ecology and Management* 2010, **260:**1873-1885.

33. Chave J, Andalo C, Brown S, Cairns MA, Chambers JQ, Eamus D, Folster H, Fromard F, Higuchi N, Kira T, et al: **Tree allometry and improved estimation of carbon stocks and balance in tropical forests.** *Oecologia* 2005, **145:**87-99.

34. Lewis SL, Lopez-Gonzalez G, Sonke B, Affum-Baffoe K, Baker TR, Ojo LO, Phillips OL, Reitsma JM, White L, Comiskey JA, et al: **Increasing carbon storage in intact African tropical forests.** *Nature* 2009, **457:**1003-1006.

35. Lovett JC: **Classification and status of the moist forests of Tanzania.** *Mitteilungen aus dem Institut für Allgemeine Botanik Hamburg* 1990, **23a:**287-300.

36. Mutai CC, Ward MN, Colman AW: **Towards the prediction of the East Africa short rains based on sea-surface temperature–atmosphere coupling.** *International Journal of Climatology* 1998, **18:**975-997.

37. Frontier-Tanzania: **Methods Manual.** In *Conervation and Management of the Eastern Arc Mountain Forests, Tanzania: Uluguru Component Biodiversity Survey 2005* (Bracebridge CN, Fanning E, Howell KM, St-John FAV eds.), vol. 1. pp. 76. Dar es Salaam, Tanzania: Frontier-Tanzania, University of Dar es Salaam & Society for Environmental Exploration; 2005:76.

38. Chave J: **Measuring tree height for tropical forest trees - a field manual.** In *Sixth Framework Programme (2002-2006)* (Pan-Amazonia ed. Toulouse, France: Universite Paul Sabatier; 2005.

39. Lovett JC, Marshall AR, Carr J: **Changes in tropical forest vegetation along an altitudinal gradient in the Udzungwa Mountains National Park, Tanzania.** *African Journal of Ecology* 2006, **44:**478-490.

40. Hall JB: **Multiple-nearest-tree sampling in an ecological survey of Afromontane catchment forest.** *Forest Ecology and Management* 1991, **42:**245-266.

41. Lovett JC: **Elevational and Latitudinal Changes in Tree Associations and Diversity in the Eastern Arc Mountains of Tanzania** *Journal of Tropical Ecology* 1996, **12:**629-650.

42. Lovett JC: **Tanzanian forest tree plot diversity and elevation.** *Journal of Tropical Ecology* 1999, **15:**689-694.

43. Shirima DD, Munishi PKT, Lewis SL, Burgess ND, Marshall AR, Balmford A, Swetnam RD, Zahabu EM: **Carbon storage, structure and composition of miombo woodlands in Tanzania’s Eastern Arc Mountains.** *African Journal of Ecology* 2011, **49:**332-342.

44. Phillips OL, Malhi Y, Vinceti B, Baker T, Lewis SL, Higuchi N, Laurance WF, Vargas PN, Martinez RV, Laurance S, et al: **Changes in growth of tropical forests: Evaluating potential biases.** *Ecological Applications* 2002, **12:**576-587.

45. Enquist BJ, Niklas KJ: **Invariant scaling relations across tree-dominated communities.** *Nature* 2001, **410:**655-660.

46. Li H-T, Han X-G, Wu J-G: **Lack of Evidence for 3/4 Scaling of Metabolism in Terrestrial Plants.** *Journal of Integrative Plant Biology* 2005, **47:**1173-1183.

47. Enquist BJ, West GB, Brown JH: **Extensions and evaluations of a general quantitative theory of forest structure and dynamics.** *Proceedings of the National Academy of Sciences* 2009, **106:**7046-7051.

48. Marshall AR, Willcock S, Platts PJ, Lovett JC, Balmford A, Burgess ND, Latham JE, Munishi PKT, Salter R, Shirima DD, Lewis SL: **Measuring and modelling above-ground carbon and tree allometry along a tropical elevation gradient.** *Biological Conservation* 2012, **154:**20-33.

49. Kuebler C: **Standardized Vegetation Monitoring Protocol.** Washington DC: Tropical Ecology, Assessment, and Monitoring Initiative, Centre for Applied Biodiversity Science, Conservation International,; 2003.

50. Chave J, Condit R, Muller-Landau HC, Thomas SC, Ashton PS, Bunyavejchewin S, Co LL, Dattaraja HS, Davies SJ, Esufali S, et al: **Assessing Evidence for a Pervasive Alteration in Tropical Tree Communities.** *PLoS Biol* 2008, **6:**e45.

51. Angiosperm Phylogeny Group: **An update of the Angiosperm Phylogeny Group classification for the orders and families of flowering plants: APG II.** *Botanical Journal of the Linnean Society* 2003, **141:**399-436.

52. Phillips O, Baker T, Feldpausch T, Brienen R, Almeida S, Arroyo L, Aymard G, Chave J, Cardozo ND, Chao K-J, et al: **RAINFOR field manual for plot establishment and remeasurement.** Leeds, UK: RAINFOR; 2009.

53. Phillips OL, Aragão LEOC, Lewis SL, Fisher JB, Lloyd J, López-González G, Malhi Y, Monteagudo A, Peacock J, Quesada CA, et al: **Drought Sensitivity of the Amazon Rainforest.** *Science* 2009, **323:**1344-1347.

54. Tyree MT, Zimmermann MH: *Xylem structure and the ascent of sap. .* Berlin, Germany: Springer; 1983.

55. Koch GW, Sillett SC, Jennings GM, Davis SD: **The limits to tree height.** *Nature* 2004, **428:**851-854.

56. King DA, Davies SJ, Supardi MNN, Tan S: **Tree growth is related to light interception and wood density in two mixed dipterocarp forests of Malaysia.** *Functional Ecology* 2005, **19:**445-453.

57. Cairns MA, Brown S, Helmer EH, Baumgardner GA: **Root biomass allocation in the world's upland forests.** *Oecologia* 1997, **111:**1-11.

58. Poorter L, Hawthorne W, Bongers F, Sheil D: **Maximum size distributions in tropical forest communities: relationships with rainfall and disturbance.** *Journal of Ecology* 2008, **96:**495-504.

59. Poorter L, Bongers L, Bongers F: **Architecture of 54 Moist-Forest Tree Species: Traits, Trade-Offs, and Functional Groups.** *Ecology* 2006, **87:**1289-1301.

60. Larjavaara M, Muller-Landau HC: **Temperature explains global variation in biomass among humid old-growth forests.** *Global Ecology and Biogeography* 2012.

61. Clark DA, Piper SC, Keeling CD, Clark DB: **Tropical rain forest tree growth and atmospheric carbon dynamics linked to interannual temperature variation during 1984-2000.** *Proceedings of the National Academy of Sciences of the United States of America* 2003, **100:**5852-5857.

62. Slik JWF, Aiba S-I, Brearley FQ, Cannon CH, Forshed O, Kitayama K, Nagamasu H, Nilus R, Payne J, Paoli G, et al: **Environmental correlates of tree biomass, basal area, wood specific gravity and stem density gradients in Borneo's tropical forests.** *Global Ecology and Biogeography* 2010, **19:**50-60.

63. Clark DB, Clark DA, Oberbauer SF: **Annual wood production in a tropical rain forest in NE Costa Rica linked to climatic variation but not to increasing CO2.** *Global Change Biology* 2010, **16:**747-759.

64. Graham EA, Mulkey SS, Kitajima K, Phillips NG, Wright SJ: **Cloud cover limits net CO2 uptake and growth of a rainforest tree during tropical rainy seasons.** *Proceedings of the National Academy of Sciences* 2003, **100:**572-576.

65. Silvertown J: **Plant coexistence and the niche.** *Trends in Ecology &amp; Evolution* 2004, **19:**605-611.

66. Rüger N, Berger U, Hubbell SP, Vieilledent G, Condit R: **Growth Strategies of Tropical Tree Species: Disentangling Light and Size Effects.** *PLoS ONE* 2011, **6**.

67. Baltzer JL, Thomas SC, Nilus R, Burslem DFRP: **Edaphic Specialization In Tropical Trees: Physiological Correlates And Responses To Reciprocal Transplantation.** *Ecology* 2005, **86:**3063-3077.

68. Herwitz SR: **Growth Rates of Selected Australian Tropical Rainforest Tree Species under Controlled Conditions.** *Oecologia* 1993, **96:**232-238.

69. Way DA, Oren R: **Differential responses to changes in growth temperature between trees from different functional groups and biomes: a review and synthesis of data.** *Tree Physiology* 2010, **30:**669-688.

70. Stegen JC, Swenson NG, Enquist BJ, White EP, Phillips OL, Jørgensen PM, Weiser MD, Monteagudo Mendoza A, Núñez Vargas P: **Variation in above-ground forest biomass across broad climatic gradients.** *Global Ecology and Biogeography* 2011, **20:**744-754.

71. Meir P, Grace J: *The effects of drought on tropical forest ecosystems.* Oxford University Press; 2005.

72. Bunker DE, DeClerck F, Bradford JC, Colwell RK, Perfecto I, Phillips OL, Sankaran M, Naeem S: **Species loss and aboveground carbon storage in a tropical forest.** *Science* 2005, **310:**1029-1031.

73. Wiemann MC, Williamson GB: **Geographic variation in wood specific gravity: Effects of latitude, temperature, and precipitation.** *Wood and Fiber Science* 2002, **34:**96-107.

74. ter Steege H, Hammond DS: **Character Convergence, Diversity, And Disturbance In Tropical Rain Forest In Guyana.** *Ecology* 2001, **82:**3197-3212.

75. Doherty RM, Sitch S, Smith B, Lewis SL, Thornton PK: **Implications of future climate and atmospheric CO2 content for regional biogeochemistry, biogeography and ecosystem services across East Africa.** *Global Change Biology* 2009, **16:**617-640.

76. Sitch S, Huntingford C, Gedney N, Levy PE, Lomas M, Piao SL, Betts R, Ciais P, Cox P, Friedlingstein P, et al: **Evaluation of the terrestrial carbon cycle, future plant geography and climate-carbon cycle feedbacks using five Dynamic Global Vegetation Models (DGVMs).** *Global Change Biology* 2008, **14:**2015-2039.

77. Baker TR, Burslem DFRP, Swaine MD: **Associations between tree growth, soil fertility and water availability at local and regional scales in Ghanaian tropical rain forest.** *Journal of Tropical Ecology* 2003, **19:**109-125.

78. Asner GP, Alencar A: **Drought impacts on the Amazon forest: the remote sensing perspective.** *New Phytologist* 2010, **187:**569-578.

79. Kozlowski T: **Physiological-ecological impacts of flooding on riparian forest ecosystems.** *Wetlands* 2002, **22:**550-561.

80. Paoli G, Curran L, Slik J: **Soil nutrients affect spatial patterns of aboveground biomass and emergent tree density in southwestern Borneo.** *Oecologia* 2008, **155:**287-299.

81. DeWalt SJ, Chave J: **Structure and biomass of four lowland Neotropical forests.** *Biotropica* 2004, **36:**7-19.

82. Laurance WF, Fearnside PM, Laurance SG, Delamonica P, Lovejoy TE, Rankin-de Merona JM, Chambers JQ, Gascon C: **Relationship between soils and Amazon forest biomass: a landscape-scale study.** *Forest Ecology and Management* 1999, **118:**127-138.

83. van Schaik CP, Mirmanto E: **Spatial Variation in the Structure and Litterfall of a Sumatran Rain Forest.** *Biotropica* 1985, **17:**196-205.

84. Quesada CA, Lloyd J, Schwarz M, Baker TR, Phillips OL, Patiño S, Czimczik C, Hodnett MG, Herrera R, Arneth A, et al: **Regional and large-scale patterns in Amazon forest structure and function are mediated by variations in soil physical and chemical properties.** *Biogeosciences Discussions* 2009, **6:**3993-4057.

85. Quesada CA, Phillips OL, Schwarz M, Czimczik CI, Baker TR, Patiño S, Fyllas NM, Hodnett MG, Herrera R, Almeida S, et al: **Basin-wide variations in Amazon forest structure and function are mediated by both soils and climate.** *Biogeosciences* 2012, **9:**2203-2246.

86. Muller-Landau HC: **Interspecific and inter-site variation in wood specific gravity of tropical trees.** *Biotropica* 2004, **36:**20-32.

87. Woodcock DW: **Wood specific gravity of trees and forest types in the southern Peruvian Amazon.** *Acta Amazonica* 2000, **30:**589-599.

88. García-Oliva F, Masera OR: **Assessment and Measurement Issues Related to Soil Carbon Sequestration in Land-Use, Land-Use Change, and Forestry (LULUCF) Projects under the Kyoto Protocol.** *Climatic Change* 2004, **65:**347-364.

89. Phillips OL, van der Heijden G, Lewis SL, López-González G, Aragão LEOC, Lloyd J, Malhi Y, Monteagudo A, Almeida S, Dávila EA, et al: **Drought–mortality relationships for tropical forests.** *New Phytologist* 2010, **187:**631-646.

90. Larjavaara M, Muller-Landau HC: **Rethinking the value of high wood density.** *Functional Ecology* 2010, **24:**701-705.

91. West GB, Brown JH, Enquist BJ: **A general model for the structure and allometry of plant vascular systems.** *Nature* 1999, **400:**664-667.

92. Chave J, Condit R, Lao S, Caspersen JP, Foster RB, Hubbell SP: **Spatial and temporal variation of biomass in a tropical forest: results from a large census plot in Panama. .** *Journal of Ecology* 2003, **91:**240-252.

93. Giannini A, Biasutti M, Held I, Sobel A: **A global perspective on African climate.** *Climatic Change* 2008, **90:**359-383.

94. Lewis SL, Phillips OL, Baker TR, Lloyd J, Malhi Y, Almeida S, Higuchi N, Laurance WF, Neill DA, Silva JNM, et al: **Concerted changes in tropical forest structure and dynamics: evidence from 50 South American long-term plots.** *Philosophical Transactions of the Royal Society of London Series B-Biological Sciences* 2004, **359:**421-436.

95. Fisher JI, Hurtt GC, Thomas RQ, Chambers JQ: **Clustered disturbances lead to bias in large-scale estimates based on forest sample plots.** *Ecology Letters* 2008, **11:**554-563.

96. Lloyd J, Gloor EU, Lewis SL: **Are the dynamics of tropical forests dominated by large and rare disturbance events?** *Ecology Letters* 2009, **12:**E19-E21.

97. Lewis SL, Malhi Y, Phillips OL: **Fingerprinting the impacts of global change on tropical forests.** *Philosophical Transactions of the Royal Society of London Series B-Biological Sciences* 2004, **359:**437-462.

98. Lewis SL, Lloyd J, Sitch S, Mitchard ETA, Laurance WF: **Changing Ecology of Tropical Forests: Evidence and Drivers.** *Annual Review of Ecology, Evolution, and Systematics* 2009, **40:**529-549.

99. Platts PJ: **Spatial Modelling, Phytogeography and Conservation in the Eastern Arc Mountains of Tanzania and Kenya.** University of York, Environment Department 2012.

100. **High Resolution global Population Data Set copyrighted by UT-Battelle, LLC, operator of Oak Ridge National Laboratory under Contract No. DE-AC05-00OR22725 with the United States Department of Energy.** [<http://www.ornl.gov/sci/landscan/index.shtml>]

101. Platts PJ, Burgess ND, Gereau RE, Lovett JC, Marshall AR, Mcclean CJ, Pellikka PKE, Swetnam RD, Marchant R: **Delimiting tropical mountain ecoregions for conservation.** *Environmental Conservation* 2011, **38:**312-324.

102. de Castilho CV, Magnusson WE, de Araújo RNO, Luizão RCC, Luizão FJ, Lima AP, Higuchi N: **Variation in aboveground tree live biomass in a central Amazonian Forest: Effects of soil and topography.** *Forest Ecology and Management* 2006, **234:**85-96.

103. Batjes NH: **SOTER-based soil parameter estimates for Southern Africa.** vol. 4. pp. 27. Wageningen: ISRIC - World Soil Information; 2004:27.

104. ISRIC: **SOTER and WISE-based soil property estimates for Southern Africa. Available at** [**http://www.isric.org/UK/About+ISRIC/Projects/Track+Record/SOTWISsaf.htm**](http://www.isric.org/UK/About+ISRIC/Projects/Track+Record/SOTWISsaf.htm) **[Accessed 17/2/2010].** 2010.

105. Malhi Y, Baker TR, Phillips OL, Almeida S, Alvarez E, Arroyo L, Chave J, Czimczik CI, Fiore AD, Higuchi N, et al: **The above-ground coarse wood productivity of 104 Neotropical forest plots.** *Global Change Biology* 2004, **10:**563-591.

106. Matsumoto H: **Cell biology of aluminum toxicity and tolerance in higher plants.** In *International Review of Cytology.* *Volume* Volume 200: Academic Press; 2000: 1-46

107. Chazdon RL: **Tropical forest recovery: legacies of human impact and natural disturbances.** *Perspectives in Plant Ecology, Evolution and Systematics* 2003, **6:**51-71.

108. Omeja PA, Obua J, Rwetsiba A, Chapman CA: **Biomass accumulation in tropical lands with different disturbance histories: Contrasts within one landscape and across regions.** *Forest Ecology and Management* 2012, **269:**293-300.

109. Blanc L, Echard M, Herault B, Bonal D, Marcon E, Chave J, Baraloto C: **Dynamics of aboveground carbon stocks in a selectively logged tropical forest.** *Ecological Applications* 2009, **19:**1397-1404.

110. Ahrends A, Burgess ND, Milledge SAH, Bulling MT, Fisher B, Smart JCR, Clarke GP, Mhoro BE, Lewis SL: **Predictable waves of sequential forest degradation and biodiversity loss spreading from an African city.** *Proceedings of the National Academy of Sciences* 2010, **107:**14556-14561.

111. Macpherson AJ, Carter DR, Schulze MD, Vidal E, Lentini MW: **The sustainability of timber production from Eastern Amazonian forests.** *Land Use Policy* 2012, **29:**339-350.

112. Swetnam RD: **Historical logging in protected areas, E. Tanzania. V2.** Cambridge, UK: Zoology Department, Cambridge University; 2011.

113. IUCN, UNEP-WCMC: **The World Database on Protected Areas (WDPA). Cambridge, UK: UNEP- WCMC. Available at:** [**www.protectedplanet.net**](http://www.protectedplanet.net) **[Accessed 03/05/2010)]. .** 2010.

114. **Hole-filled SRTM for the globe Version 4, from the CGIAR-CSI SRTM 90m Database** [<http://srtm.csi.cgiar.org>]

115. Clark DA, Piper SC, Keeling CD, Clark DB: **Tropical rain forest tree growth and atmospheric carbon dynamics linked to interannual temperature variation during 1984-2000.** *Proceedings of the National Academy of Sciences of the United States of America* 2003, **100:**5852-5857.

116. Feeley KJ, Davies SJ, Perez R, Hubbell SP, Foster RB: **Directional changes in the species composition of a tropical forest.** *Ecology* 2011, **92:**871-882.

117. Zomer RJ, Trabucco A, Bossio DA, Verchot LV: **Climate change mitigation: A spatial analysis of global land suitability for clean development mechanism afforestation and reforestation.** *Agriculture, Ecosystems &amp; Environment* 2008, **126:**67-80.

118. **Tropical Rainfall Measuring Mission** [<http://trmm.gsfc.nasa.gov/>]

119. **Wind Speed At 50 m Above The Surface Of The Earth** [<http://eosweb.larc.nasa.gov/sse/>]

120. Roy DP, Jin Y, Lewis PE, Justice CO: **Prototyping a global algorithm for systematic fire-affected area mapping using MODIS time series data.** *Remote Sensing of Environment* 2005, **97:**137-162.

121. Slik J, Bernard C, Van Beek M, Breman F, Eichhorn K: **Tree diversity, composition, forest structure and aboveground biomass dynamics after single and repeated fire in a Bornean rain forest.** *Oecologia* 2008, **158:**579-588.

122. Balch JK, Nepstad DC, Curran LM, Brando PM, Portela O, Guilherme P, Reuning-Scherer JD, de Carvalho Jr O: **Size, species, and fire behavior predict tree and liana mortality from experimental burns in the Brazilian Amazon.** *Forest Ecology and Management* 2011, **261:**68-77.

123. Muoghalu JI: **Tree species population dynamics in a secondary forest at Ile-Ife, Nigeria after a ground fire.** *African Journal of Ecology* 2007, **45:**62-71.

124. Wright SJ, Calderón O: **Seasonal, El Ni&ntilde;o and longer term changes in flower and seed production in a moist tropical forest.** *Ecology Letters* 2006, **9:**35-44.

125. Perez R, Ineichen P, Moore K, Kmiecik M, Chain C, George R, Vignola F: **A new operational model for satellite-derived irradiances: description and validation.** *Solar Energy* 2002, **73:**307-317.

126. **Low Resolution Solar Data** [<http://www.nrel.gov/gis/>]

127. Dormann CF, McPherson JM, Araújo MB, Bivand R, Bolliger J, Carl G, Davies RG, Hirzel A, Jetz W, Daniel Kissling W, et al: **Methods to account for spatial autocorrelation in the analysis of species distributional data: a review.** *Ecography* 2007, **30:**609-628.

128. Maggini R, Lehmann A, Zimmermann NE, Guisan A: **Improving generalized regression analysis for the spatial prediction of forest communities.** *Journal of Biogeography* 2006, **33:**1729-1749.

129. Platts PJ, McClean CJ, Lovett JC, Marchant R: **Predicting tree distributions in an East African biodiversity hotspot: model selection, data bias and envelope uncertainty.** *Ecological Modelling* 2008, **218:**121-134.

130. Swetnam RD, Fisher B, Mbilinyi BP, Munishi PKT, Willcock S, Ricketts T, Mwakalila S, Balmford A, Burgess ND, Marshall AR, Lewis SL: **Mapping socio-economic scenarios of land cover change: A GIS method to enable ecosystem service modelling.** *Journal of Environmental Management* 2011, **92:**563-574.

131. Banin L: **Cross-continental comparisons of tropical forest structure and function.** Univeristy of Leeds, School of Geography 2010.

132. Huxley J: *Problems of relative growth.* New York: The Dial Press; 1932.

133. Enquist BJ: **Universal scaling in tree and vascular plant allometry: toward a general quantitative theory linking plant form and function from cells to ecosystems.** *Tree Physiology* 2002, **22:**1045-1064.

134. Meyer HA: **A Mathematical Expression for Height Curves.** *Journal of Forestry* 1940, **38:**415-420.

135. Fang Z, Bailey RL: **Height–diameter models for tropical forests on Hainan Island in southern China.** *Forest Ecology and Management* 1998, **110:**315-327.

136. Pinheiro JC, Bates DM: **Model building for nonlinear mixed-effects models.** In *Technical Report 931*. pp. 11: Department of Biostatistics, University of Wisconsin, Madison; 1994:11.

137. Winsor CP: **The Gompertz Curve as a Growth Curve.** *Proceedings of the National Academy of Sciences* 1932, **18:**1-8.

138. Richards FJ: **A Flexible Growth Function for Empirical Use.** *Journal of Experimental Botany* 1959, **10:**290-301.

139. Yang RC, Kozak A, Smith JHG: **The potential of Weibull-type functions as flexible growth curves.** *Canadian Journal of Forest Research* 1978, **8:**424-431.

140. Bailey RL: **The potential of Weibull-type functions as flexible growth curves: discussion.** *Canadian Journal of Forest Research* 1980, **10:**117-118.
