# Supplementary material for: Quantifying and understanding carbon storage and sequestration within the Eastern Arc Mountains of Tanzania, a tropical biodiversity hotspot
Source: Carbon Balance Manag. 2014 Apr 28;9:2. doi: 10.1186/1750-0680-9-2 (PMC4041645; doi:10.1186/1750-0680-9-2)

**Additional file 2: Figure S1** The spatial variation of WSG in tree-dominated land cover categories within the study area (a), with upper (b) and lower (c) pixel based 95% CI. See text for details on methods.


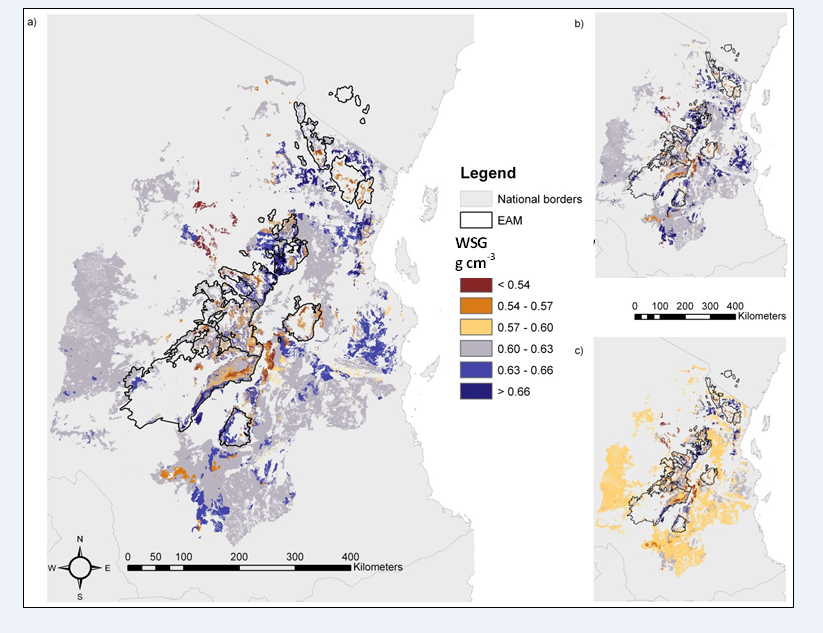

Supplement: Supplementary file 2 — Additional file 2: Figure S1: The spatial variation of WSG in tree-dominated land cover categories within the study area (a), with upper (b) and lower (c) pixel based 95% CI. See text for details on methods. (DOC 410 KB) [file 13021_2013_99_MOESM2_ESM.doc]
