# Supplementary material for: Quantifying and understanding carbon storage and sequestration within the Eastern Arc Mountains of Tanzania, a tropical biodiversity hotspot
Source: Carbon Balance Manag. 2014 Apr 28;9:2. doi: 10.1186/1750-0680-9-2 (PMC4041645; doi:10.1186/1750-0680-9-2)
Supplement: Supplementary file 3 — Additional file 3: Figure S2: The most influential, significant influential variables on WSG (a and b), the intercept of the power law relationship (c and d), and the gradient of the power law relationship (e and f). Dashed red lines indicate 95% CI. (DOC 77 KB) [file 13021_2013_99_MOESM3_ESM.doc]

**Additional file 3: Figure S2** The most influential, significant influential variables on WSG (a and b), the intercept of the power law relationship (c and d), and the gradient of the power law relationship (e and f). Dashed red lines indicate 95% CI.
