# Supplementary material for: Quantifying and understanding carbon storage and sequestration within the Eastern Arc Mountains of Tanzania, a tropical biodiversity hotspot
Source: Carbon Balance Manag. 2014 Apr 28;9:2. doi: 10.1186/1750-0680-9-2 (PMC4041645; doi:10.1186/1750-0680-9-2)

**Additional file 4: Figure S3** The spatial variation in the intercept of the power law relationship (a proxy measure for potential stem density) in tree dominated land cover categories within the study area (a), with upper (b) and lower (c) pixel based 95% CI. See text for details on methods.


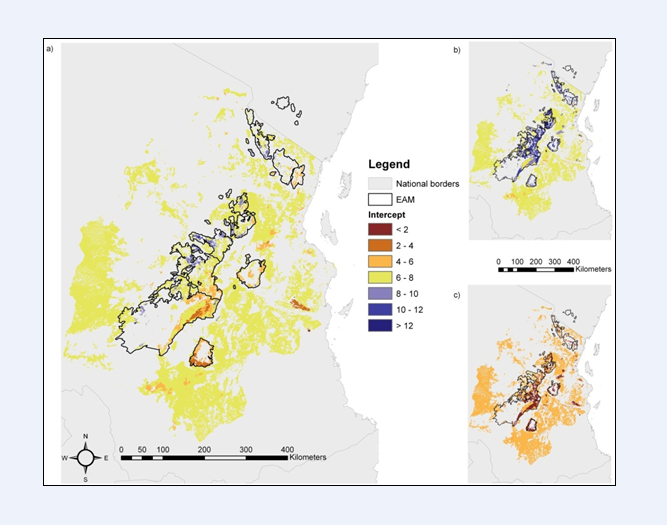

Supplement: Supplementary file 4 — Additional file 4: Figure S3: The spatial variation in the intercept of the power law relationship (a proxy measure for potential stem density) in tree dominated land cover categories within the study area (a), with upper (b) and lower (c) pixel based 95% CI. See text for details on methods. (DOC 273 KB) [file 13021_2013_99_MOESM4_ESM.doc]
