# Supplementary material for: Quantifying and understanding carbon storage and sequestration within the Eastern Arc Mountains of Tanzania, a tropical biodiversity hotspot
Source: Carbon Balance Manag. 2014 Apr 28;9:2. doi: 10.1186/1750-0680-9-2 (PMC4041645; doi:10.1186/1750-0680-9-2)

**Additional file 5: Figure S4** The spatial variation in the gradient of the power law relationship (a proxy measure for the proportion of larger stems) in tree-dominated land cover categories within the study area (a), with upper (b) and lower (c) pixel based 95% CI. See text for details on methods.


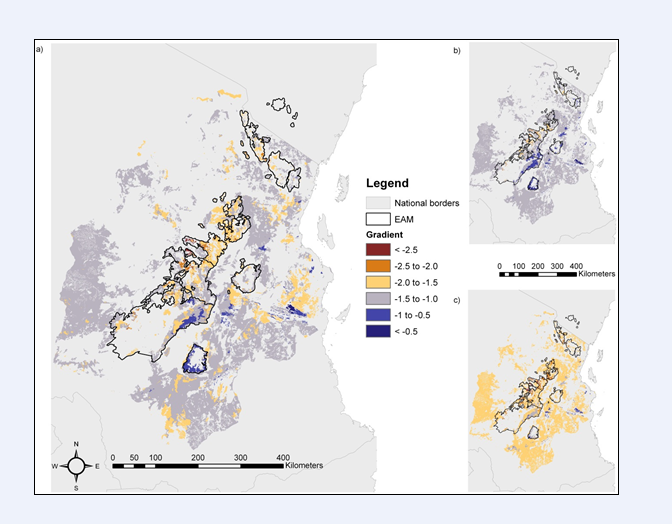

Supplement: Supplementary file 5 — Additional file 5: Figure S4: The spatial variation in the gradient of the power law relationship (a proxy measure for the proportion of larger stems) in tree-dominated land cover categories within the study area (a), with upper (b) and lower (c) pixel based 95% CI. See text for details on methods. (DOC 254 KB) [file 13021_2013_99_MOESM5_ESM.doc]
