# Supplementary material for: Quantifying and understanding carbon storage and sequestration within the Eastern Arc Mountains of Tanzania, a tropical biodiversity hotspot
Source: Carbon Balance Manag. 2014 Apr 28;9:2. doi: 10.1186/1750-0680-9-2 (PMC4041645; doi:10.1186/1750-0680-9-2)
Supplement: Supplementary file 6 — Additional file 6: Figure S5: The 2nd order interactions relating my carbon storage derivatives (wood specific gravity, maximum canopy height, the intercept of the power law relationship, and the gradient of the power law relationship [shown here as WSG, height, intercept, and gradient respectively]) to aboveground live carbon storage. Dashed red lines indicate 95% CI. (DOC 91 KB) [file 13021_2013_99_MOESM6_ESM.doc]

**Additional file 6: Figure S5** The 2^nd^ order interactions relating my carbon storage derivatives (wood specific gravity, maximum canopy height, the intercept of the power law relationship, and the gradient of the power law relationship [shown here as WSG, height, intercept, and gradient respectively]) to aboveground live carbon storage. Dashed red lines indicate 95% CI.
