# Supplementary material for: Quantifying and understanding carbon storage and sequestration within the Eastern Arc Mountains of Tanzania, a tropical biodiversity hotspot
Source: Carbon Balance Manag. 2014 Apr 28;9:2. doi: 10.1186/1750-0680-9-2 (PMC4041645; doi:10.1186/1750-0680-9-2)

**Additional file 7: Figure S6** The effect of MAT on tree height for a range of DBH. The data (points) correspond to DBH ranges whereas the Gompertz model fits (solid lines) illustrate the relationship for mid-point of this range only. Dotted lines represent the 95CI of the model fits.


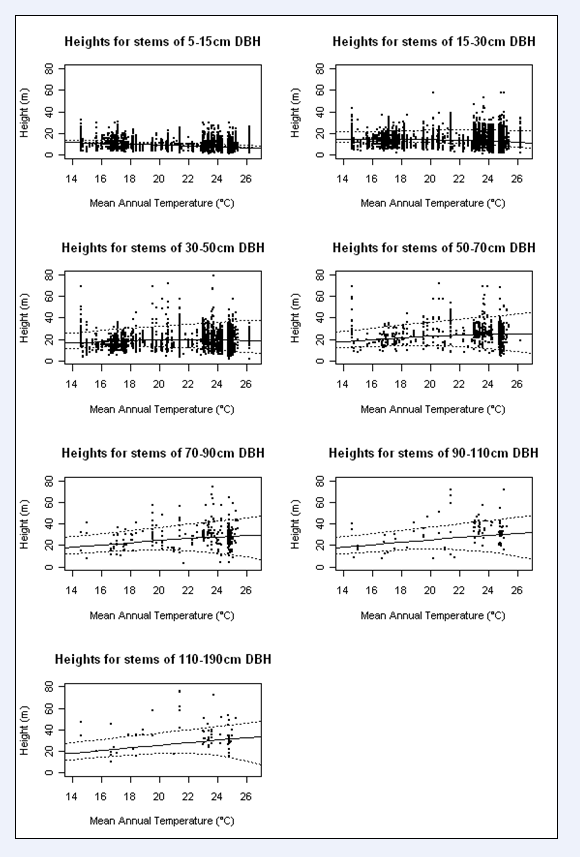

Supplement: Supplementary file 7 — Additional file 7: Figure S6: The effect of MAT on tree height for a range of DBH. The data (points) correspond to DBH ranges whereas the Gompertz model fits (solid lines) illustrate the relationship for mid-point of this range only. Dotted lines represent the 95CI of the model fits. (DOC 122 KB) [file 13021_2013_99_MOESM7_ESM.doc]
