# Supplementary figures and images for: Quantifying and understanding carbon storage and sequestration within the Eastern Arc Mountains of Tanzania, a tropical biodiversity hotspot
Source: Carbon Balance Manag. 2014 Apr 28;9:2. doi: 10.1186/1750-0680-9-2 (PMC4041645; doi:10.1186/1750-0680-9-2)

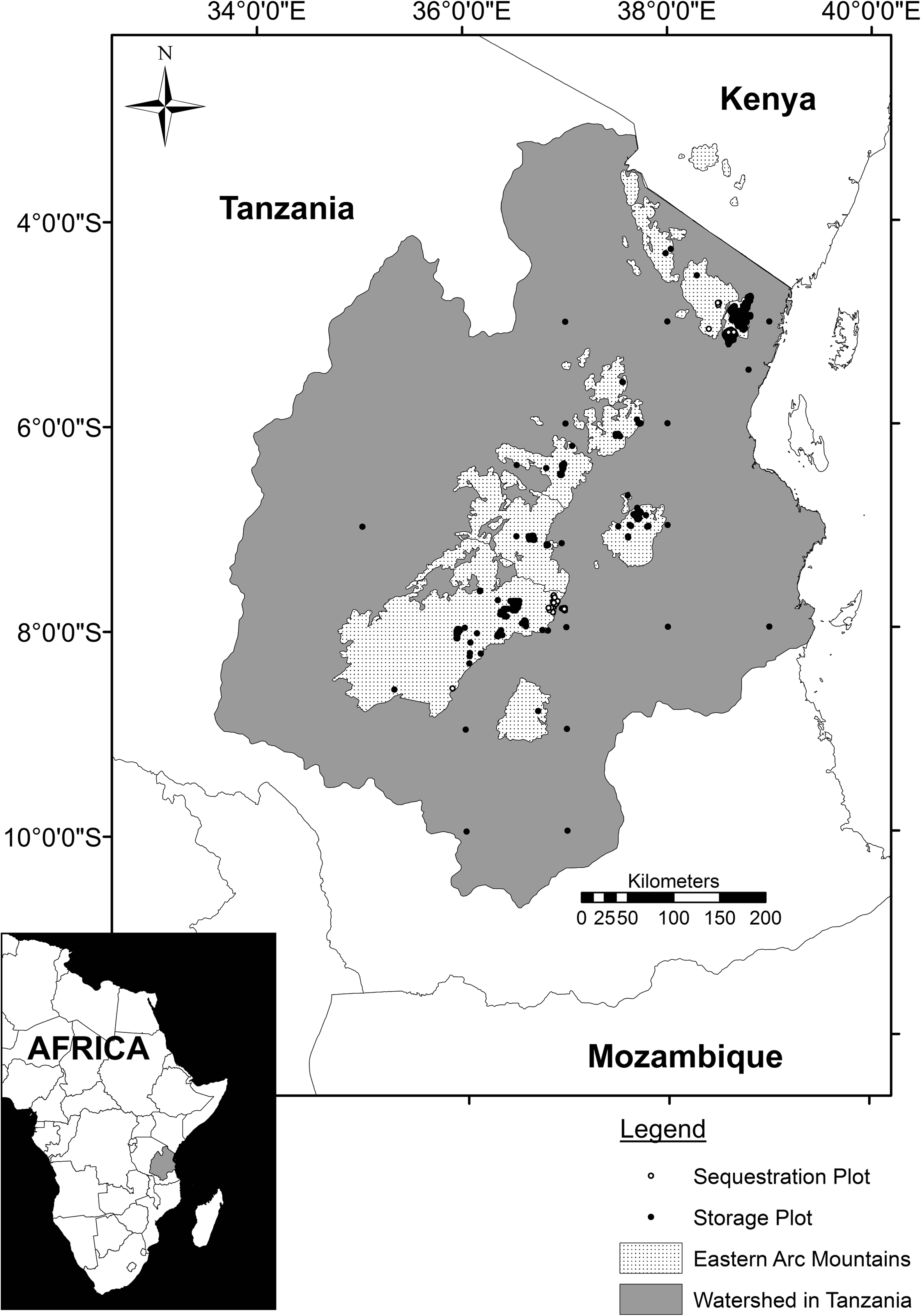

Supplement: Supplementary file 8 — Authors’ original file for figure 1 [file 13021_2013_99_MOESM8_ESM.tiff]

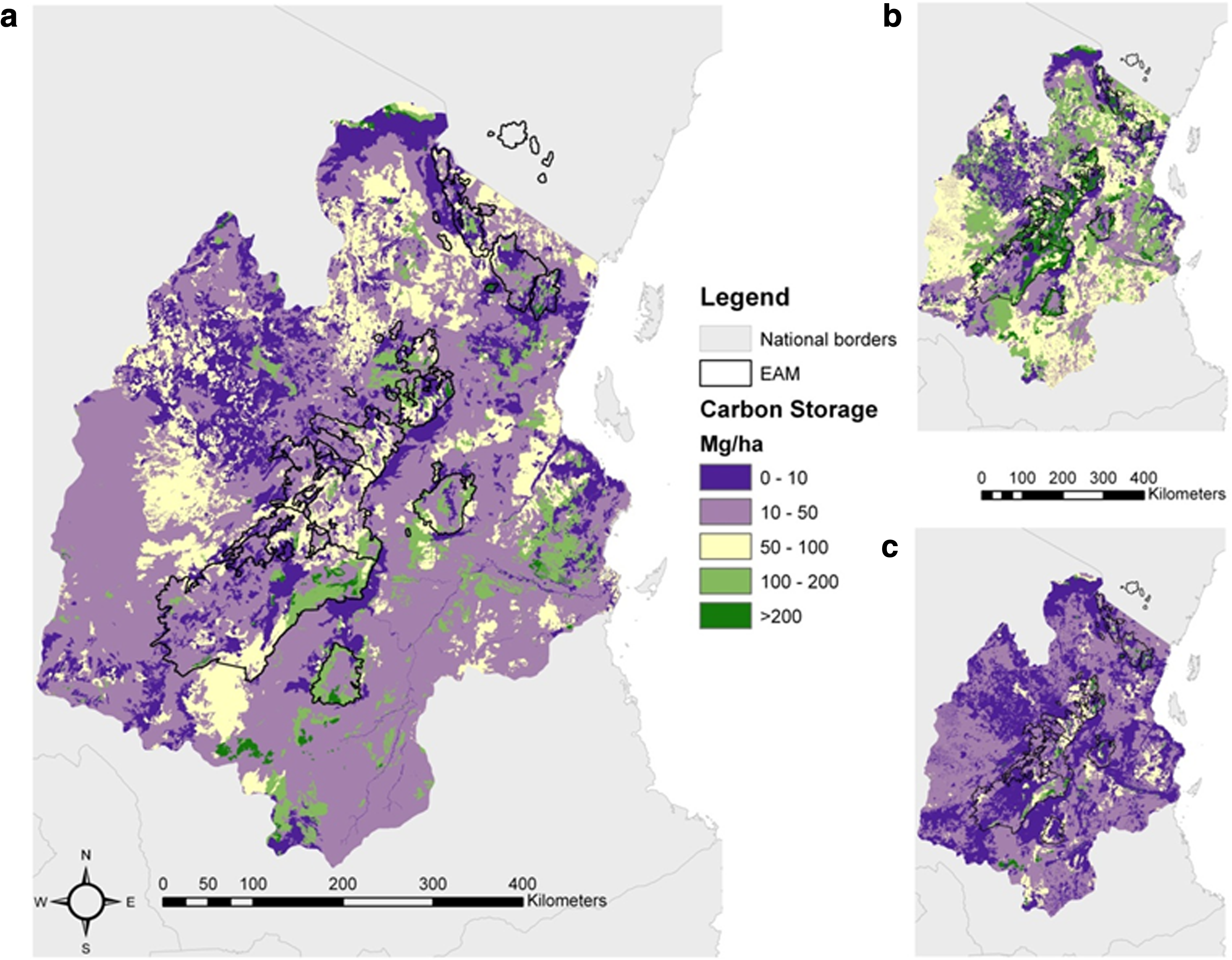

Supplement: Supplementary file 9 — Authors’ original file for figure 2 [file 13021_2013_99_MOESM9_ESM.tiff]

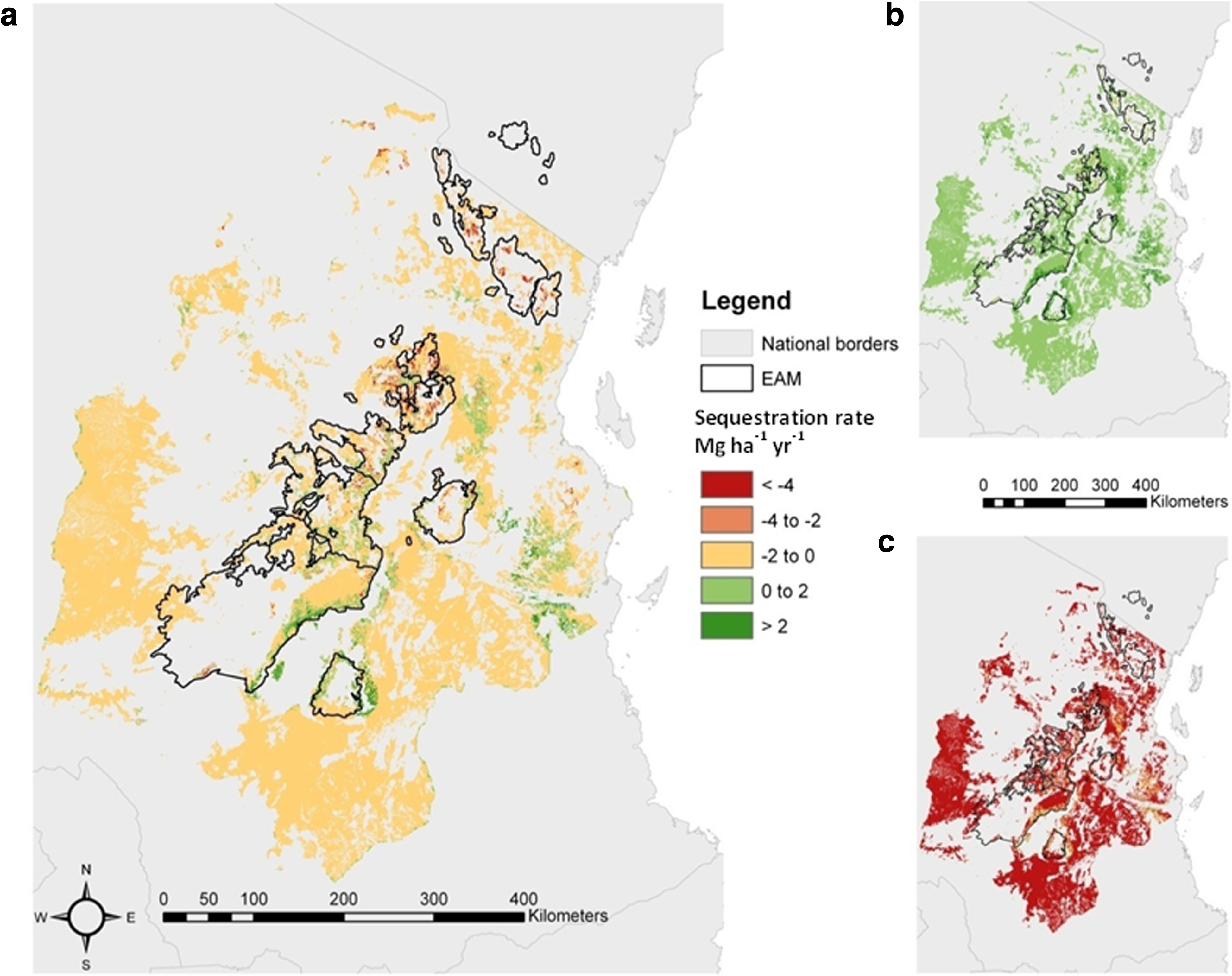

Supplement: Supplementary file 10 — Authors’ original file for figure 3 [file 13021_2013_99_MOESM10_ESM.tiff]

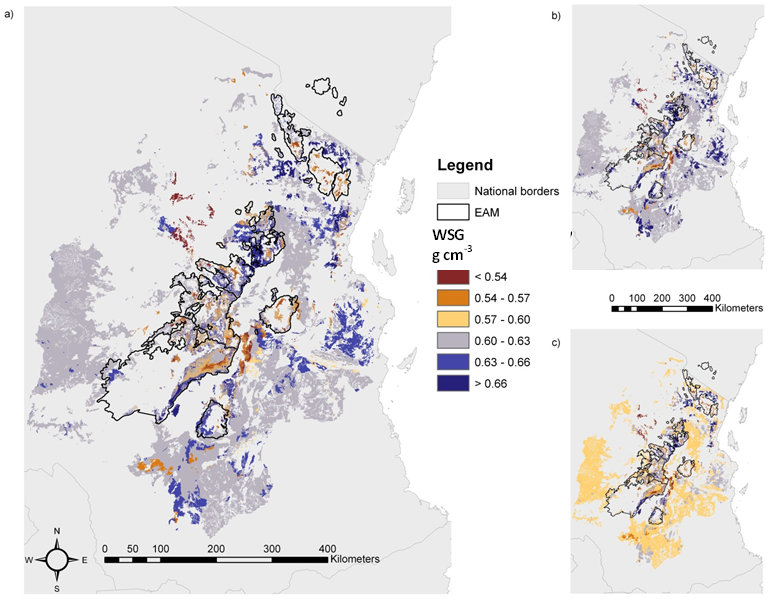

Supplement: Supplementary file 12 — Authors’ original file for figure 5 [file 13021_2013_99_MOESM12_ESM.png]

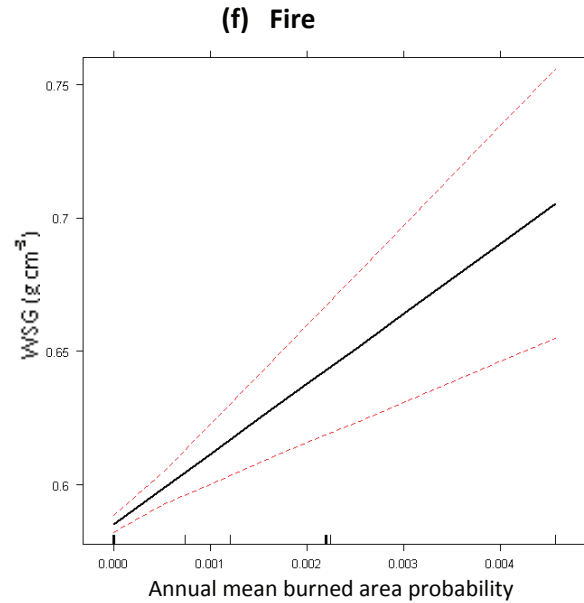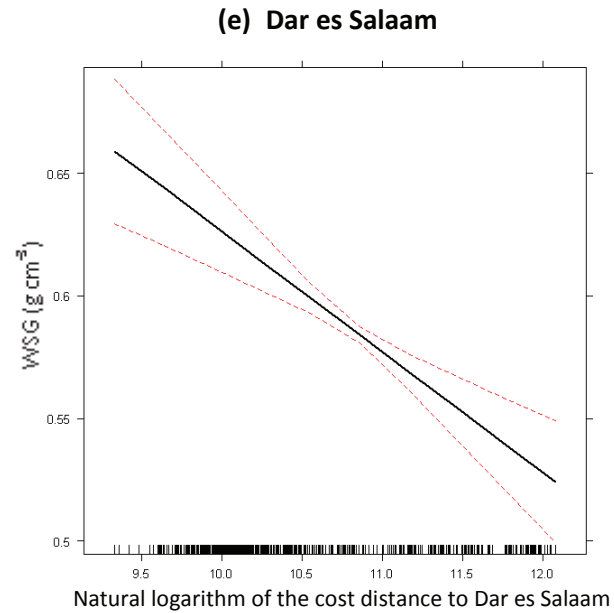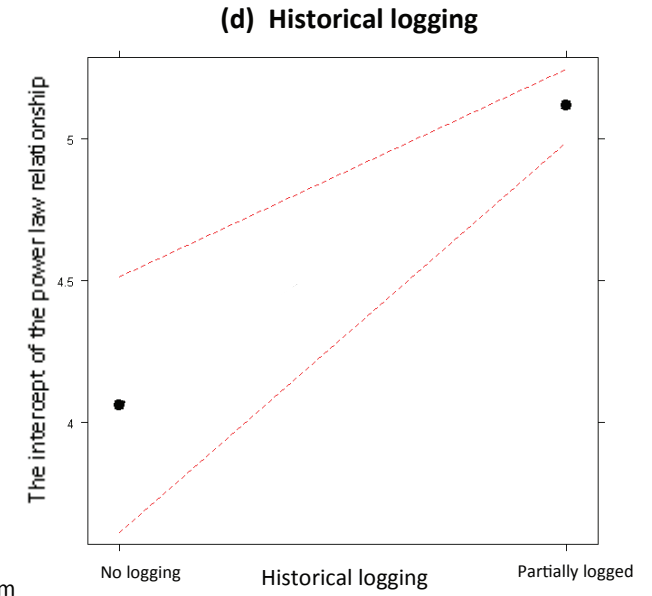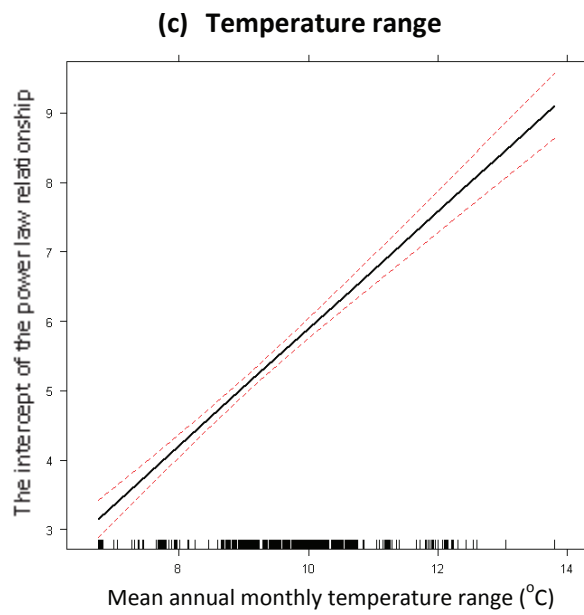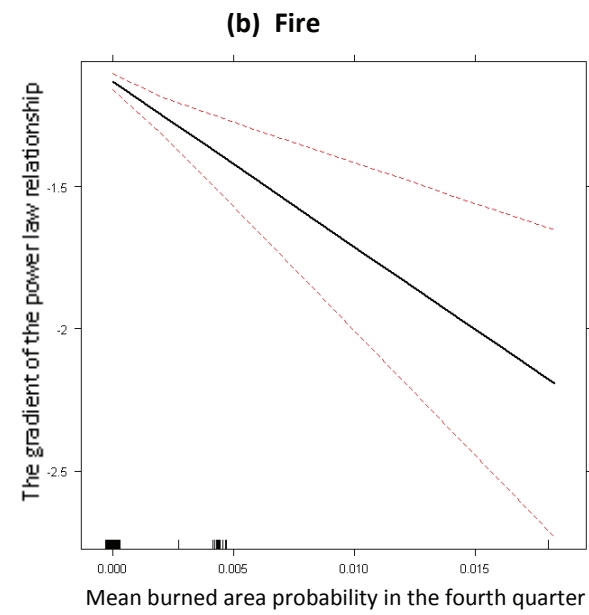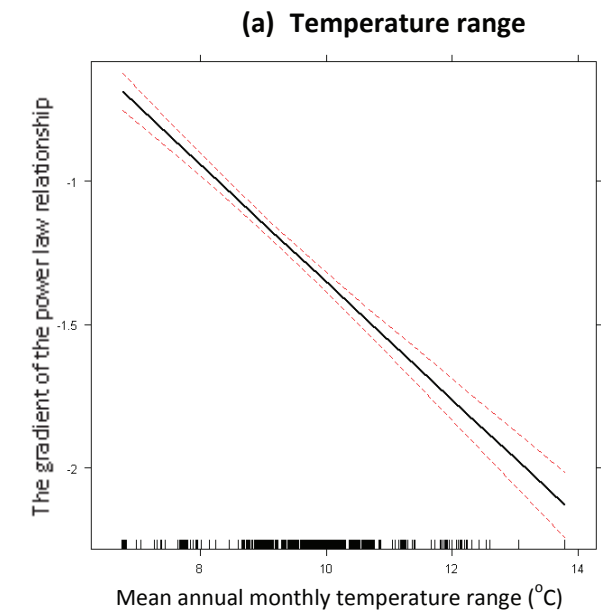

Supplement: Supplementary file 13 — Authors’ original file for figure 6 [file 13021_2013_99_MOESM13_ESM.pdf]

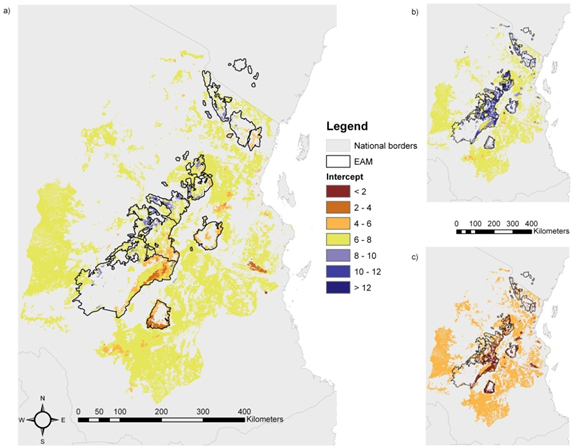

Supplement: Supplementary file 14 — Authors’ original file for figure 7 [file 13021_2013_99_MOESM14_ESM.png]

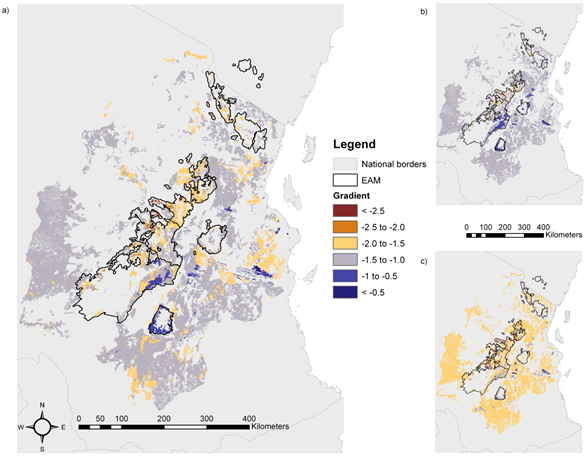

Supplement: Supplementary file 15 — Authors’ original file for figure 8 [file 13021_2013_99_MOESM15_ESM.png]

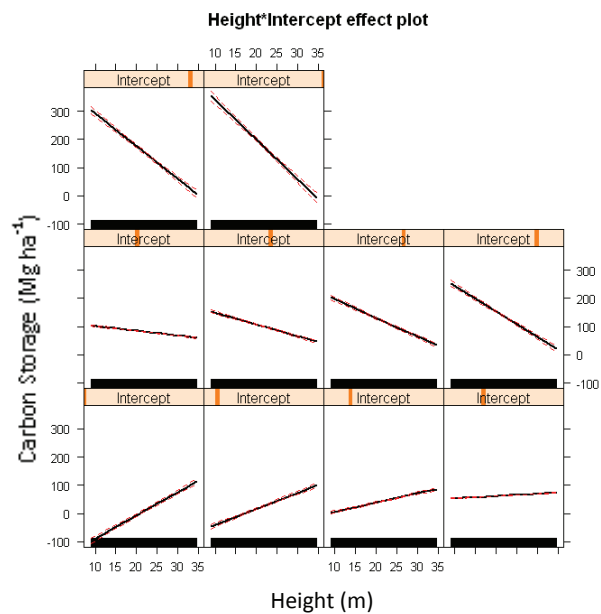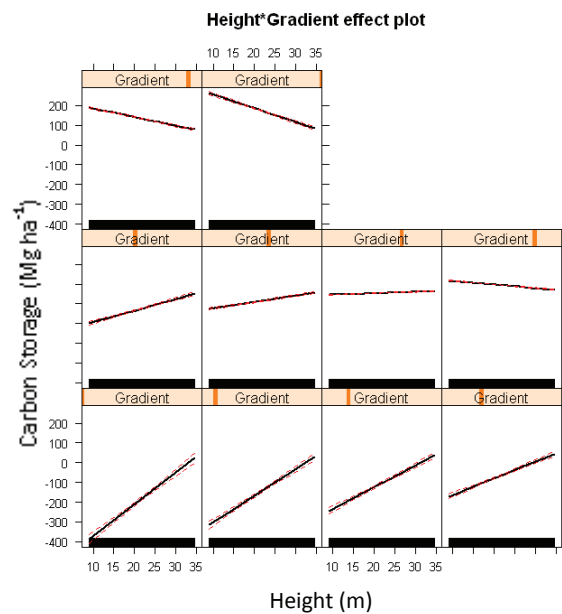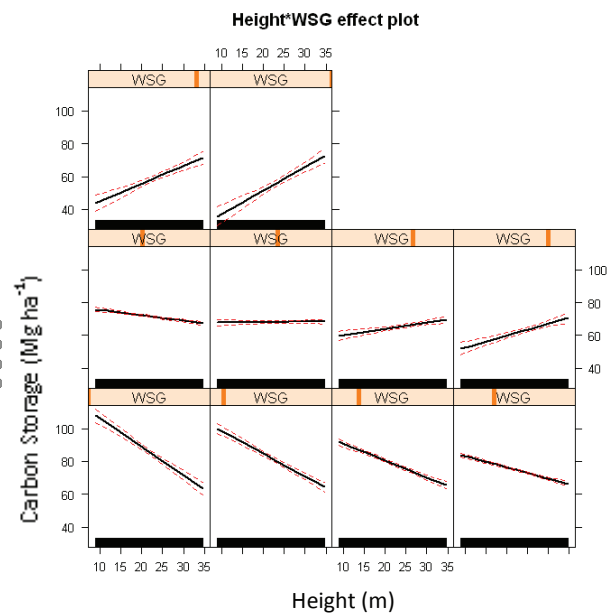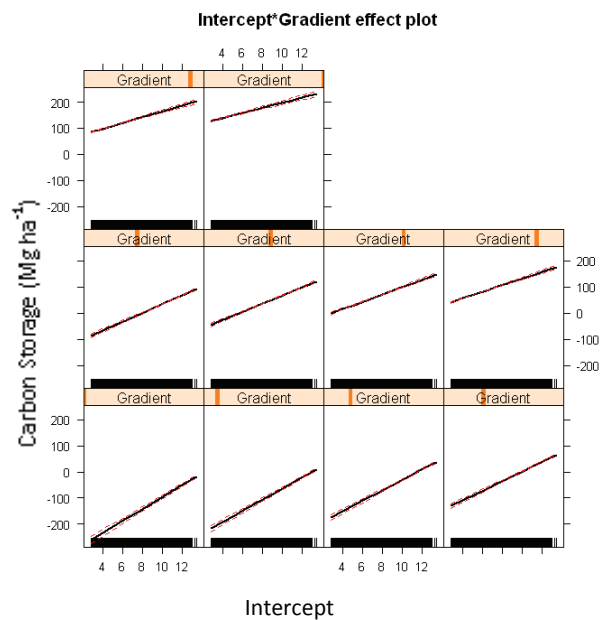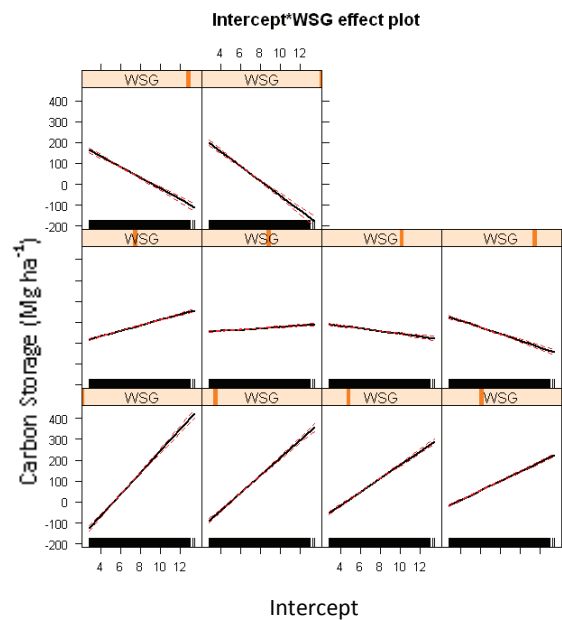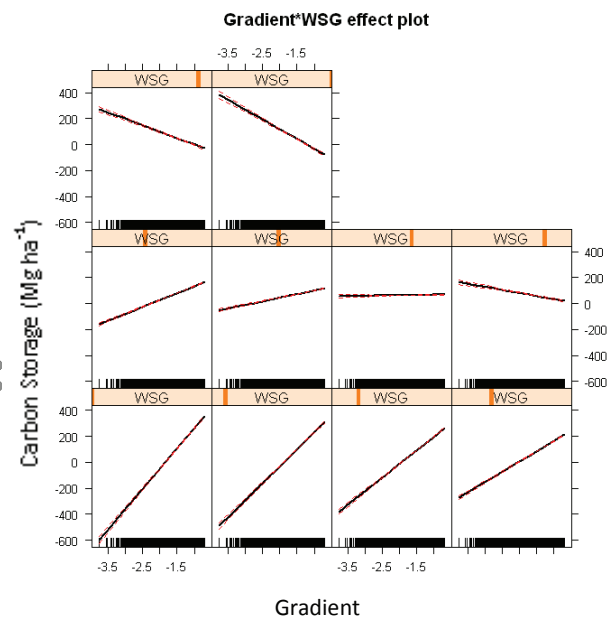

Supplement: Supplementary file 16 — Authors’ original file for figure 9 [file 13021_2013_99_MOESM16_ESM.pdf]

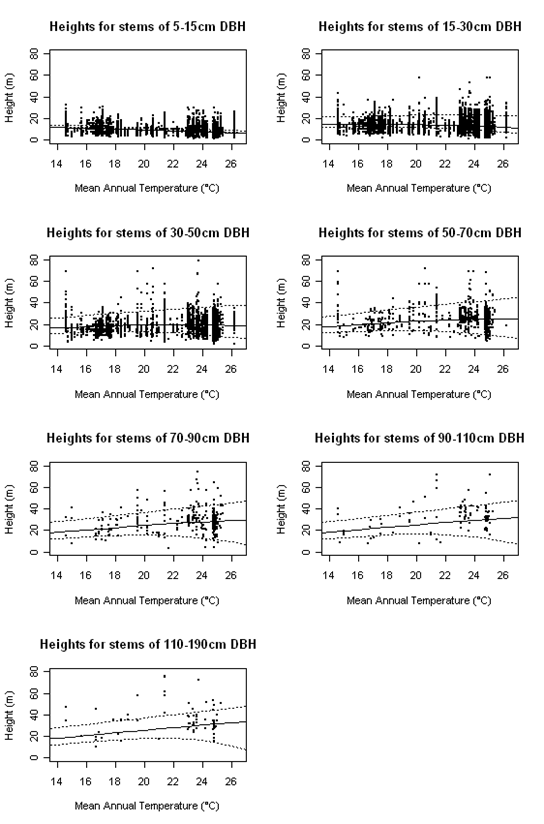

Supplement: Supplementary file 17 — Authors’ original file for figure 10 [file 13021_2013_99_MOESM17_ESM.png]
