# Supplementary material for: Quantifying and understanding carbon storage and sequestration within the Eastern Arc Mountains of Tanzania, a tropical biodiversity hotspot
Source: Carbon Balance Manag. 2014 Apr 28;9:2. doi: 10.1186/1750-0680-9-2 (PMC4041645; doi:10.1186/1750-0680-9-2)
Supplement: Supplementary file 11 — Authors’ original file for figure 4 [file 13021_2013_99_MOESM11_ESM.pdf]

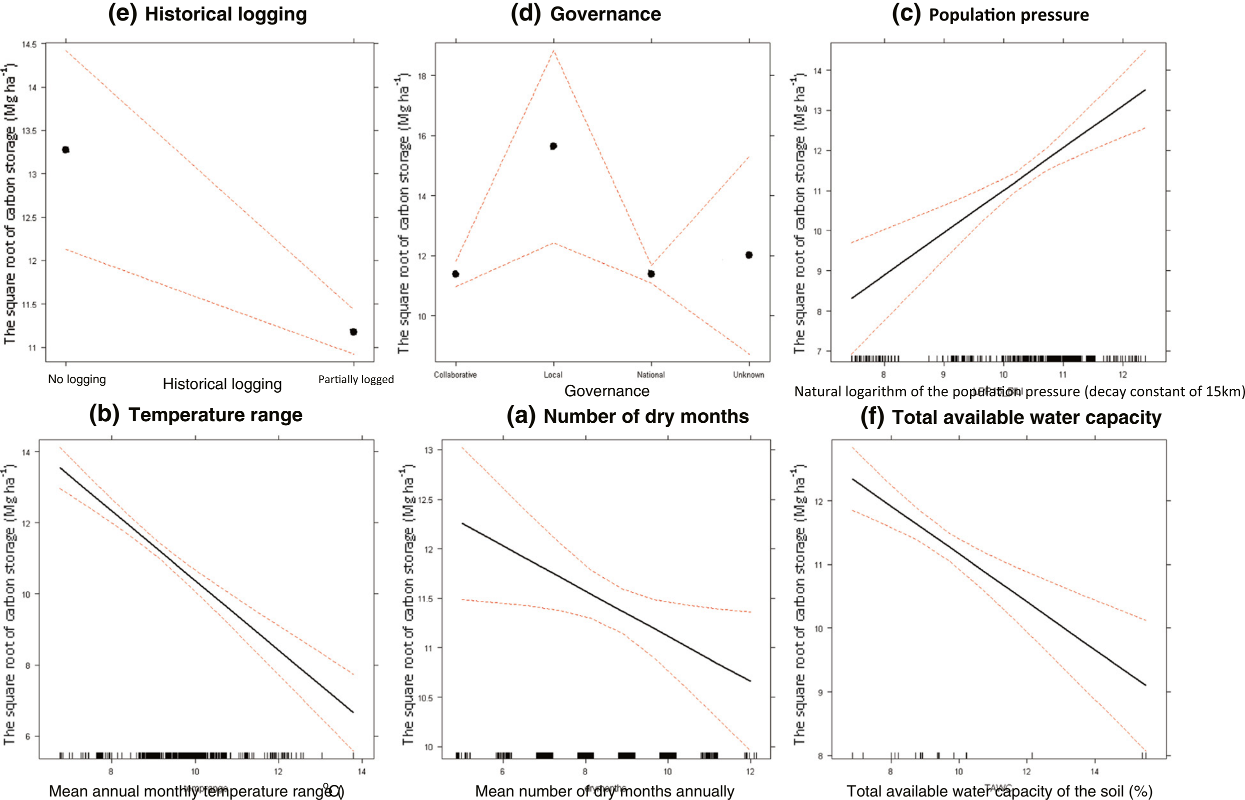
(e) Historical logging
The square root of carbon storage (Mg ha-1)
No logging
Historical logging
Partially logged
(d) Governance
Collaborative
Local
National
Unknown
Governance
(c) Population pressure
Natural logarithm of the population pressure (decay constant of 15km)
(b) Temperature range
Mean annual monthly temperature range (°C)
(a) Number of dry months
Mean number of dry months annually
(f) Total available water capacity
Total available water capacity of the soil (%)
